# Hominin Variability and Evolutionary Relationships at Guattari Cave During the Middle and Late Pleistocene (San Felice Circeo, Latina, Italy)

**DOI:** 10.3390/genes17020132

**Published:** 2026-01-26

**Authors:** Mauro Rubini, Paola Zaio, Ferdinando Spanό, Flavio Cognigni, Marco Rossi, Alessandro Gozzi, Francesco Di Mario

**Affiliations:** 1 Former Superintendence of Archaeology, Fine Arts and Landscape for the provinces of Frosinone and Latina, 00100 Rome, Italy; antropologiasal@libero.it (M.R.); alessandrogozzi89@gmail.com (A.G.); francescodimario@gmail.com (F.D.M.); 2 Istituto Italiano di Paleontologia Umana, Piazza Ruggero Bonghi 2, 03012 Anagni, Italy; 3 Departmental Doctoral Program in Ancient Civilizations, University of Basel, 4051 Basel, Switzerland; 4 Emergency Diagnostics, Policlinico Umberto I, University of Rome “LaSapienza”, 00100 Rome, Italy; ferdinando.spano@uniroma1.it; 5 Carl Zeiss S.p.A, Research Microscopy Solutions, Via Varesina, 162, 20156 Milan, Italy; flavio.cognigni@zeiss.com; 6 Department of Basic and Applied Sciences for Engineering (SBAI), University of Rome La Sapienza, Via Antonio Scarpa 14, 00161 Rome, Italy; marco.rossi@uniroma1.it

**Keywords:** neanderthal evolution, middle/late Pleistocene, hominin remains, Guattari Cave, Italian peninsula archeology

## Abstract

**Background/Objectives:** Along the Tyrrhenian coast of central Italy, multilayered caves have yielded significant Neanderthal-era human remains. Recent excavations at Guattari Cave uncovered hominin fossils dated to approximately 66–65 ka, revealing a population with notable morpho-anatomical variability exhibiting both *plesiomorphic* (primitive) and *autapomorphic* (derived) traits. **Methods:** Here we present detailed morphometric and comparative analyses of cranial, dental, and postcranial remains, demonstrating affinities with *Homo erectus* (*sensu stricto* [*s.s.*] and *lato* [*s.l.*]), Proto-Neanderthals, classical Neanderthals, and *Homo sapiens*. **Results:** These findings indicate notable morpho-anatomical variability among the Guattari Cave hominin remains, with affinities to multiple hominin lineages during the Middle and Late Pleistocene. Pleistocene. **Conclusions:** The Guattari Cave assemblage thus contributes to our understanding of Eurasian hominin diversity and evolutionary dynamics, highlighting the Mediterranean as a region of interest for studying the phyletic continuity and diversity preceding modern humans.

## 1. Introduction

**Figure 1:**
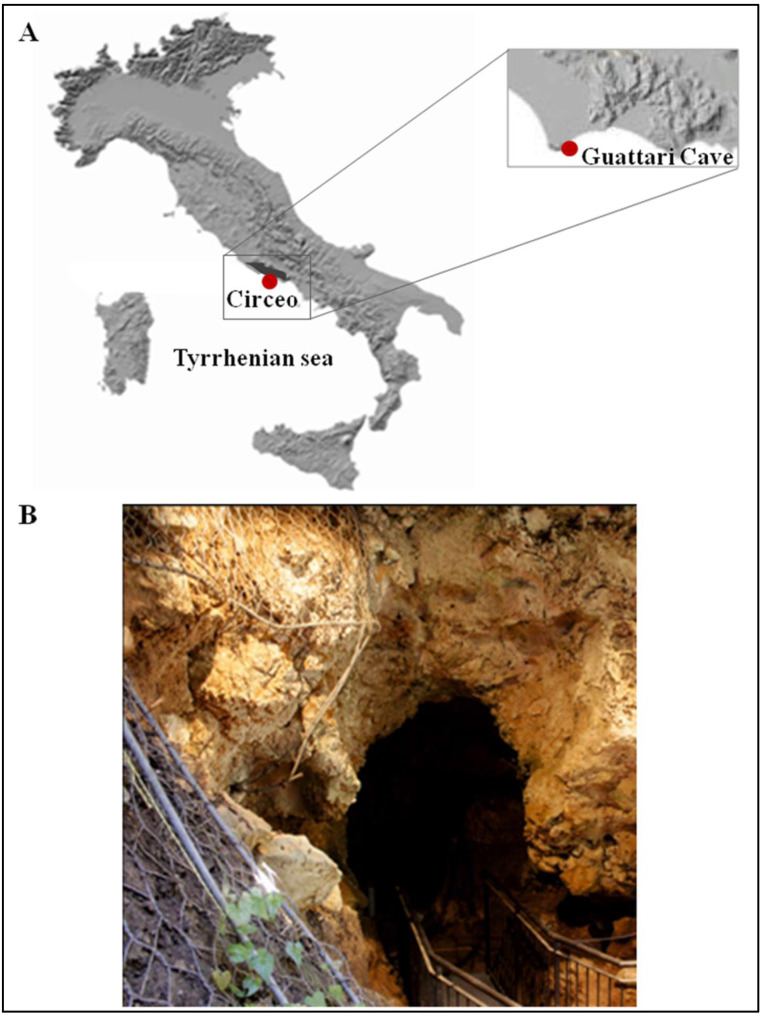
(**A**) Location of Guattari Cave (map of digital elevation). (**B**) The cave entrance.

In recent years, significant progress has been made in understanding human populations during the Middle and Late Pleistocene. Advances in dating techniques, as well as paleoenvironmental, paleoecological, and genetic reconstructions, have enabled a more detailed reconstruction of population movements and interactions during this period, shedding new light on key aspects of human evolution. During the Pleistocene, Neanderthals coexisted in Eurasia with other hominin species, including “*H. erectus*”, “*Homo heidelbergensis*”, Denisovans (from the Altai and southern Siberia) [1], and “*H. sapiens*”, as well as more recently discovered or debated species such as “*Homo luzonensis*”, “*Homo floresiensis*”, and the controversial “*Homo longi*”. Throughout their long history, Neanderthals, like other contemporary human groups, faced abrupt climatic and environmental changes [2,3,4] and adapted to these challenges by seeking regions with favorable climates and abundant resources. As a result, they dispersed widely across Eurasia, from Gibraltar to the Altai Mountains, and from the Middle East to Britain and the Mediterranean. The Tyrrhenian coast of Lazio (central Italy) features numerous caves between the Circeo and Gaeta promontories, many of which show evidence of human activity; however, only three—Fossellone Cave, Breuil Cave, and Guattari Cave—have yielded human remains. Guattari Cave is located approximately one hundred meters from the Tyrrhenian Sea, on the eastern side of the Circeo promontory (Figure 1). In the first half of the twentieth century, a complete Neanderthal skull (Circeo 1) and two mandibles (Circeo 2 and Circeo 3) were discovered there by chance [5,6]. More recently, archeological excavations conducted between 2019 and 2022 uncovered an additional 15 human remains, representing a remarkable addition to the Italian Middle Pleistocene record and making a significant contribution to European paleoanthropological research.

### The Role of Morphology in the Absence of Genomic Data

In contexts lacking ancient DNA (aDNA), such as the specimens examined in this study, morphological analysis represents the primary tool for phylogenetic reconstruction and taxonomic delimitation.

In this study, a comparative morphometric analysis was conducted to identify morphological divergences and affinities along the entire evolutionary lineage of hominins, starting from a phylogenetic hypothesis that reconciles some genetic results reported in the recent literature.

Morphology provides observable and quantifiable characteristics (traits) that, when properly coded, can be used in cladistic analyses to infer evolutionary relationships. In the absence of molecular data, this approach becomes essential for systematics and evolutionary reconstruction.

Application scenarios include the following:

- Fossil species and extinct taxa: In paleontological specimens, DNA is generally degraded or absent, as often occurs in fossil remains found along the Italian coastal area. Morphology therefore becomes the only source of information for placing fossils within phylogenetic trees.
- Functional and ecological analysis: Morphological structures reflect functional adaptations and selective pressures, providing clues about ecology and behavior.

The main advantages of morphometric analysis are universality, applicability to both living and fossil taxa, and operational immediacy. Conversely, the limitations include evolutionary convergence, phenotypic plasticity, and intraspecific variability, which can reduce phylogenetic resolution.

In the absence of molecular data, morphology is not merely an alternative but an indispensable component for systematics and evolutionary reconstruction. Integrating morphological characteristics with ecological and stratigraphic data helps mitigate intrinsic limitations and achieve more robust inferences.

## 2. Archeological Background

**Figure 2:**
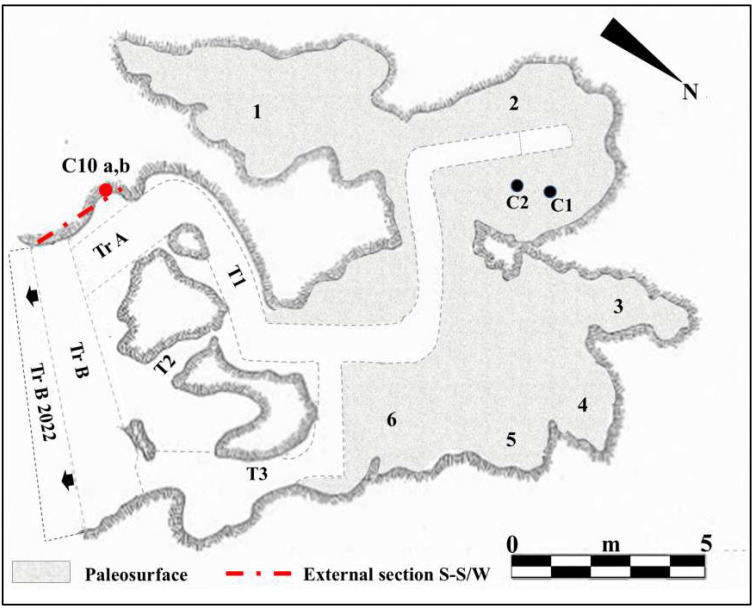
Planimetry of Guattari Cave. Internal area of the cave showing the paleosurface of level 2. **1.** “Antro del Laghetto”; **2.** “Antro dell’Uomo” with the localization of the human specimens: Circeo 1 (**C1**) skull and Circeo 2 (**C2**) mandible, found in 1939 (**black circles**); **3.** “Antro del Bue”; **4.** “Antro della Iena”; **5.** “Antro del Rinoceronte”; **6.** “Antro del Cervo”. External area of the cave: recently investigated sector (**black arrows**), trench B (**Tr B 2022**). Trenches A and B (**Tr A** and **Tr B**) and tunnels 1, 2, and 3 (**T1-3**), historical excavation Blanc, Cardini, and Segre of the 1930s and 1940s. Section S-S/W highlighted by Blanc in 1939 [5] and recently investigated (mixed line with two dots and long red line) with localization of the human specimen, Circeo 10 (**C10**, **red circle**), found during the cleaning investigations. Modified after Segre [9] and Rolfo et al. [10].

The entrance to Guattari Cave is located approximately 7 m above sea level on the eastern slopes of Monte Morrone, a low hill on the Circeo promontory (Figure 1 and Supplementary Figure S1). The cave was discovered by chance on 24 February 1939, during construction work. In an internal chamber later named “Antro dell’Uomo”, a Neanderthal skull and mandible (Circeo 1, C1 and Circeo 2, C2) were found resting on an ancient ground surface (paleosurface). A second Neanderthal mandible, Circeo 3 (C3) [7,8], was discovered outside the cave in 1950. Segre [9] reported that the human remains were located near a deposit of bone-rich breccia (ossiferous breccia) still attached to the rock face above the entrance to tunnel 1 (Figure 2). Excavations began immediately after the initial discovery and continued, with varying intensity, until the 1950s, involving both the external and internal areas of the cave (Supplementary Figure S2) through the creation of several trenches (Figure 2). Throughout this area, excavations appear to have reached the Tyrrhenian fossil beach at the base of the Pleistocene sequence, extending more than a meter below the original surface.

### 2.1. The Excavation of the Internal Deposit “Antro Del Laghetto”

**Figure 3:**
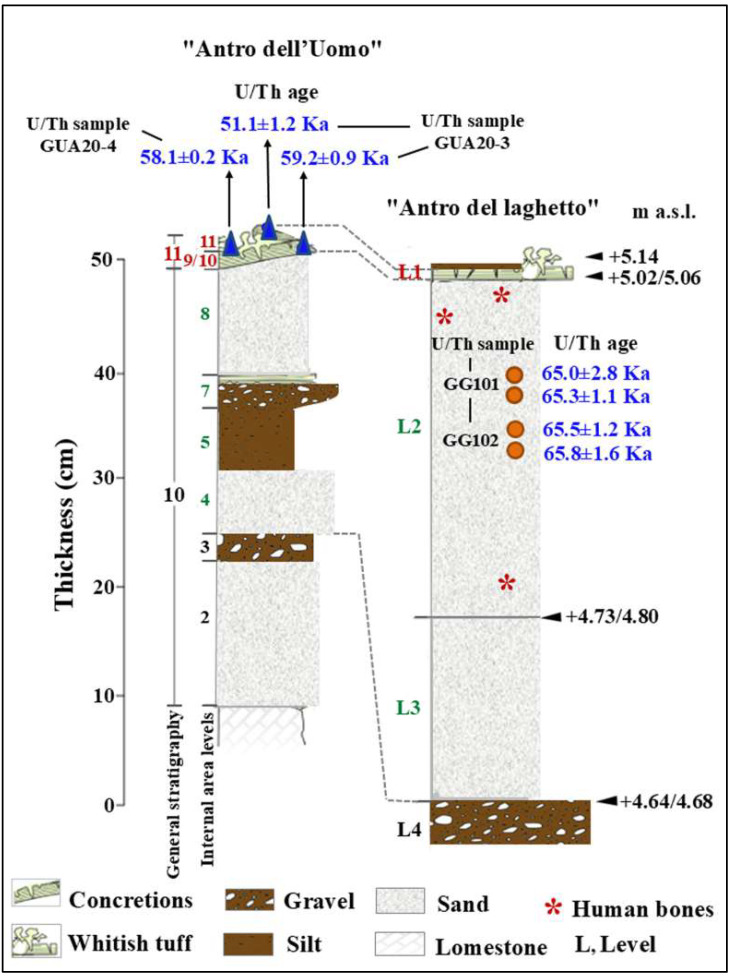
Stratigraphy of the “Antro dell’Uomo” and “Antro del laghetto”. Modified after Segre [9] and Rolfo et al. [10]. **L1/11**. Tuffaceous coralloid concretion and whitish tuff and bone partially covered by the flowstone; **8** and **4**. Mammal bone and coprolites; **L2**. Stalagmite concretions, pebbles of limestone, mammal bones, coprolites, lithic artifacts, and human bones (red asterisk); **L3**. Tuffaceous flowstone, mammal bones, coprolites, and lithic artifacts.

On 11 October 2019, under the direction of the Superintendence of Archaeology, Fine Arts and Landscape for the provinces of Frosinone and Latina, new systematic research began in the Guattari Cave, which focused mainly on the area called “Antro del Laghetto” (due to the presence of a small accumulation of water, especially in the winter season). The excavation encompassed the entire stratigraphic deposit, which consists of a complex succession of overlapping layers with a thickness ranging from 47 to 102 cm. A total of 25 Stratigraphic Units (SUs) were identified, allowing the distinction of four different levels, each with distinct geological and archeological characteristics (Figure 3).

**Level 1.** The surface layer corresponds to the stalagmitic crust, of modest thickness, that covers the entire surface of the “Laghetto”, formed over time by the precipitation of *calcium* carbonate from the groundwater that seasonally floods the area.

**Level 2.** Layer characterized by a paleosurface, with an average thickness of 40 cm, with skeletal remains distributed randomly over the entire investigated area.

**Level 3.** This level is strongly altered by phosphatization, resulting in faunal remains that are badly damaged by the chemical alteration of the sediment. Lithic artifacts were also recovered from this level; the lithic industry present, known as the Pontinian, represents a regional variant of the Mousterian stone tool tradition [11,12].

**Level 4**. Although this was the last level to be excavated, it is not the final layer in the stratigraphic sequence. It consists of a series of stalagmitic crusts of varying development, some of which have been altered by chemical processes affecting the *calcium* carbonate. From an archeological perspective, this level is sterile, meaning it contains no evidence of human activity.

According to Rolfo et al. [10], the low overall number of lithic artifacts in the “Antro del Laghetto” succession (28 in level 3 and 7 in level 2) suggests a limited human presence inside the cave. In contrast, evidence of human activity is well documented in the more external and older deposits [9,10].

The paleosurface of level 2 of the “Laghetto” area, unlike the other areas of the cave, was not visible because it was covered and incorporated, in its superficial portion, by the level 1 stalagmitic concretion.

**Figure 4:**
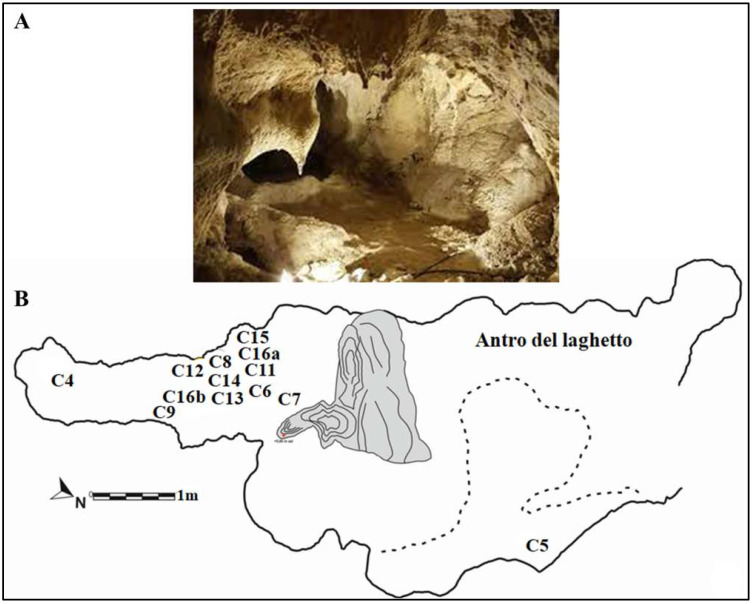
(**A**) “Antro del Laghetto”. (**B**) Planimetry showing the paleosurface of “Antro del Laghetto” level 2. Localization of the human specimens (C*n*) discovered during recent excavations. They are all stratigraphically placed within level 2. Modified after Rolfo et al. [10].

**Figure 5:**
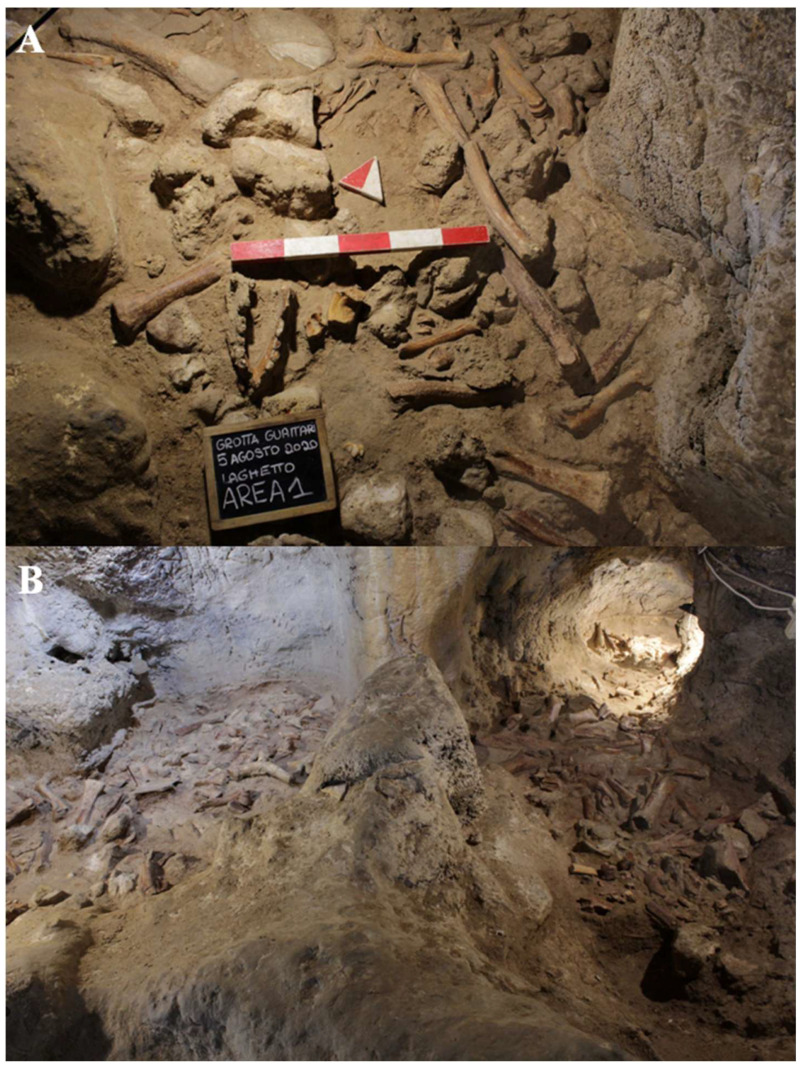
Level 2: (**A**) paleosurface of the “Laghetto” area; (**B**) paleosurface of the cave.

**Figure 6:**
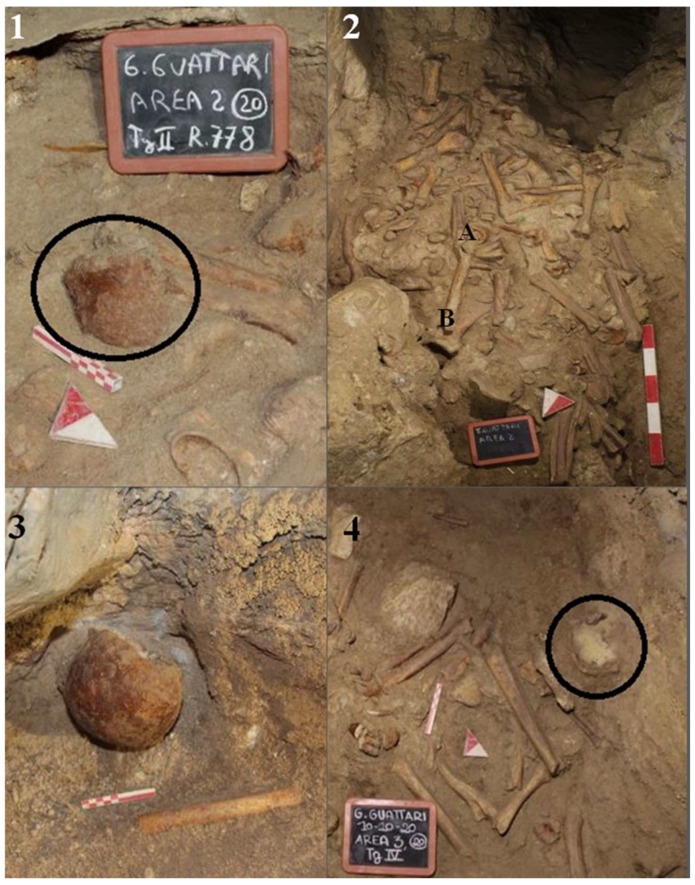
Level 2 human remains under excavation. **1**, One of the two hemifrontals of Circeo 4; **2**, Mandible, Circeo 6 (**A**), and femur, Circeo 11 (**B**); **3**, *Calvarium* Circeo 5; **4**, Occipital bone, Circeo 8 (black circle).

During the excavation of level 2, several human remains (Figure 4B) were found scattered among the faunal remains in various areas of the “Laghetto,” all located on the paleosurface (Figure 5). A significant number of human skeletal fragments were recovered, and images documenting the discovery of some of these are presented in Figure 6.

It is still unclear whether the abundance of bone remains, which in some areas form accumulations over 60 cm thick, is the result of direct predatory activity by animals or the natural movement of cave deposits, causing material to slide from northeast to southwest.

The faunal remains, found together with the human remains, which characterize the entire paleosurface of level 2 of the cave (Figure 5), include red deer (*Cervus elaphus*), which is the dominant species, followed by the spotted hyena (*Crocuta crocuta*) and the aurochs (*Bos primigenius*). Other identified species include wild horse *(Equus ferus*) and wild boar (*Sus scrofa*), rare fallow deer (*Dama dama*), two bear species (*Ursus spelaeus* and *Ursus arctos*), a few remains of a rhinoceros species (probably *Stephanorhinus hemitoechus*), abundant giant deer (the Irish elk, *Megaloceros giganteus*), rare elephant (*Palaeoloxodon antiquus*) and hippopotamus (*Hippopotamus amphibius*), roe deer (*Capreolus capreolus*), leopard (*Panthera pardus*), ibex (*Capra ibex*), chamois (*Rupicapra* sp.), cave lion (*Panthera spelaea*), wild cat (*Felis silvestris*), European hemione (*Equus hydruntinus*), hare (*Lepus sp*.), fox (*Vulpes vulpes*) and wolf (*Canis lupus*), and at least one mustelid in addition to sparse birds and micromammals [13].

### 2.2. The Excavation of the External Deposit

Archeological investigations were also carried out outside the cave, focusing on the residual portions of the sections left by the Blanc excavation of 1939–1950 and on the limited areas spared by past excavations. The new interventions began with the cleaning and documentation of the sections highlighted by Blanc in 1939, both the one identified as section S-S/W, and the east and west sections of trench B (Figure 2).

**Figure 7:**
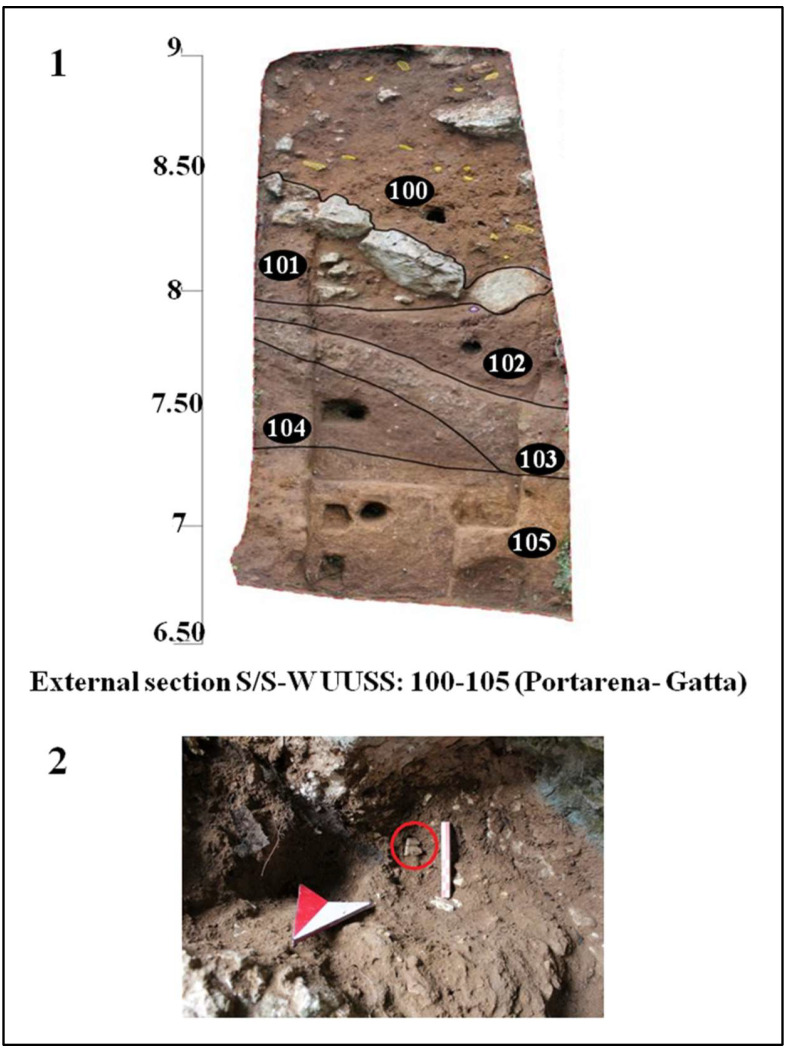
External area. **1**, Section S/S/W; **2**, detail of the excavation of the two human teeth (red circle, Circeo 10) in the south section.

The aim of the investigations was to highlight and analyze the stratigraphic sequence of the external deposit (Figure 7):

(a) A first level containing faunal remains and collapse blocks of part of the outermost portion of the cave vault. The discovery of two human teeth (Circeo 10 a, b) belonging to the same individual is worth noting (Figure 2 and Figure 7).
(b) A very compact underlying level concretioned with rare lithic industry.
(c) A compact sandy level with rare lithic industry.

Excavation of the remaining portions of the deposit in front of the cave, which were spared by previous investigations, has confirmed the findings of earlier excavations: a series of levels with a high concentration of charcoal, faunal remains, and lithic artifacts. The entire stratigraphic complex, which is over 50 cm deep, is characterized by abundant faunal remains (many of which are burnt) and a high concentration of charcoal and lithic industry [9,10], with geochronological data placing these deposits between approximately 121 ka and 105 ka [14].

The discovery of charcoal and burnt animal bones suggests the presence of a structured hearth, with burnt soil fragments, in the immediate vicinity. These findings indicate an initial and oldest phase of structured human activity in the atrial portion and the external area in front of the cave, particularly in the sector in front of trench B (Figure 2). In contrast, a second and much later phase of hominin presence is documented mainly by the occurrence of human skeletal remains, found among numerous faunal remains, in the internal area of the cave.

### 2.3. Chronology

**Figure 8:**
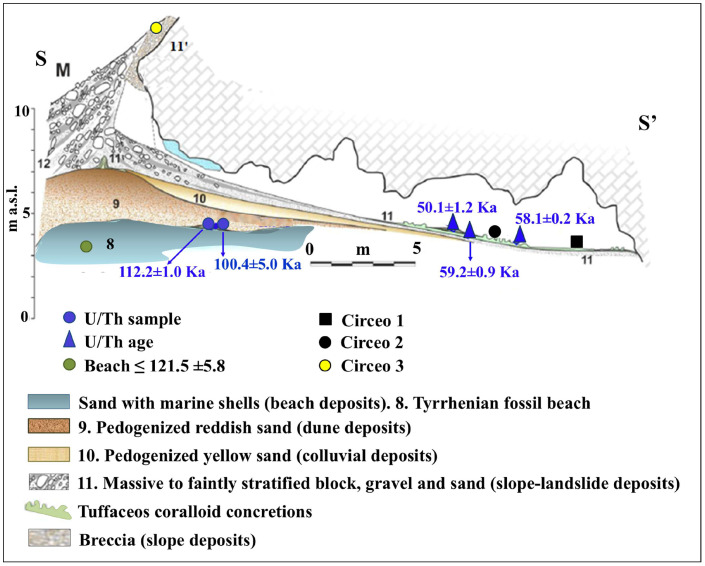
Simplified geological section of Guattari Cave, with the localization of the historical remains and of the U/Th samples described in the text. Green circle (level 8), 40Ar/39Ar dating of detrital sanidine, extracted from the biodetritic deposit occurring at the base of the cave fill (Stratigraphic Unit 10 [SU10]), in Marra et al. [11], yielded ages of 121 ± 5 ka. Modified after Segre [9] and Rolfo et al. [10].

**Figure 9:**
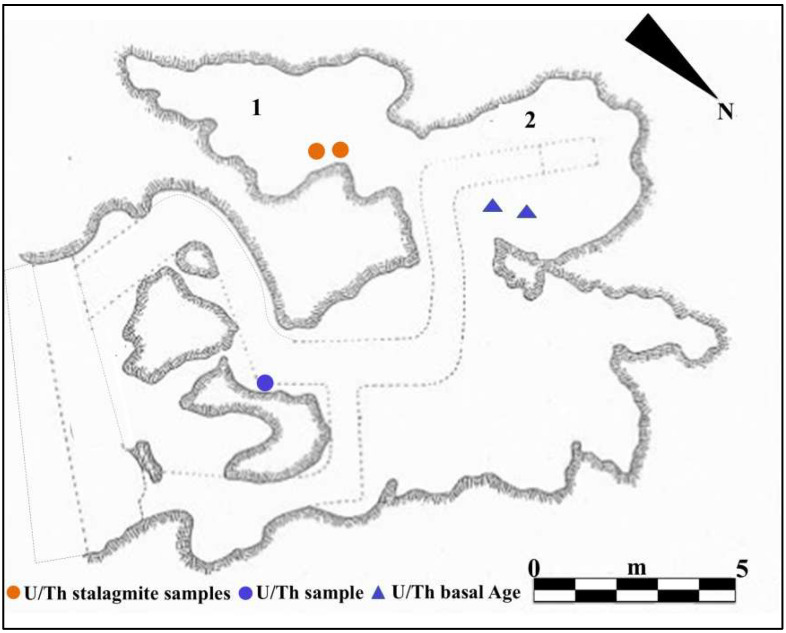
U/Th geochronology (method applied to corals or speleothems): from the “Antro del Laghetto”, (**1**) two stalagmite samples (**orange circles**); from the “Antro dell’Uomo”, (**2**) two samples of the surface coralloid concretion (**blue triangles**); basal age of the internal continental infilling of the cave, U/Th samples of the basal marine deposit (**blue circle**). Modified after Segre [9] and Rolfo et al. [10].

The general stratigraphy of the cave, as summarized by Segre [9], divides the internal and external deposits into several units, with the lowest unit consisting of fossil beach deposits (Figure 8 and Figure 9). The attribution of this deposit to Marine Isotope Stage (MIS) 5.5 was recently confirmed by 40Ar/39Ar dating of detrital sanidine extracted (Figure 8) from the biodetritic deposit at the base of the cave fill, which yielded an age of 121.5 ± 5.8 ka [10,11]. The basal age of the internal continental fill, determined from a sample of the layer covering the basal marine deposit (Figure 8 and Figure 9), provided two dates: 112.6 ± 0.9 ka and 100.4 ± 5.9 ka [10]. Therefore, the entire internal continental clastic succession of the cave fill developed between the age of the flow overlying the beach deposits (112.2 ± 1.0 ka to 100.4 ± 5.0 ka) and before 59.9 ± 0.8 ka, the age of the oldest coralloid concretions [10,15].

For the geochronology of the “Antro del Laghetto”, two stalagmites were selected within level 2, with accumulation of human and animal bones, and two samples were taken of coralloid calcitic concretion which covers the upper surface of the “Antro dell’Uomo” [10] (Figure 3 and Figure 9). The coralloid concretion taken from the skull of C1 (“Antro dell’Uomo”) was previously dated, with the isochronous U/Th method, by Schwarcz et al. [15].

The results indicate that the middle-upper succession of the “Antro del Laghetto” (level 2) dates to approximately 66–65 ka (MIS 4), while the upper portion of the “Antro dell’Uomo” has a basal age of 59.17 ± 0.85 ka and a maximum age of 51.1 ± 1.2 ka [10] (Figure 3). Overall, the new chronological data for the surface coralloid concretion are consistent with the earlier dating of approximately 57–50 ka by Schwarcz et al. [15]. The middle-upper part of the “Antro del Laghetto” is precisely dated by four concordant U/Th measurements to a narrow interval of ~66–65 ka, corresponding to the Greenland Stadial (GS) 19.1 [10,16], one of the most severe periods of the MIS 4 glacial stage. In regions such as Apulia, Abruzzo, and southern Italy, this interval coincides with drier, more open, and arid conditions [10]. In summary, the stratigraphic sequence of Guattari Cave was formed between MIS 5.5 and MIS 3, that is, between the high Tyrrhenian marine stand (~125 ka) and the landslide that closed the cave after 50 ka [17]. Thus, like other coastal caves affected by sea-level fluctuations, Guattari Cave may have become accessible to humans from the end of MIS 5.5, around 120 ka [18].

## 3. Materials and Methods

Guattari Cave has yielded numerous human remains alongside a large assemblage of fauna through both historical and recent excavations. The bones, found in a randomly mixed context, were initially treated as a single assemblage. Specimens were separated into faunal and human remains, with all human skeletal elements identified, cataloged by element type, and accompanied by all available contextual information. Unidentified specimens were excluded from analysis. To preserve the integrity of the remains, restoration and removal of concretions were not performed.

The overall number of human fossil remains, first and second discoveries, consist of 18 cranial and postcranial remains. The laboratory code adopted was C (Circeo) and the numbering, progressive, starts taking into account the findings of 1939–1950 (C1, C2, C3). Analysis of the minimum number of individuals (NMI) was carried out on the most represented bone segment (skull). The result identifies the presence of at least four individuals (C1, C4, C5, and C8). The skeletal remains from the recent discovery (2019–2022) include a fronto-parietal portion, “Circeo 4” (C4); a *calvarium*, “Circeo 5” (C5); a mandible incomplete, “Circeo 6” (C6); a femoral diaphysis, “Circeo 7” (C7); an occipital bone, “Circeo 8” (C8); an incomplete upper maxillary, “Circeo 9” (C9); and finally a right coxal bone, “Circeo 16 b” (C16b), and a left, “Circeo 16 a” (C16a), incomplete and probably belonging to two different individuals female. Presence/absence of pathological conditions, trauma, or taphonomic changes were observed. The mixed nature of the discovery led to a comparative analysis of the remains, among themselves, visually assessing characteristics such as shape, size, tissue proportions, developmental stage, and presence of anatomical features and pathological conditions. This assessment revealed no obvious links. The new human remains are described and compared with the human remains from 1939–1950 and with a large sample of diachronic and synchronic *Homo* fossils from the Pleistocene (Supplementary Table S1) distributed over different geographical areas (Europe, Africa, and Asia) to evaluate phenetic affinities and evolutionary relationships. The presence/absence of non-metric morphological traits presented in Supplementary Table S2 and described in the text was detected.

The skeletal measurements of the hominins (Supplementary Tables S3–S10) were taken with composite digital calipers following the suggestions of Martin [19]. Sex of Circeo 16 (a, b) was determined according to the standards of Buikstra and Ubelaker [20].

CT analysis of the Circeo 7 femur was performed at Policlinico Umberto I, University of Rome “La Sapienza,” using a 64-slice CT scanner. Acquisition parameters included 20 × 0.625 detectors, a rotation time of 0.75 s, and pitch of 0.3, 760 mAs, and 140 kV. Reconstruction parameters were a thickness of 0.67 mm, increment of 0.335 mm, and kernel Y-Detail/Smooth. The CT data were analyzed by evaluating both individual cross-sections and the entire diaphysis to quantify structural and biomechanical properties. Diaphyseal plasticity in long bones reflects remodeling throughout life in response to mechanical loading [21], and cross-sectional geometry (CSG) provides insight into biomechanical performance. Key CSG parameters used in femoral biomechanical analysis include the polar moment of area (J), which indicates mean torsional and bending stiffness, and the second moments of area (Ix and Iy), which specify bending stiffness along particular axes. Relative cortical area (%CA) measures differential developmental and aging processes. The second moments of area (I) assess bending rigidity in a given plane, while the polar second moment of area (J) reflects resistance to torsion and overall rigidity. Measurements and CSG parameters in this study include anteroposterior (AP) and mediolateral (ML) widths (Supplementary Table S14), total subperiosteal cross-sectional area (TA, mm^2^), cortical area (CA, mm^2^), percent cortical area [%CA = (CA/TA) × 100], second moments of area [Ix (ML axis) and Iy (AP axis), mm^4^], minimum and maximum second moments of area (Imin and Imax, mm^4^), and the polar moment of area (J = Ix + Iy; see Supplementary Tables S15–S19). Although the femur is incomplete and does not allow for the direct estimation of torsion, torsional stiffness was assessed by analyzing Ix and Iy (Imax and Imin), as the torsional stiffness of a tubular bone depends on its polar moment of inertia. %CA was also evaluated, reflecting the size of the medullary cavity and responses to mechanical loading, such as the history of endosteal versus periosteal deposition and resorption, and age-related changes [22]. To analyze patterns of change in femoral diaphyseal transverse properties among Pleistocene “*Homo*”, the cross-sectional parameters TA, CA, Ix, Iy, Imax, Imin, and J, as well as the ratios %CA and Imax/Imin, were compared to other samples. These parameters were collected using MomentMacroJ v1.4B (available as freeware at https://fae.johnshopkins.edu/chris-ruff/; accessed on 11 February 2025) for ImageJ, on selected cross-sections at 20, 35, 50, 65, and 80% of the total femur length (Supplementary Tables S15–S19). Because the C7 femur has incomplete epiphyses, total length was approximated by comparison with scaled reference samples (Supplementary Figure S3). The 2D images were derived from the 3D model using Amira 6.

The metric data of the teeth, specifically the bucco-lingual (BL) and mesio-distal (MD) crown dimensions (Supplementary Table S10), were recorded following the protocol of Hillson et al. [23]. The morphological features of the teeth were assessed using the ASUDAS method of the Arizona State University Dental Anthropology System [24]. Micro-Computed Tomography images of the two lower molars (C11, LRM3 and C12, LLM3) were acquired using a ZEISS Xradia Versa 610 X-ray microscopy system (Carl Zeiss, Oberkochen, Germany) at the Research Center on Nanotechnologies Applied to Engineering (CNIS), Sapienza University of Rome (Italy). This instrument provides sub-micron-scale resolution images with enhanced contrast by combining geometric magnification and optical magnification with a high-flux X-ray source, thus overcoming the limitations of traditional X-ray Computed Tomography (CT). The Guattari Cave mandibles (C2 and C3) were scanned in November 2017 at the Core Facility for µCT Micro-Computed Tomography at the University of Vienna using a custom-built VISCOM X8060 (Germany) µCT scanner. Scan parameters were slightly adjusted for each specimen: 140 kV, 160–180 µA, 2200–2500 msec, diamond high-performance transmission target, 0.75 mm copper filter, and isometric voxel sizes between 10.6 and 12.7 µm. The µCT images were acquired from 1440 different angles. Using filtered back-projection in VISCOM XVR-CT 1.07 software, these data were reconstructed as 3D volumes with a color depth of 16,384 gray values and a resolution of 20 µm. The resulting slices display differences in X-ray attenuation, which are primarily due to differences in the density of the object studied [25]. Three-dimensional reconstruction and segmentation analysis were carried out using Visage Amira 6.3 software (Thermo Fischer Scientific, Waltham, MA, USA) and using the latest available versione ov Bone J (1.4.3.0.) plugin for ImageJ 1.54 (NIH) [26]. The segmentation procedure was based on a semi-automatic threshold approach using the half-maximum height method (HMH) [27]. The 3D structures of the enamel, dentine, and coronal pulp cavity were obtained using the region of thresholding protocol (ROI-Tb) [28], which involves repeated measurements across different slices [29]. Virtual sections of each tooth were then extracted. The cementum–enamel junction (CEJ) was defined as the cervical line and determined manually, while the base of the pulp cavity chamber was established at the point where the interradicular canals begin. Variation in internal morphology and relative proportions of dental tissues were determined through digital analysis of linear, volumetric, and surface variables. We used the following abbreviations to identify the dental tissue volumes (mm^3^) [Supplementary Table S11]: Ve (enamel); Vcdp (coronal dentine and pulp chamber); Vpc, coronal pulp chamber volume; Vc (total crown = enamel, dentine, and pulp); Vcervix root cervix (pulp chamber ¼ situated inside the crown and root cervix); Vbranch root branch and Vt (total tooth = enamel, cementum, dentine, and pulp). In addition to the volumetric measurements, the linear and surface variables were identified SEDJ (EDJ surface, mm^2^).

Volumetric proportions of dental tissues are defined as:

- Vcdp/Vc% of coronal volume that is dentine and pulp (=100*Vcdp/Vc).
- Three-dimensional AET [3D average in mm of enamel thickness (=Ve/SEDJ)].
- Three-dimensional RET [scale-free, relative 3D thickness of enamel (=100*3D AET/Vcdp1/3) [30].
- VBI volumetric bifurcation (Supplementary Figure S4) index in % (=Vcervix/[Vcervix + Vbranch] × 100) [31]. Corresponding with the classification scheme of Keene [32] a value of 0–24.9% denotes a cynotaurodont molar, a value of 25–49.9% a hypotaurodont molar, a value 50–74.9% a mesotaurodont molar, and a value of 75–100% a hypertaurodont molar (Supplementary Table S12).
  To obtain the greatest amount of information on the sample, we evaluated the thickness of the lateral enamel excluding the occlusal one. In Amira (6.3.0, FEI Inc., Agawam MA, USA), we defined the plane of the occlusal basin, a plane parallel to the cervical plane and tangent to the lowest point of the occlusal basin enamel. Subsequently, all the material above the plane of the occlusal basin was removed and only the enamel, dentin, and pulp between these two planes were measured [33]. In the text and figures, we use the following abbreviations to identify the dental tissue volumes (mm^3^):
  ○ LVe (lateral enamel volume).
  ○ LVcdp (lateral volume of coronal dentin including pulp enclosed in the crown).
  ○ LVpc (lateral coronal pulp chamber volume).
  ○ LVc (total lateral volume of the crown, including lateral enamel, dentin, and pulp).
  ○ LSEDJ, in mm^2^ (lateral surface of the EDJ).
  ○ (LVcdp/LVc = 100*LVcdp/LVc in%). Percentage of dentin and pulp in the volume of the lateral crown
  ○ Three-dimensional average enamel thickness (3D LAET = LVe/LSEDJ in mm).
  ○ Three-dimensional relative lateral enamel thickness [3D LRET = 100*3D LAET/(LVcdp1/3)], a measurement without scale.

The accuracy of the microtomographic-based measurements was tested for intra- and inter-observer error using three different individuals; differences were <3%.

Internal anatomy was analyzed using bucco-lingual (b/L) sections across the two dentine and pulpal horn tips of the mesial cusps of the crown, as well as along the enamel junction. This approach allowed us to obtain both perpendicular and horizontal views of each root canal. To visualize the topographical distribution of enamel thickness, particularly in the C2 and C3 samples where wear was present, we imported the meshes obtained from CT scan segmentations into MeshLab. In MeshLab, we applied the Shape Diameter Function (SDF) filter, which estimates the local diameter of the object at each point on the mesh surface by sending several rays inside a cone centered around the point’s inward-normal. This process provides a measure of the neighborhood diameter at each point [34]. We then used the Colorize by Vertex Quality filter to generate a topographic map of thickness, with increasing thickness represented by a chromatic scale from dark blue to red. To establish the phyletic relationships within this sample, we conducted both qualitative analyses, exploring morphological variations and affinities, and quantitative multivariate analyses. The latter involved comparing our data with synchronic and diachronic datasets available for African, European, and Asian *Homo* (with particular attention to the south-eastern area), as well as modern humans. For the complete sample, we performed a multivariate non-parametric analysis using principal components analysis (PCA) on 23 raw variables. PCA was calculated on the correlation matrix to standardize variation, and all statistical analyses were performed using SPSS 20.0. In order to maximize the number of samples for inclusion in PCA, we used the following variables: maximum cranial length (g-op, M1), *maximum* cranial breadth (eu-eu, M8), minimum frontal breadth (ft-ft, M9), *maximum* frontal breadth (co-co, M10), *supraorbital torus* breadth, height (Po-Br, M20), bi-frontomalare temporale breadth (fmt-fmt, M43), postorbital breadth, *biasterion* breadth (ast-ast, M12), lambda-*inion* (M31), lambda-*asterion* (M30,3), *inion*-*asterion* and thickness at *lambda*, *inion*, internal occipital protuberance, *asterion*, bregma, *supraorbital torus* thickness at midorbit (SOTTM), *torus* highest point, parietal to bregma, parietal to lambda, parietal eminence, and parietal to the *asterion*. Finally, we carried out an overall analysis using both coefficients of variation and description of non-metric traits in a data-combined approach within Middle and Late Pleistocene Eurasian groups.

Dummy variables, defined as traits that are always present or always absent among all cases, were excluded from the analysis [35]. The distance between two samples (dij) was calculated by dividing the number of variables present in one individual and absent in the other (scored as 10 or 01) by the total number of variables, including those present or absent in both individuals (scored as 11 or 00) [36]. The binary scores and formulas were compiled in a spreadsheet to generate a matrix, which was then used for Cluster Analysis employing the WPGMA method and squared Euclidean interval. Paleogenomic analyses of the entire dental sample and petrous bone were conducted in Leipzig and Vienna. However, due to strong diagenesis resulting in a scarcity of collagen, it was not possible to extract aDNA. In the absence of paleogenomic results, phenotypic variation was assessed by combining mixed, metric, and non-metric traits, together with an analysis of available genetic data in the literature. For the interpretation of possible gene flows and migratory scenarios, we relied on published data, particularly the results of Posth et al. [37] and Hajdinjak et al. [38]. Where available, genetic information on the percentage of Neanderthal ancestry has been discussed in the following Section 5.2. The morphological traits analyzed in this study are considered derived features of Neanderthals or *H. erectus* and were used for taxonomic identification.

## 4. Results

### 4.1. Descriptive Morphology

***Cranium***

**Circeo 4 (C4**)

**Figure 10:**
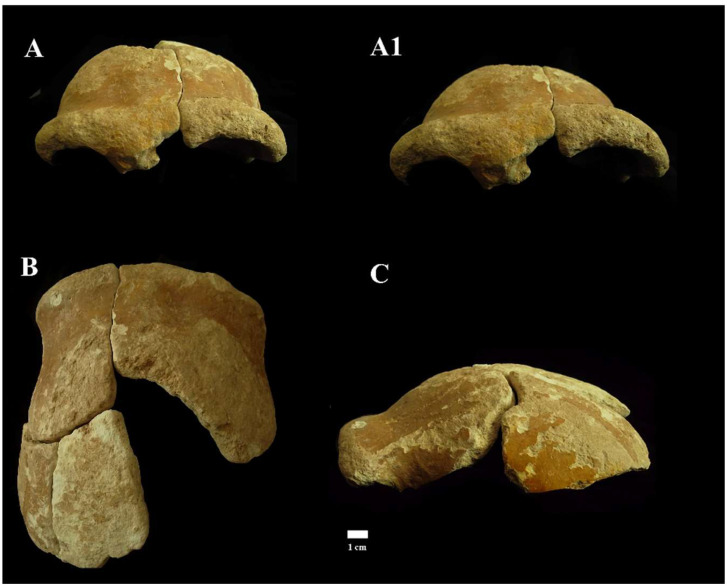
Circeo 4, frontal bone. View: Frontal, anterior–superior (**A**) and anterior (**A1**); superior (**B**); and left lateral (**C**).

Cranial remains found inside the cave, “Antro del Laghetto” area (Figure 4, localization) [Figure 10 and Supplementary Figure S5A].

A portion of *calvarium* is composed of four elements constituting a large part of the frontal bone and a small part of the left parietal bone. The frontal bone consists of a right and a left *squama frontalis*. There is mild asymmetric retro-orbital narrowing. The *supraorbital torus* is thick, and the prominent glabella is in line with the nasion. The orbits are rounded (Supplementary Catalog of Specimens).

Despite the strong extracranial cortical erosion, a short section of coronal suture on the left frontal *squama* remains preserved (Supplementary Figure S6), a relevant *data* not only from a metric point of view but also from a diagnostic point of view as it allows us to formulate an approximate age diagnosis. The individual, therefore, according to modern parameters [20], could be an adult, but not too young, considering the persistence of sutural denticles.

**Circeo 5 (C5)**

**Figure 11:**
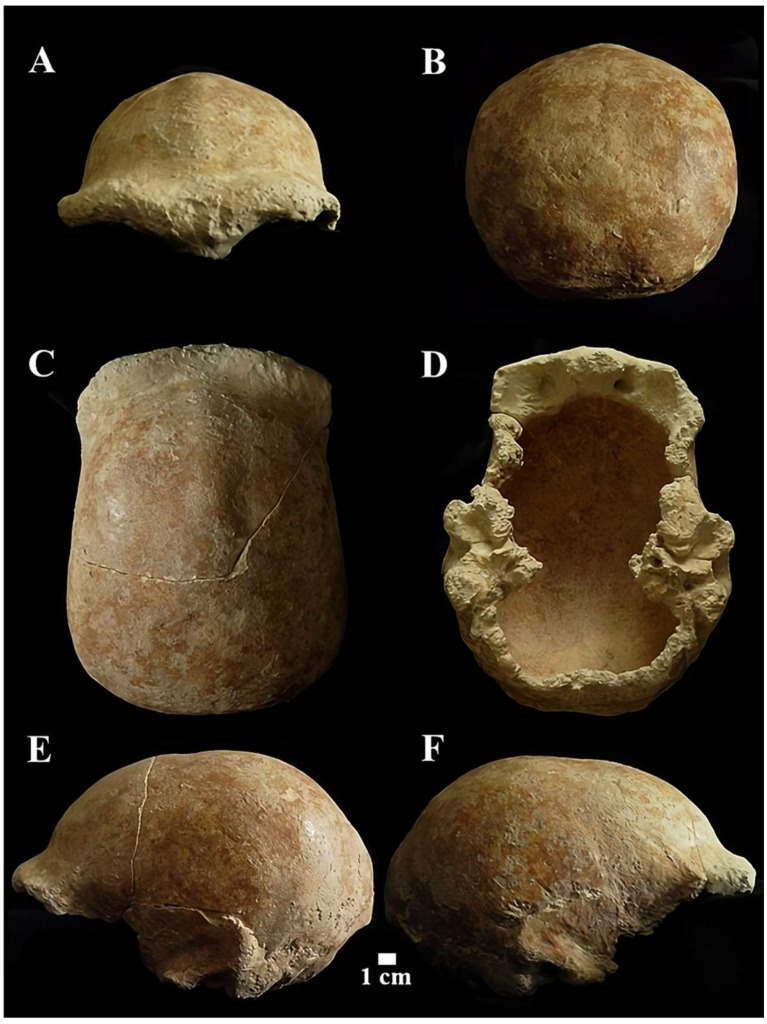
Circeo 5 *calvarium*. View: **A**, frontal anterior; **B**, posterior (occipital); **C**, superior; **D**, inferior (*basicranium*); **E**, right lateral; **F**, Left lateral.

*Calvarium* found inside the cave, “Antro del Laghetto” (Figure 4, localization) [Figure 11 and Supplementary Figure S5B,B1].

The *neurocranium* (*Calvarium*) is moderately elongated in the anteroposterior direction and semi-rounded in the occipital region. In posterior view, the parietals are quite vertical with a curvature starting in the upper third (Figure 11B). The lateral cranial walls are, therefore, parallel to the superior parietal bones which are short, flat, and slightly convergent. The lateral cranial walls are, therefore, parallel to the superior parietal bones which are short, flat and slightly convergent. The orbits are rounded (Supplementary Catalog of Specimens). The morphological traits and the synostosis of the cranial sutures are consistent with those of an adult female individual.

**Figure 12:**
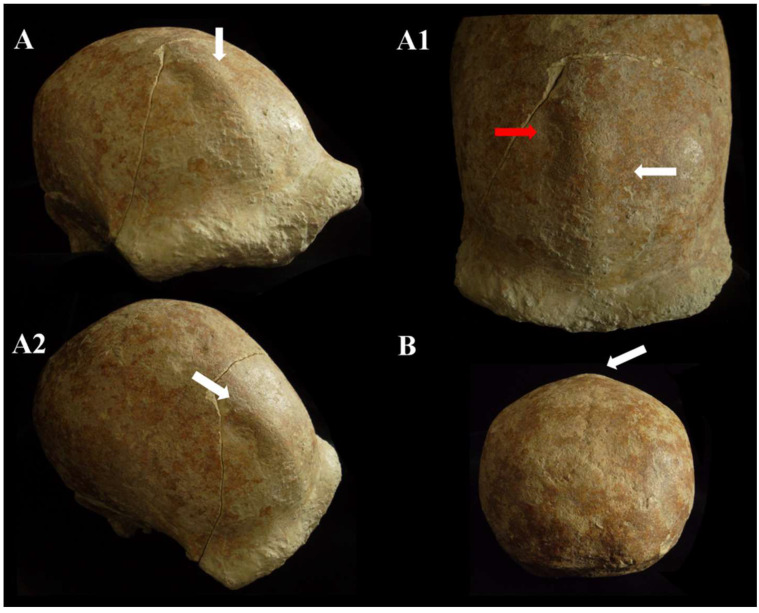
Circeo 5, frontal *keel*. View: superior–anterior (**A**,**A1**), superior–lateral (**A2**), and posterior (**B**). The white arrows indicate the frontal *keel*. The frontal *keel* is defined by a pair of anteroposteriorly long and mediolaterally wide depressions in the frontal squama (the red arrow in **A1** indicates an accentuation of depression). Not to scale.

The *squama frontalis* has a distinct post-toral plane and a high, rounded frontal. The *supratoral sulcus* is continuous and the *supraorbital torus* is thick. The glabella is in line with the nasion. There is a slight *supraorbital* narrowing and residual traces of the lower part of the metopic suture. It has a frontal *keel* that extends along much of the sagittal line. It is defined by a pair of long, mediolaterally wide anteroposterior depressions in the frontal “squama” [39,40] (Figure 12). It presents a well-developed bregmatic eminence, associated with two pre-bregmatic parasagittal depressions that do not involve the parietal bone (Figure 12). The bregmatic eminence is not separated from the frontal *keel*. The small temporal bone (*squama temporalis*) shows a linear upper profile (Supplementary Catalog of Specimens). The juxtamastoid eminence is incomplete and eroded, and both the juxtamastoid and occipitomastoid crests appear to be absent. The mastoid processes are small with underdeveloped apophysis and incomplete apex.

*Basicranium* (Comprehensive Supplementary Catalog of Specimens).

**Figure 13:**
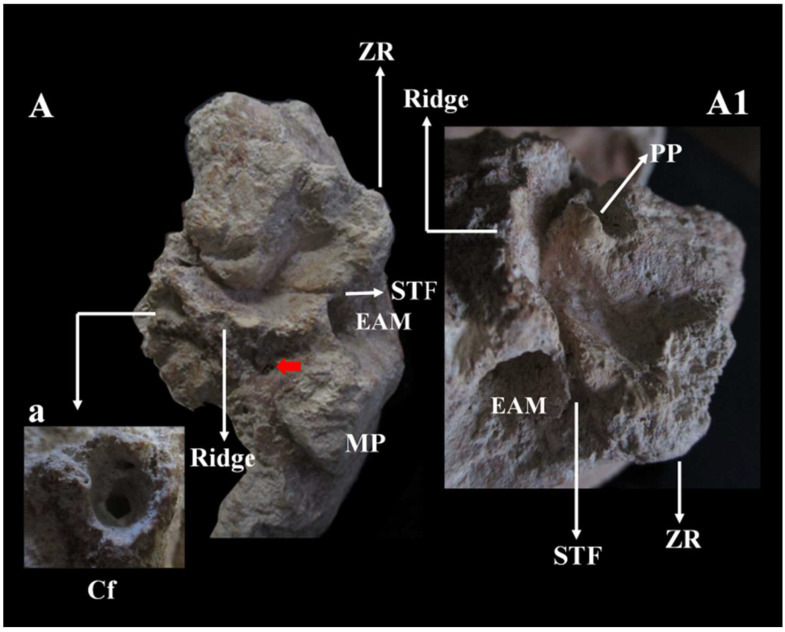
Circeo 5, *basicranium*. Temporal bone left mandibular fossa (TMJ) viewed in *norma basalis*. **A**, styloid *foramen* (red arrow). **Ridge** (process *supratubalis*?), wrinkled crest that extends to the External *Acoustic Meatus* (**EAM**). Absence of the vaginal process and the styloid process. Squamotympanic fissure (**STF**). **a**, Detail of the Carotid *foramen* (**Cf**) with bony thickening of the marginal rim of the *foramen* and narrowing of the carotid canal. **A1** detail of **A**. Note the absence of the postglenoid process, the position of the squamotympanic fissure (**STF**) that runs into the roof of the *fossa* itself, and the coronally oriented tympanic plate. **MP**, Mastoid Process; **ZR**, Zygomatic Root; **PP**, Preglenoid (entoglenoid) Process (?). Not to scale.

In the *basicranium* (Figure 13 and Supplementary Figure S7), the mandibular *fossa*, bilaterally, is preserved and deep. It has a squamotympanic fissure (STF) that runs into the roof of the *fossa* itself and a coronally oriented tympanic plate [41]. The styloid process is not fused to the skull base, and the vaginal process is absent. The carotid *foramen*, visible only on the right, is posterior to the STF and shows a thickening of the opening margin and a narrowing of the channel (Figure 13a). The styloid process is not fused to the skull base, and the vaginal process is absent. The *postglenoid* process is absent. The *sigmoid sinuses* are vertical, and near the left *sigmoid sinus*, there is a distinct *sulcus* (Supplementary Figure S8).

The occipital bone shows ectocranial thickening on the superior nuchal line (underdeveloped nuchal *torus*). The transverse *torus occipitalis* expands laterally in the direction of the *asterion*, most noticeably on the right side. Between the superior and supreme nuchal line, there is a small and smooth *suprainiac fossa*, circular in shape. The *inion* is located well above the *endinion*. The posterior projection of the *squama occipitalis* (*chignon*) is slight. Sutural synostosis is characterized by interdigitations on the outer table and incomplete obliteration on the inner table.

Overall, individual C5 is a young adult female [20] who presents with mild internal frontal hyperostosis.

**Circeo 6 (C6)**

**Figure 14:**
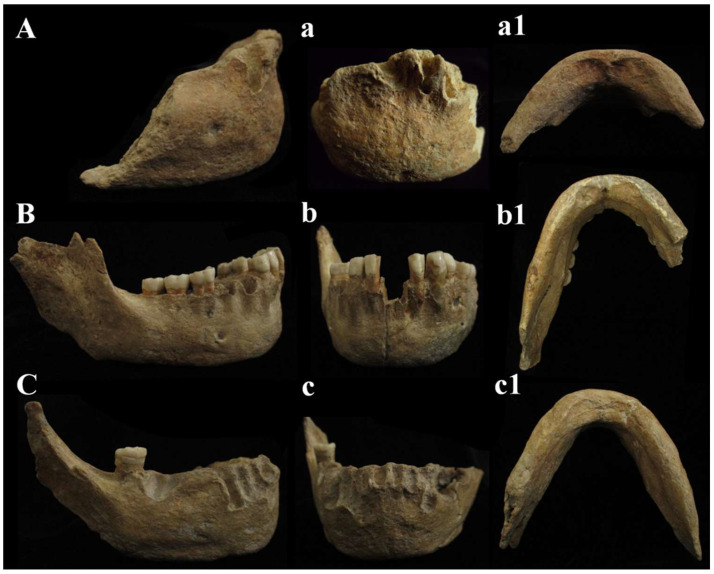
Circeo mandibles, comparison. Circeo 6 (**A**), mandibular symphysis recent discovery, **Circeo 2** (**C**) and **Circeo 3** (**B**), original specimens, respectively, from 1939 and 1950. **Circeo 6**, view: (**A**) lateral (right); (**a**) anterior; (**a1**) inferior. **Circeo 3**, view: (**B**) lateral (right); (**b**) anterior; (**b1**) inferior. **Circeo 2**, view: (**C**) lateral (right); (**c**) anterior; (**c1**) inferior. Not to scale.

Mandible found inside the cave, “Antro del Laghetto” area (Figure 4 and Figure 6, localization) [Figure 14A,a,a1].

C6 is an anterior interforaminal portion (synphysis). The mandibular symphysis is characterized by the absence of mental *trigone*. It shows a slight hint of the chin eminence (*mentum*) with *incurvatio mandibulae* in lateral view, which may be accentuated by the presence of bone atrophy due to *intra-vitam* tooth loss. The digastric *fossa* is directed downwards and posteriorly. C6 has on the right side (the left side is incomplete) a single oval mandibular *foramen*, horizontal and parallel to the masticatory plane and located under M_1_ (P_4_) [Supplementary Catalog of Specimens].

In C6, severely atrophied alveolar areas show residual alveolar ridge morphology consistent with tooth loss before death (Supplementary Table S13). The alveolar ridge of the central incisors does not present horizontal atrophy [42,43], and the bone level is normal [44]. The presence of a thin fracture line involving only the external alveolar bone is noted in correspondence with the right central and lateral incisors [Supplementary Catalog of Specimens].

**Circeo 8 (C8)**

Occipital bone (*squama occipitalis*) found inside the cave, “Antro del Laghetto” area, composed of a portion plus one fragment (Figure 4, localization) [Supplementary Figure S9; Supplementary Catalog of Specimens].

The occipital plane is convex. The nuchal plane is incomplete. An occipital bun is absent. The “nuchal *torus*”, heavily eroded, shows ectocranial thickening located above the superior nuchal line. The *suprainic fossa* is elliptical and is located on the upper margin of this thickening. Its surface is pocked [45]. The *inion* is located immediately above the *endinion*. The *torus* has no margins but upper and lower thinning. The transverse torus is thickened medially and lacks significant lateral development. External occipital protuberance is absent. The interdigitations of the lambdoid suture, without traces of synostosis [20], and the pocked surface of the suprainiac fossa indicate that this occipital bone could belong to a young individual and is therefore not compatible with C4.

**Circeo 9 (C9)**

Palatine process of maxilla found inside the cave, “Antro del Laghetto” area (Figure 4, localization) [Supplementary Figure S10].

Although the finding shows severe erosive aggression, the presence of a progressive alveolar ridge atrophy due to tooth absence is evident. The *sinuses* sit above the upper teeth. The maxilla in the palatal view (palatine) presents slight traces of *palatine torus* along the margins of the medial palatine suture. There is asymmetrical thickness between the right and left sides of the palate. Residual dental alveoli are present on the right while the left anterior alveolar ridge shows bone remodeling. The nasal view shows a large piriform opening. Based on the features of the internal nasal region presented by Schwartz et al. [46], we provide a description, in Supplementary Catalog of Specimens, of the topographic relief of the nasal cavity wall of C9, which preserves part of the nasal *fossae* with the spinal crests and nasal ridges (Supplementary Figure S10).

**Dental sample**

**Circeo 10a and b (C10a-C10b)** found in the area outside the cave (Figure 2, localization).

Permanent maxillary molars and alveolar part (Supplementary Figure S11).

Second and third right upper molars (M^2^ and M^3^), contiguous and compatible. Both are probably from the same adult individual. A small portion of alveolar bone is present (M^2^ in alveolar bone). The estimated age for tooth wear is adult. Dental traits: M^2^ right: presence of grade 3.5 *metaconus* and hypocone, absence of 5th cusp, Carabelli tubercle and parastyle, and presence of grade 2 enamel extension and three roots; M3 right: absence of 5th cusp.

**Circeo 11 (C11)** found inside the cave, “Antro del Laghetto” area (Figure 4, localization).

Permanent lower molar (Supplementary Figure S12).

**Figure 15:**
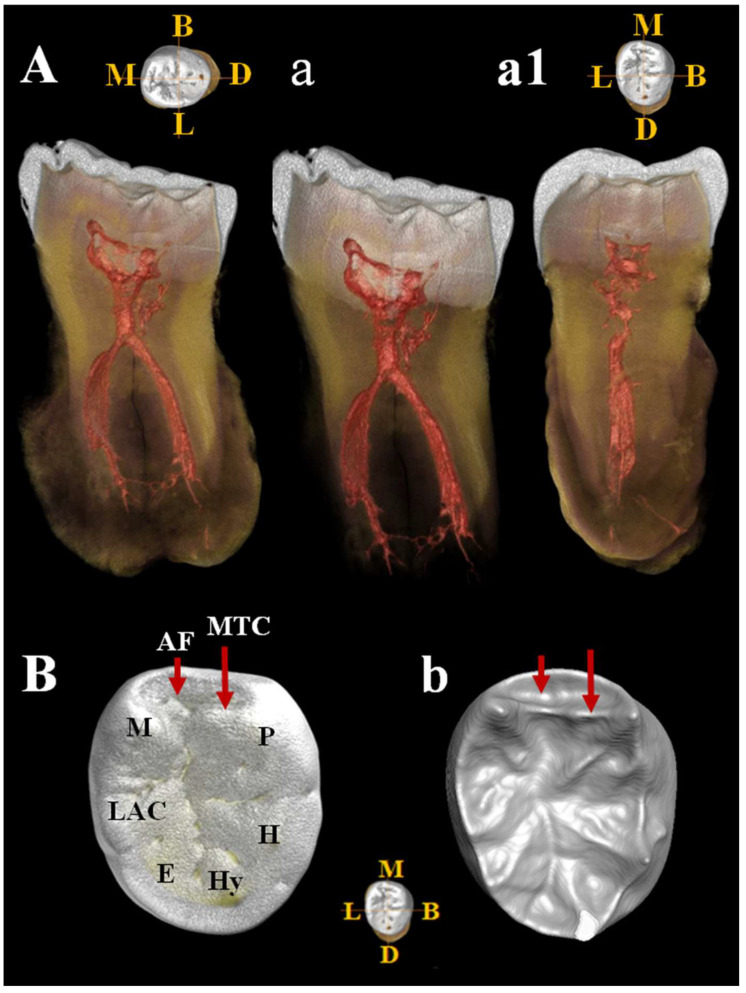
µCT images of Circeo 11 lower right third molar (LRM3) with club-shaped *hypercementosis*. Dentinal structures of the root: **A**, view of the mesio-distal section with *hypercementosis*; **a**, view of the mesio-distal section without *hypercementosis*, digitally removed; **a1**, view of the bucco-lingual section. Note the presence of a connection between the two main canals in the apical third and the presence numerous lateral accessory canals and apical deltas. The apical *foramina* have a regular direction. **b**, Occlusal view of the crown surface (enamel) illustrating the main cusps and one probable accessory cusp; **B**, occlusal view of the enamel–dentin junction (EDJ) [enamel digitally removed], showing the presence of the mid-trigonid crest (**MTC**) and the anterior fovea (**AF**). Abbreviations: **B**, Buccal; **L**, Lingual; **M**, Mesial; **D**, Distal; **P**, Protoconid; **H**, Hypoconid; **Hy**, Hypoconulid; **E**, Entoconid; **LAC**, Lingual Accessory Cusp; **M**, Metaconid.

C11 is a probable lower right third molar (M_3_) because the contact facet on the distal side is missing. Estimated age for tooth wear: young adult. Dental traits: presence of mid-trigonid ridge (grade 1A) and fovea anterior, *sulci* pattern = +, presence of 5 cusps, absence of 6th cusp and presence of a probable lingual accessory cusp (Figure 15B,b). C11 shows a club-shaped diffuse apposition of *cementum* that cover a large part of the tooth root, giving it a “bulbous” appearance (*hypercementosis*). It involves the apical and middle root third and root furcation areas. The roots are fused but retain a thin line of demarcation. µCT images (Figure 15) show a thickened root highlighted by a radiopaque area consistent with the presence of *hypercementosis* (Figure 15A–a1). A thin and partial separation between the roots is visible, associated with the presence of numerous lateral and accessory canals, as well as apical deltas (Figure 15A–a1). There is a connection between the two main canals in the apical third (Figure 15a) and the respective apical *foramina* with regular direction are present, documenting a probable tooth vitality. A loss of contour of the tooth roots is visible due to fusion with the calcified mass, a morphology that could mimic a cementoblastoma. Moderate taurodontism is present with an index corresponding to mesotaurodontism (Supplementary Table S12).

**Circeo 12 (C12)** found inside the cave, “Antro del Laghetto” area (Figure 4, localization).

Permanent lower molar (Supplementary Figure S12).

**Figure 16:**
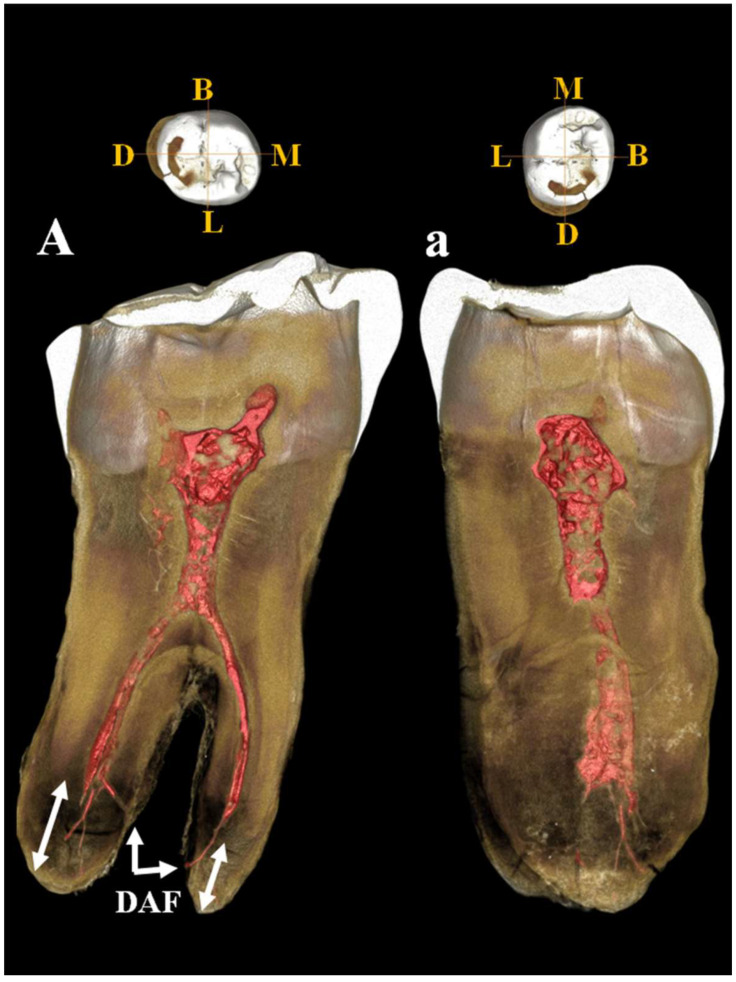
µCT images of Circeo 12 lower left third molar (LLM3). Dentinal structures of the root: **A**, view of the mesio-distal section; **a**, view of the bucco-lingual section. Mesio-distal section shows the periapical apices of both roots with *cementum* apposition (double white arrows), deviation of the canal and of apical *foramen* in the mesial root, and presence of an accessory lateral canal in the distal root. Note the presence of residual apical deltas in both roots. Abbreviations: **DAF**, deviated apical *foramen*; **B**, Buccal (vestibular); **L**, Lingual; **M**, Mesial; **D**, Distal.

Left lower third molar (_3_M). Estimated age for tooth wear: adult. Dental traits: sulci model = (+), presence of n. 4 cusps, presence of mid-trigonid ridge (grade 1A) and fovea anterior, absence of *protostilis*, 5th, 6th, 7th cusps and extension of enamel, presence of two roots. The µCT image (Figure 16) shows the periapical apices of both roots with a slight apposition of *cementum* that has caused a deviation of the canal and apical *foramen* in the mesial root and an apical rounding with the formation of an accessory lateral canal in the distal root (Figure 16A,a). The presence of residual apical deltas is noted in both roots. As in C11, moderate taurodontism is present with an index corresponding to mesotaurodontism (Supplementary Table S12).

**Circeo 13 (C13)** found inside the cave, “Antro del Laghetto” area (Figure 4, localization). Permanent upper canine (Supplementary Figure S11).

Right upper canine (C^1^). Estimated age for tooth wear: young adult. Dental traits: probable absence of shovel absence of tubercle in the cingle, extension of enamel, presence of root. Slight apical *hypercementosis*.

**Circeo 14 (C14)** found inside the cave, “Antro del Laghetto” area (Figure 4, localization) [Supplementary Figure S11].

Permanent upper second premolar tooth (Supplementary Figure S11).

**Figure 17:**
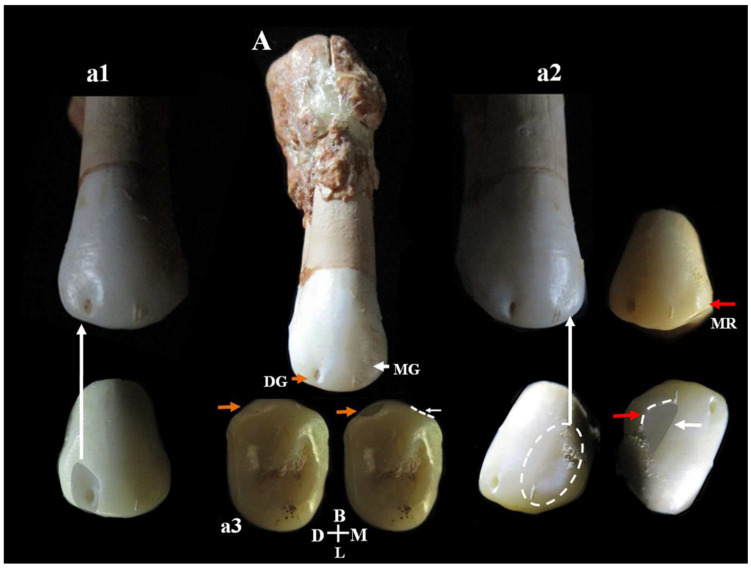
Mesio-distal buccal vertical groove of the second upper premolar, Circeo 14. **A**, Complete vestibular side of Circeo 14. The orange arrow highlights the distal buccal vertical groove on the crown surface (detail in **a1**), and the white arrow the mesial groove (detail in **a2**). **a3** is the occlusal view of both grooves. Note the severe *hypercementosis* involving the apical third of the root. The buccal groove, an archaic character from the Middle and Upper Pleistocene, is a shallow vertical groove accompanying a ridge on the mesial and distal margins of the vestibular surface. In our sample, a distal groove associated with a clear concavity was observed while on the mesial side, there is a weak and indistinct concavity associated with a small vertical ridge (**MR**, red arrow). **DG**, Distal Groove; **MG**, Mesial Groove; **MR**, Mesial Ridge; **D**, Distal; **M**, Mesial; **B**, Buccal (vestibular); **L**, Lingual. Not to scale.

C14 is a right upper second premolar (P^4^) showing a club-shaped diffuse apposition of cementum that covers a large part of the tooth root (*hypercementosis*). Estimated age for tooth wear: young adult. Dental traits: presence of two lingual cusps, enamel extension. On the buccal surface of the crown, there is a *plesiomorphic* character from the Middle and Upper Pleistocene consisting of a buccal vertical groove. The vertical groove is present on the distal aspect of the vestibular surface and is associated with a clear concavity. A weak and indistinct concavity is also present on the mesial aspect associated with a small vertical ridge (Figure 17). The buccal vertical groove is visible on 3D reconstruction of the outer-enamel surface (vestibular) and on the enamel–dentine junction (Supplementary Figure S13).

**Circeo 15 (C15)** found inside the cave, “Antro del Laghetto” area (Figure 4, localization) [Supplementary Figure S14].

Crown and root part of a permanent tooth (Supplementary Figure S14).

Part of the crown and root of a small permanent upper right first molar (M^1^). The crown, although incomplete, shows a mesiodistally compressed morphology and the roots, which are also incomplete, are small and strongly splayed. Distal contact facet is present. Severe wear. Dental traits: grade (1) enamel extension.

**Postcranial skeleton**

**Circeo 7 (C7)**

Right femur (diaphysis) found inside the cave, “Antro del Laghetto area” (Figure 4, localization) [Supplementary Figure S15].

C7 is without both epiphyses. Femoral shaft is curved, platymeric in the subtrochanteric part and with weak midshaft pilastric index. The *linea aspera* is continuous, modest, and with a pilaster consisting of a light underlying bony crest (Supplementary Figure S16). The cortical distribution pattern of C7 for the 80%, 65%, and 50% cross-sections shows a constant *maximum* cortical thickness on the medial side and is variable from lateral to lateroposterior on the lateral side (Supplementary Figure S3). Supplementary Tables S4 to S8 show the diaphyseal cross-sectional properties of the proximal, distal, and mid-diaphyseal sections of C7 and the comparative specimens.

**Circeo 16 a (C16a)**

Incomplete left coxal bone found inside the cave, “Antro del Laghetto” area (Figure 4, localization) [Supplementary Figure S17].

The partial iliac bone preserves the incomplete iliac wing, a small portion of the auricular surface, a remnant of the tubercle of the iliac crest, a small, preserved margin of the anterior superior iliac spine and the anterior inferior iliac spine. Finally, a portion of the *acetabulum* is preserved (*fossa*), with exposure of the spongy bone on the acetabular rim, and a portion of the greater sciatic notch. The *ischium* and *pubis* are completely missing. In the lateral view, the presence of a single ventral iliac buttress acetabulocrystal (vertical) is noted. The thickness of the buttress is variable. In medial view, there is a well-defined arcuate line and a thick iliosciatic buttress (Comprehensive Supplementary Catalog of Specimens). The composite morphology of the arch, formed by the sciatic notch and the auricular surface, is consistent with the female morphology.

**Circeo 16 b (C16b)**

Incomplete right coxal bone found inside the cave, “Antro del Laghetto” area (Figure 4, localization) [Supplementary Figure S18].

The partial iliac bone preserves the incomplete iliac wing, a portion of the auricular surface, a small remnant of the iliac crest (tubercle iliac crest?), a small preserved margin of the anterior inferior iliac spine, the latter accentuated by an evident supra-acetabular sulcus which extends between the acetabulospinal buttress and the acetabular rim, a portion of the acetabular fossa with exposure of the spongy bone on the acetabular rim, and finally a portion of the greater sciatic notch. The ischium and pubis are completely missing. In the lateral view, a single ventral iliac acetabulospinal buttress is present (concretions are present). The thickness of the buttress is variable. There is a thick iliosciatic buttress (Comprehensive Supplementary Catalog of Specimens). The composite morphology of the arch, formed by the sciatic notch and the auricular surface, is consistent with the female morphology.

### 4.2. Comparative Morphology and Discussion of Morphometric Data

The first variant emerges from the direct comparison between the remains of 1939–1950 (C1, C2, and C3), classic Neanderthals, and the recent remains (C4, C5, C8, and C6), which are characterized by a mixture of *autapomorphic* and *plesiomorphic* traits shared with the monophyletic group (Proto-Neanderthal and classical European Neanderthal, *Erectus s.s*. and *s.l.*, and *Sapiens*). A similar pattern of variability is observed in the dental sample.

***Frontal bone C4 and C5***. Both present a similar morphology and a notable thickness (Supplementary Figure S19). The morphology and total frontal bone thickness (measured as the combined thickness of the diploe and the inner and outer tables) differ from those observed in C1. This difference may reflect variability within the species rather than differences at the *genus* level. In anterior view, the supraorbital torus of C4 and C5 exhibits a morphology similar to that seen in Asian *H. erectus* (e.g., Zhoukoudian 12 and 5), Late European *Erectus* such as Lazaret 24 (170 ka), and European Proto-Neanderthals like Biache-Saint-Vaast 2 (BSV2) and Petralona. The *supraorbital torus* is wide and continuous, and when viewed from above (*norma verticalis*), there is no glabellar depression present. The *supratoral sulcus* is defined and continuous in C5 but discontinuous in C4, where it is interrupted by a convexity in correspondence with the glabella as in the Late Indonesian *Erectus* Ngadong 5 [47]. In C4, this convexity may be caused or accentuated by the morphology of the frontal *sinus*. The frontal *sinuses* of C4 are asymmetric, well developed, and contain multiple chambers (Comprehensive Supplementary Catalog of Specimens). The minimum frontal diameter of C4 and C5 is greater than that of BSV2 and Neanderthals, but is very similar to C1, slightly lower than Amud 1, and comparable to Ngadong 11 and WLH 50 (Australian “*H. sapiens*”) [Supplementary Table S3]. C4 and C5 show a postorbital narrowing and a widening towards the parietal walls, but this widening is less pronounced than in classical Neanderthals. The sagittal profile of the frontal bone in C4 and C5 differs from that of classic Neanderthals such as C1, La Chapelle-aux-Saints, and La Ferrassie 1, which display a more anteriorly positioned glabella relative to the *nasion*. In lateral view (*norma lateralis*), the upper profile of C5 is high and rounded, similar to the Indonesian fossils (Sangiran [S], Sambungmacan [SM], and Ngandong). It displays a convex frontal contour, which is absent in C4, and this convexity becomes more pronounced at the bregmatic eminence. The distance from the glabella to the bregma along the mid-sagittal plane is greater in C4 than in C5, but both measurements fall within the range observed in Javanese specimens (Supplementary Table S3).

***C5 calvarium.*** It displays a combination of Proto-Neanderthal *plesiomorphic* traits (such as the absence of the anterior mastoid tubercle) and Neanderthal *autapomorphic* traits (such as the digastric *sulcus* closed anteriorly) [Supplementary Table S2]. Some of these characteristics are also known in *H. erectus s.s.* Additionally, the morphology of the parietals—vertical with a curvature beginning in the upper third—is very similar to that of the archaic Middle Eastern *H. sapiens* specimen Manot 1 (Israel). Unlike the C1 skull, and presumably also C4, the frontal bone of C5 exhibits a frontal “*keel*” (Figure 12), a feature described by Schwartz et al. [40] as unique to Trinil 2 (an *autapomorphy*) and also present in Sangiran specimens. The frontal *keel* of C5, like that of Trinil 2, is characterized by a pair of anteroposteriorly long and mediolaterally wide depressions in the frontal squama [39,40] (Figure 12). C5, like the Indonesian specimens, also has a bregmatic eminence that does not extend bilaterally into the coronal *keels* or posteriorly into a sagittal *keel*, but it does possess a pair of small depressions located posterior to the bregma [39,40]. Contrary to the Ngandong specimens [48], the bregmatic eminence of C5 is not separated from the frontal *keel*. In all Proto-Neanderthal samples from Atapuerca Sima de los Huesos (SH), a mid-sagittal *keel* on the *squama frontalis* has been described [49]. However, the authors note that this feature does not exactly match the morphology and *autapomorphic* characteristics of the Asian *H. erectus* holotype Trinil 2, and they suggest that this trait is absent in Neanderthals [49]. The supraorbital *torus* of C5 appears more similar to that of Lazaret 24 [50] and BSV2 [48,51]. The latter has been classified as type III in the Cunningham classification system [51] and is considered similar to those of Sima de los Huesos 5, Bilzingsleben, and the Neanderthals [51]. The sagittal profile of the C5 *calvarium* also shows a similar contour to S2, SM1, and Ngandong.

In posterior view, the parieto-temporal curve in the coronal plane closely resembles the “tent” morphology of the Indonesian sample SM 4, a result of the presence of the bregmatic eminence (Figure 11B). However, the vertical lateral profile is very similar to that of the Western Asian (Israel) *H. sapiens* Manot1 (55 ka [52]) and differs from the rounded profile characteristic of classic Neanderthals such as C1, La Chapelle-aux-Saints, and Ferrassie1. In C5, as in Manot1 and modern humans, the *maximum* cranial width is positioned high, and the lateral walls are vertical and almost parallel [52]. The biasterionic breadth of C5 is larger than that of Manot 1, which is extremely small, but is similar to that of Shanidar 1, Sale (*Erectus*, Morocco), and Xuchang (XUC2) and falls within the range observed in the Java specimens (Supplementary Table S4). The convexity (bunning) of the occipital bone of C5 and C8 is less pronounced than in C1 (although it is more defined in C8 than in C5), Manot 1, Chapelle-aux-Saints, and Middle Pleistocene fossils from northern Africa (Jebel Irhoud, Morocco) and Europe (Neanderthals). According to Hershkovitz et al. [52], the presence of bunning is not necessarily related to interbreeding between Neanderthals and modern humans, as it is not present in Middle Eastern Neanderthals (e.g., Amud 1). Therefore, the authors suggest that this morphological trait originated in modern Near Eastern humans, or possibly even earlier in some African populations such as Aduma (~79–105 ka), who later migrated to the Levant. Furthermore, in C5, we observed a slight parietal flattening that extends into the lambdoid region. In Manot 1, as in BSV1, some features typically associated with classical Neanderthals, but occasionally present in other fossils, have been described. These include lambdoid flattening of the parietal bones associated with the occipital conformation, resulting in a double arched-shape profile with parietal and occipital concavities. This medial change in the posterior parietal curve, identified as a prelambdic depression, indicates the presence of an occipital bun, a trait described for C1, La Chapelle-aux-Saints, Spy 2, and La Ferrassie 1. In the post-obelic region of C5, there is a slight inflection followed by an occipital protuberance, which does not correspond to the classic bun. Therefore, we believe that in C5, the slight parieto-lambdoid flattening, in the absence of a classic Neanderthal occipital chignon, does not produce the double arch parietal and occipital profile described for Manot 1 and BSV1, but rather resembles a characteristic of the Ngadong specimens, as described by Zhang (Doctoral dissertation) [53], corresponding to a depression in the posterior parietal region that ends at the lambda. The occipital profile described by Schwartz [40] for S2 and SM1 as “rounded between the anteriorly inclined occipital and nuchal planes which gives the short occipital supero-inferiorly a blunt V-shaped profile”, shows similarities with C5, particularly when compared with SM1. In C5, Ngawi 1 and S3, the morphology of the occipital torus is similar to that of the Ngandong specimens, but less prominent [40].

***Temporal bone C5.*** Moreover, regarding the temporal bone, the presence/absence of a mosaic of very specific traits (*autapomorphic* and *plesiomorphic*) relating to *H. erectus s.s.*, *H. erectus s.l.* (Late Indonesian), and Neanderthal have been detected. *H. erectus* is usually described as having “well-developed or marked” mastoid and supramastoid crests, which are separated by a supramastoid groove or, in some cases, fused [53]. In C5, these crests appear to be present, separated by a slight supramastoid *sulcus*, and show a generalized hypertrophy of the temporal bone, but without the presence of an angular *torus*. The tympanomastoid fissure, which separates the tympanic plate from the mastoid process, is absent on the right but may be present on the left. On the left side, a slight line of separation between the tympanic plate and the mastoid process is visible, though it is interrupted by a small area of *post-mortem* damage. This trait is considered an autapomorphy of Asian *H. erectus* [54]. The mandibular *fossa* of C5 (Figure 13), which is bilaterally preserved (Supplementary Figure S7), exhibits a morphology very similar to that of the Ngandong specimens [41,55]. It features a coronally oriented tympanic plate (a *plesiomorphic* trait) [41] and the STF runs in the roof of the *fossa*, with the posterior wall of the temporomandibular joint (TMJ) formed exclusively by the tympanic plate. In C1, the tympanic plate is oriented coronally, but the STF is located posterior to the apex of the *fossa*. The tympanic plate is generally considered to be oriented coronally in *H. erectus* and sagittally in modern humans. The orientation in Neanderthals is debated: Weidenreich [56] and Stringer [54,57] argue for sagittal orientation, while other authors [58,59] suggest it is coronally orientated. In C5, the styloid process is absent (that is, it is not fused to the skull base) and the postglenoid process is also absent, as observed in the Ngandong specimen (Supplementary Table S2). The styloid process is fused to the *petrous* bone in all SH specimens and in Neanderthals as C1 (except some specimens from Krapina and Shanidar1; Supplementary Table S2) [41]. In Asian *H. erectus*, the *postglenoid* process is significantly reduced (apomorphy) [41] and the styloid process is absent (apomorphy) [41]. A well-developed *postglenoid* process is a feature of the Proto-Neanderthal SH sample, abd is present in BSV2 [51] and in classical Neanderthals (including C1 skull). The European Middle Pleistocene fossils, Castel di Guido and Ceprano, also show a well-developed *postglenoid* process [41]. In Middle Pleistocene Asian fossils, both conditions are present (Narmada, Dali, and Xujiyao show the styloid process although Hexian and Yunxian 2 lack it [41]). In C5, the digastric *sulcus* and the stylomastoid *foramen* are not aligned. Similarly in BSV2 and in classical Neanderthals, the digastric *sulcus*, the base of the styloid process and the styloid *foramen* are not aligned, as the styloid process is positioned more medially relative to the digastric *sulcus* [51].

In C5, the vaginal process of the *petrous* bone is absent (an *autapomorphy* of *H. erectus* [47]). However, there is a thickened and wrinkled crest (possibly the *supratubalis* process; see Figure 13) that extends, only on the left, up to the *meatus*, similar to what is observed in Sts19 [60], as well as in S2, S4, S17, Ngandong, and SM4 [61]. As in S4, S17, and Ngandong (6, 7, 10, 11, and 12), the carotid *foramen* in C5 is located posterior to the STF.

Similarly to the Ngandong and SM3 specimens, as described by Zhang (Doctoral dissertation, [53]), the mandibular *fossa* of C5 is deep. A short lateral part of the posterior margin of the mandibular *fossa* is open, due to the absence of the *postglenoid* process. The tympanic plate, mediolaterally short and coronally oriented, is higher than the articular eminence; its anterior surface is convex. The auditory *meatus* is oval, oriented almost vertically, and it is separated from the mastoid process.

In C5, the mastoid process is small, reflecting the retention of the *plesiomorphic* condition seen in *H. erectus* [47,56,57]. The anterior mastoid tubercle is absent, and the digastric *sulcus* is closed anteriorly (*plesiomorphy*) [Supplementary Table S2]. The anterior mastoid tubercle is also absent in early Proto-Neanderthals, such as the Atapuerca-SH [62] and BSV2 samples [51]. While the presence of an anterior mastoid tubercle is generally considered an *autapomorphic* trait of Neanderthals [48,54,59,63,64,65,66], Frayer [67] has reported several Neanderthal specimens lacking this feature including Gibraltar 1, La Quina 27, and Saccopastore 1 and 11 adult specimens of Krapina. The anteriorly closed digatric *sulcus* is a distinctive morphology consistently found in Neanderthals, although it may be present in several specimens from the Lower Zhoukoudian Cave [49]. This future is described as an elevation of the floor of the digastric *sulcus* in the anterior part, forming a saddle-like rise that nearly obliterates the *sulcus* [41,49]. A similar feature was also described by Guipert et al. [51] in the Proto-Neanderthal BSV2 specimen.

C5 *calvarium*, unlike C1 and similar to the Ngandong specimen, has a *squama temporalis* with a flat upper edge. In contrast, the apomorphic condition of a convex upper border of the *squama temporalis* is observed in Middle Pleistocene samples from Africa (Bodo, Salé), Europe (SH sample, Petralona and Steinheim), and Asia (Dali), as well as in Neanderthals and modern humans [41,49]. Furthermore, in C5, there may be traces of an atypical intracranial *sinus* drainage pattern (specifically, an arborizing *sigmoid sinus*), as described by Schwartz [40] in S2 and S4. This pattern consists of a distinct groove diverging from the *sigmoid sinus.* In C5, a distinct but small *sulcus* is visible near the left *sigmoid sinus* (Supplementary Figure S8), which could represent a remnant of the same system or a variant. In any case, this drainage model of the *sinus* is considered by the author to be potentially *apomorphic* for *H. erectus*, as seen in Trinil 2 [40].

***Occipital bone C5 and C8***. In C5, the *inion* is located well above the *endinion*, whereas in Manot 1, the *inion* is located below the *endinion*. Although the *endinion* region of C8 is heavily concretioned, it can still be determined that the *inion* is situated immediately above the *endinion*. The separation between *inion* and *endinion* is taxonomically important, as the *endinion* being located well below the *inion* is considered a classic anatomical characteristic of *H. erectus* [68]. However, this condition has also occasionally been observed in Neanderthals, where it is regarded as a *plesiomorphic* character. The external *squama occipitalis* of C5 has a small, shallow, rounded *suprainiac fossa* centrally located between the supreme and superior nuchal lines. This positioning creates a depression in the external occipital protuberance, interrupting the slight and straight occipital *torus* and resulting in a double arch morphology reminiscent of some Indonesian specimens (Ngandong7 and 12). Schwartz and Tattersall [69] describe the occipital *torus* of all Ngandong specimens as having variable prominence, lateral extension to the *asterion*, and two curved nuchal lines that join at the midline to produce a strong external occipital protuberance. Thus, on each side, the lower edge of the “*torus*” appears bow-shaped, which, according to the authors [69], accounts for the double-arched description of the occipital *torus* in the Ngandong specimens. In C5, Ngawi1 and S3, the morphology of the occipital *torus* is similar to the Ngandong specimens, but less prominent [69]. In contrast, C8 exhibits a convex and low occipital plane that flattens into the *suprainiac fossa*, with no occipital bun present. The heavily eroded nuchal *torus* has ectocranial thickening located above the upper nuchal line, and the suprainiac *fossa* at its upper edge helps define the “bilateral arch” morphology. This morphology, which is bilaterally curved and poorly defined laterally, is typical of Neanderthals [45]. The “*torus*” lacks distinct margins, tapering above and below, and the transverse *torus* is thickened medially but not defined laterally. External occipital protuberance is absent. These features together define the “classical” morphology of the Neanderthal posterior *neurocranium* [52].

***Skull thickness***. The results of non-linear metric values constituted by the cranial thickness (Supplementary Tables S5–S8) indicate that C4 and C5 have cranial bone thicknesses that are significantly greater compared to that of classical Neanderthals such as C1. Following in-depth investigations, we found no evidence of a pathological component for this variant in the absence of evident pathognomic osteolithic alterations. Significant cranial thickness has been documented in numerous diachronic and geographically distant human fossil specimens, including the Ceprano *calvarium*, which may have important phylogenetic implications. The cranial thickness of C4 and C5 (Supplementary Tables S5–S7) closely resembles that of Proto-Neanderthals, *H. erectus*, early archaic humans, and Middle Eastern *H. sapiens*. This significant cranial thickness, which is slightly greater in C4, results from an expanded diploic bone and thin inner and outer tables. In both samples, the thickness of the supraorbital *torus* decreases laterally, while the frontal bone is thicker than the parietal bone. The high parietal eminence values of C5 are lower than those of the Australian sample (WLH 50), similar to those of Asian and Indonesian samples, and generally differ from the Middle to Upper Pleistocene and Neanderthals values. Similarly, the elevated bregma thickness values of C4 and C5 are lower than those of the Australian sample (WLH 50), close to those of Asian and Indonesian *H. erectus* (Zhoukoudian, Sangiran, Ngandong) and early archaic humans. These values are similar to those of the Proto-Neanderthal sample Petralona 1, but higher than those of the Neanderthal sample. The thicknesses of C8 are higher than the Neanderthal average (except for Spy 1) and are similar to those Proto-Neanderthals, Indonesian Asian samples, and Arcaic *H. sapiens* (Supplementary Table S8).

***C2, C3, and C6 mandibles***. The fossil specimen C6, represented by a mandibular *symphysis*, exhibits distinctive morphological features. These include the absence of a mental trigone, a slight indication of a chin eminence (*mentum*), and a curvature of the mandible (*incurvatio mandibulae*) visible in lateral view. Additionally, the mandibular *foramen* is positioned horizontally, parallel to the masticatory plane. A comparison between C6 and the C2 and C3 mandibles, discovered between 1939 and 1950, reveals notable morphological variability (Figure 14). In C2, the outlines of the *symphysis* are difficult to discern, since there is no curvature of the mandible (*incurvatio mandibulae*) and no evidence of a mental trigone. The lateral marginal tubercles, which are barely perceptible, are located at the level of the canine to third premolar (C-P3), similar to the *plesiomorphic* condition observed in specimens such as those from Dmanisi. The mental pits and central *keel* are only faintly indicated. C3 and C6 share most features with C2, except for a slight indication of *incurvatio mandibulae*; in C6, this may result from bone atrophy due to tooth loss during life (*intra-vitam*). C6 also has a smaller bicanine width than C3 and closely resembles C2 in this respect. As reported by Vialet et al. [70], the absence of a bony chin is characteristic of the Middle Pleistocene, while the mental trigone—a triangular projection at the front of the mandible—appeared early in the evolution of the *genus*

*Homo*. According to these authors, the mandibles of OH 7 and OH 3 from the Lower Pleistocene possess both the bony chin (*mentum osseum*) and the mental trigone. The mental trigone is also present in KNM-ER 730, S9, and S22, whereas only a small mental protrusion is observed in the Dmanisi mandibles and in specimen ATE9-1 from the Sima del Elefante site (Sierra de Atapuerca). In the Middle Pleistocene, this feature is described in Tighenif 1 and 2 and in the Zhoukoudian mandible. Notably, the symphysis of the Circeo mandibles closely resembles that of the Montmaurin-LN mandibles, displaying a “primitive” configuration that lacks the defining characteristics of the bony chin seen in *H. sapiens*. This condition is typical of most Middle European Pleistocene mandibles, with the exception of two specimens from the Atapuerca-SH site [70]. In mandible C6, the *fossa digastrica* is directed downward and posteriorly, as observed in C2 and C3. This orientation is a pattern documented in Neanderthals, which appears to diverge from the downward-facing *plesiomorphic* model and the generally posterior-facing modern human model [70]. The C6 mandible has a single horizontal oval mandibular *foramen* (Neanderthal-like) on the right side, located below the M1 (P4) position; the left side is incomplete (Supplementary Table S9). In contrast, the C2 mandible, similar to the Montmaurin-LN mandible, possesses two *foramina* on both the right and left sides of the body, with the main *foramina* situated below the M1 position and smaller *foramina* located just beneath them. These paired foramina appear to be connected, separated only by a bony bridge that features a longitudinal groove on the left side. The C3 mandible also exhibits two *foramina* on each side, although the second *foramen* on the left is not clearly visible. The larger *foramina* are positioned below the M1-P4 region, while the smaller ones, as in C2, are located beneath the main *foramina* and are similarly connected by a bony bridge (Supplementary Table S9). The presence of multiple *foramina* is not uncommon in Early and Middle Pleistocene mandibles and is most frequently observed in Pleistocene Asian specimens [70]. In terms of their position, the *plesiomorphic* condition is characterized by *foramina* located more anteriorly (at the P3-P4 level), whereas a *foramen* situated below the M1-P4 or M1 is typical of Neanderthals and most Central European Pleistocene hominins [70]. The mandibles confirm what was underlined for the cranial findings. The three mandibles C2, C3, and C6 show *apomorphic* characteristics, such as the presence of a slight *incurvatio mandibulae* in lateral view (C3 and C6) and the localization of the mental *foramen*, which is in the direction of the *symphysis* between M1 and P4.

***Superior maxillary C9***. Circeo 9 (C9) [Supplementary Figure S10] consists of the palatine process of the maxilla. Its poor state of preservation—being heavily eroded and incomplete—precluded an in-depth comparative morphometric analysis. Osteolytic alterations associated with tooth loss (showing varying degrees of atrophy) and a thickness asymmetry between the two portions of the palatine bone were observed. These findings indicate the need for targeted tomographic analysis, which is beyond the scope of the present study. From an exclusively morphological point of view, we cannot completely exclude a hypothetical relationship with C6, as well as with C2 or C3, a hypothesis that will require future investigations and insights. The only comparative feature that emerges relates to the nasal cavity, specifically the presence of a single, superiorly elevated, midline-grooved spinal ridge. This feature is considered a possible Sima-hominin autapomorphy, with some variants (such as lacking a midline groove) described in Petralona. It is absent in the *H. sapiens* specimens described by Schwartz et al. [46], as well as in *Homo neanderthalensis* (Gibraltar 1; La Chapelle-aux-Saints), *Homo antecessor* (Gran Dolina ATD6-69), the Steinheim *cranium*, and specimens often attributed to *H. heidelbergensis* (Kabwe, Arago, Bodo, Dali, and Jinniushan). This feature is considered as a possible Sima-hominin *autapomorphy* [46]. Neanderthals, and likely Circeo 1, develop a posterior nasal crest that extends superiorly toward the nasal bones, thereby creating an anterior vestibule distinct from the rest of the nasal cavity [46]. In C9, although much of this region is missing, both sides of the nasal cavity preserve a low nasal crest. Based on its location and orientation, we identify this as a posterior nasal crest, but it does not appear to exhibit Neanderthal characteristics. This aspect warrants further investigation from a phyletic point of view.

***Dental sample***. The dental sample consists of a total of seven teeth, of which only two belong to a single individual (upper M^2^ and M^3^, C10a-b). C11, C14, and to a lesser extent C12, show a club-shaped cellular *hypercementosis* that modifies the morphology of the tooth root. Cellular *cementum* is mainly present in the apical thirds and furcation regions, which are thought to be subjected to great mechanical pressure in periodontal physiology [71]. Cellular *cementum* has, among its various functions, the primary role of maintaining the tooth in its *alveolus* and protecting the dental pulp. *Hypercementosis* could therefore be an adaptive response of periodontal tissues toward increasing the support area and the distribution of occlusal forces in coping with excessive occlusal loads. Nevertheless, *hypercementosis* remains a complex multifactorial condition that is not yet clarified and that only in some cases may represent a physiological and compensatory response to the biomechanical environment of the tooth [72]. The general characterization of this dental sample confirms the presence of a mosaic morphology as for the entire human sample from Circeo. In fact, dental traits emerge that are indicative of a Neanderthal *morph* such as middle trigonid crest (C12) and *plesiomorphic* traits by the Middle and Upper Pleistocene, i.e., the buccal vertical groove (C14) [Figure 17; Supplementary Figure S13]. Specifically, we refer to a buccal vertical groove (distal and mesial) present on an upper premolar of Circeo dental specimen (C14) completely similar (the distal) to that recently documented in *H. luzonensis* and *H. sapiens* (Dushan1, 15 Kyr) in Southeast Asia [73,74]). This trait is documented in the premolars of *Australopithecus* and *Homo habilis*, in Neanderthal (Krapina), and in some Chinese *H. erectus* of the Middle and Early Pleistocene (Hexian HXUP3, Yiyuan, Xichuan, and Zhoukoudian) but is absent in Late Pleistocene Chinese samples such as Tianyuan and Xujiayao [73]. The metric results (Supplementary Table S10) support the phenetic relationships suggested by the morphological analyses: the entire dental sample, including C2 and C3, falls within the range of Neanderthal variability, with the exception of C15 (M1) and C14 (P4), which lie outside the variability observed in all hominins except H. luzonensis (M1 and P4 HlCCH6 and P4/P3 HlCCH8). Notably, the crown of C15, although incomplete, is small and compressed in mesio-distal dimension, while the remaining root portion is characterized by small and divergent roots (Supplementary Figure S14), a morphology also observed in dental specimens from Southeast Asia (Luzon and Dushan) [73,74]. This study also provides new *data* on the morphology and endostructure of the mandibular third molar specimen from the Guattari Cave site and results of comparisons with a comparative sample that includes mandibular third molar specimens from *H. erectus*, East and North African *Homo*, Middle Pleistocene European *Homo*, Neanderthals, and fossil and extant *H. sapiens* (Supplementary Table S11). The specimen consists of the two third mandibular molars, C11 and C12, discovered during recent excavations and the third molars included in the two 1939–1950 mandibles, C2 and C3. Comparison between the two dental sub-samples allowed us to define a dental divergent pattern within the same site. The molars C11 and C12 have a medium–high taurodontism index (mesotaurodont), complementary to the Neanderthal lineage, while the molars of the mandibles C2 and C3 have a low taurodontism index (hypothaurodont) [Supplementary Table S12]. According to Kupczik and Hublin [31] and Martín-Francés et al. [33], hypotaurodont molars are also found in the two *Paranthropus species* in Koobi Fora (KNM-ER 1805) and Swartkrans (SK 45 and SK15) while the slender shape of the pulp chambers represents an *autapomorphic* condition in *H. erectus*, penecontemporaries, and Neanderthals. The results of enamel thickness, 3DAET and 3DRET, show a low value for molars C11 and C12 with a 3DAET value shared with Abri Bourgeois-Delaunay (BD1), Abri-Suard (S43), and Krapina (D9). Both fall within the variation range of the Neanderthal group. In contrast, the 3DAET value of molars C2 and C3 is medium–high and is shared with *H. antecessor*, Proto-neanderthal, and Neanderthal. The coronal tissue variables, enamel–dentin-pulp, and the corresponding estimates (Supplementary Table S11) for the enamel thickness components, 3DAET, 3DRET, and Vcdp/Vc, also show divergences. In specimens C2 and C3, the 3D enamel thickness, similarly to the *H. antecessor*, *H. erectus*, and *Homo* specimens from the Middle Pleistocene of Europe (Montmaurin-La Niche and SH), reflect the same thickness pattern as a result of the low percentage of dentine (Vcdp/Vc) in the crown. In contrast, the results of sample C11 and C12 show variables with values higher than the *H. antecessor*, *H. erectus*, and *Homo* samples from the Middle Pleistocene of Europe (Montmaurin-La Niche and SH), which fall within the variation range of the Neanderthal sample reflecting the same thickness pattern. When compared to C11 and C12, molars C2 and C3 show a substantially higher 3DRET value, outside the Neanderthal variability and within the variation range of *H. antecessor*, the European Middle Pleistocene SH specimens including the European Montmaurin-La Niche specimen and of the recent *H. sapiens* group (MH). The enamel of the M3 sample from Circeo falls within the variation ranges of all the groups compared but compared to the individual values, it is rather thin. The proportions of the crown tissue and the distribution of the enamel thickness in the molars of the hominins of the Middle Pleistocene show variations in thickness and topography while the Neanderthals generally have thin enamel. With the exception of specimen C12, the Circeo mandibular M3 specimens exhibit the highest 3D lateral relative enamel thickness (LRET) values among the comparative sample (Supplementary Table S11), overlapping with the maximum values observed in the dataset. The lowest value, found in C12, is similar to that of Krapina (D5) and falls within the average range for Neanderthals. The 3D lateral dentine proportion (LVcdp/LVc) values for the Circeo specimens are generally lower than those of the comparative sample, although there is some overlap with individual specimens across all groups. The chromatic maps of the Circeo mandibular third molars show a distribution pattern that approximates the condition of Neanderthals and Middle Pleistocene European *Homo* (SH), with enamel distributed peripherally along the marginal edges, rather than on the occlusal basin, with a greater concentration at the apex of the lingual and buccal cusps (Supplementary Figures S20 and S21) [33]. The enamel distribution maps highlight a thicker enamel on the lingual and bucco-mesial cusps with a decrease at the bucco-distal level and distributed in C11, C12, and C2 according to a distribution pattern similar to the Neanderthal. The absolute thickness of the enamel approximates the modern human condition. The new evidence confirms, also for the Circeo dental sample, morphometric and endostructural affinities with Neanderthals and other Eurasian hominins of the Middle and Final Pleistocene.

***C16a-b.*** Regarding the postcranial skeleton, some interesting *data* emerge from the analysis of the coxal bones. C16a and C16b are two iliac portions, one left and one right, both exhibiting morphological features typically associated with female pelvises. However, due to observed differences in thickness, size, and overall morphology, we consider it unlikely that they belong to the same individual. In medial view, both C16a and C16b display a single iliac buttress and a supra-acetabular *sulcus*, while the interspinous notch is absent (Supplementary Figures S17 and S18). The *supra-acetabular sulcus* is a *fossa* (*fossa supra-acetabularis*) located between the iliac buttress and the acetabular rim. This feature is also observed in specimens such as SK50 and SK3155, in early representatives of the *genus Homo* (KNM ER 3228, KNM WT 15000), in African fossil samples from the Lower and Middle Pleistocene (OH 28 and Broken Hill E 719), and in European Neanderthals [75,76]. Most Pleistocene hominins are thought to possess a supra-acetabular *sulcus* or *fossa*, including Arago XLIV, OH 28, Skhul IV, and most Neanderthal hip bones such as those from Krapina, Amud 1, La Chapelle-aux-Saints, La Ferrassie I, Hortus XLV, Neanderthal 1, and Sima pelvis 1 [75]. This feature is rarely observed in some Australopithecus specimens (e.g., Sts 14, TM-1605, SK50) and in some modern humans [75,76]. Comparison of C16a and C16b reveals differences in the morphology and thickness of the iliac buttress. C16a exhibits a single ventral acetabulocrystal buttress, considered a *plesiomorphic* feature, while C16b shows a single acetabulospinal buttress similar to that observed in *Australopithecus africanus* Sts14, where the buttress is located near the anterior margin (Supplementary Figures S17 and S18) [76]. Regarding the *genus Homo*, two buttresses are found in specimens attributed to *Homo ergaster* (KNM ER 3228, OH 28), *H. heidelbergensis* (Arago 44, sample Sima de los Huesos), and *H. neanderthalensis* (Neanderthal 1, Kebara 2, Amud 1, La Ferrassie 1, La Chapelle-aux-Saints 1, and Krapina [76]). Among modern human fossils, Qafzeh 9 shows a single ventral acetabulospinal buttress near the anterior edge, while Skhul 4 shows the acetabulocrystal buttress. *H. sapiens* has, when present, a single acetabulocrystal buttress [76]. The only comparable metric values are the height and thickness of the acetabulocrystal buttress. C16a has a lower iliac buttress height (79 mm) compared to Krapina 207 (90.9 mm) [76], but its thickness is greater (19.5 mm in C16a versus 12.4 mm in Krapina 207) [76]. C16a and C16b also differ in thickness, and C16b shows a notable similarity to Omo 1 [75]. The iliac thickness measured at the greater sciatic notch is 23.3 mm in C16b, 22.7 mm in Omo 1, and 20.7 mm in C16a; at the base of the auricular surface, the ilium thickness is 27.3 mm in C16b, 26.4 mm in Omo 1, and 25.7 mm in C16a. Additionally, both C16b and Omo 1 exhibit a thickening of the bone above the acetabulum, corresponding to the base of the Anterior Inferior Iliac Spine (AIIS).

The iliosciatic buttress, defined as the surface extending from the arcuate line to the deepest portion of the greater ischial notch, exhibits a robust, pillar-like morphology in both C16a and C16b, similar to that seen in Krapina 207 [75]. Among fossil specimens, iliosciatic morphology varies: it can be narrow and pillar-shaped (as in SK 3155, SK 50, Arago 44, AT-Pelvis 1, and AT-800), broad and flat (as in AL 288-1), or intermediate (as in Sts 14, KNM ER 3228, OH 28, AT3807-3809, AT3300, and AT1004) [75]. The traits observed in C16a and C16b, including a probable oblique auricular surface (in C16b), a laterally flared iliac blade (in C16b), and robust acetabulocrystal, acetabulosacral, and iliosciatic buttresses, may also be among the features that characterize *H. erectus* [77]. Finally, we note that C16b, in comparison to C16a, exhibits a more complex morphology, characterized not only by notable thickness but also by a reduced height from the center of the acetabular cavity to the iliac crest (approximately 106 mm), which closely resembles the measurement observed in *H. floresiensis* (105 mm) [78].

***C7.*** The C7 femur, which lacks both distal and proximal epiphyses, displays an anteroposterior diaphyseal convexity and a linea aspera with a medio-distal deviation towards the medial side. It also exhibits a probable pilaster (Supplementary Figure S16), a feature thought to be absent in Neanderthals [79], as well as a platymeric sub-trochanteric region and medium cortical thickness. These characteristics fall within the ranges of morphological variation in *H. erectus*, Neanderthals, and modern humans. The periosteal contour is sub-circular. Analysis of the individual C7 measurements and comparison with Pleistocene samples (Supplementary Table S14) shows that the mid-diaphyseal dimensions (M6, M7) of C7 do not exhibit the classic circular morphology typically documented in Neanderthals. The anteroposterior to mediolateral dimensions of C7 are consistent with those documented for some Early and Middle Pleistocene specimens (KNM-ER 737 and 803; Ehringsdorf 5, Kresna11, OH28, Zhoukoudian 1,4-6, Ferrassie 1,2, Quina5, and Tabun1,3).

**Figure 18:**
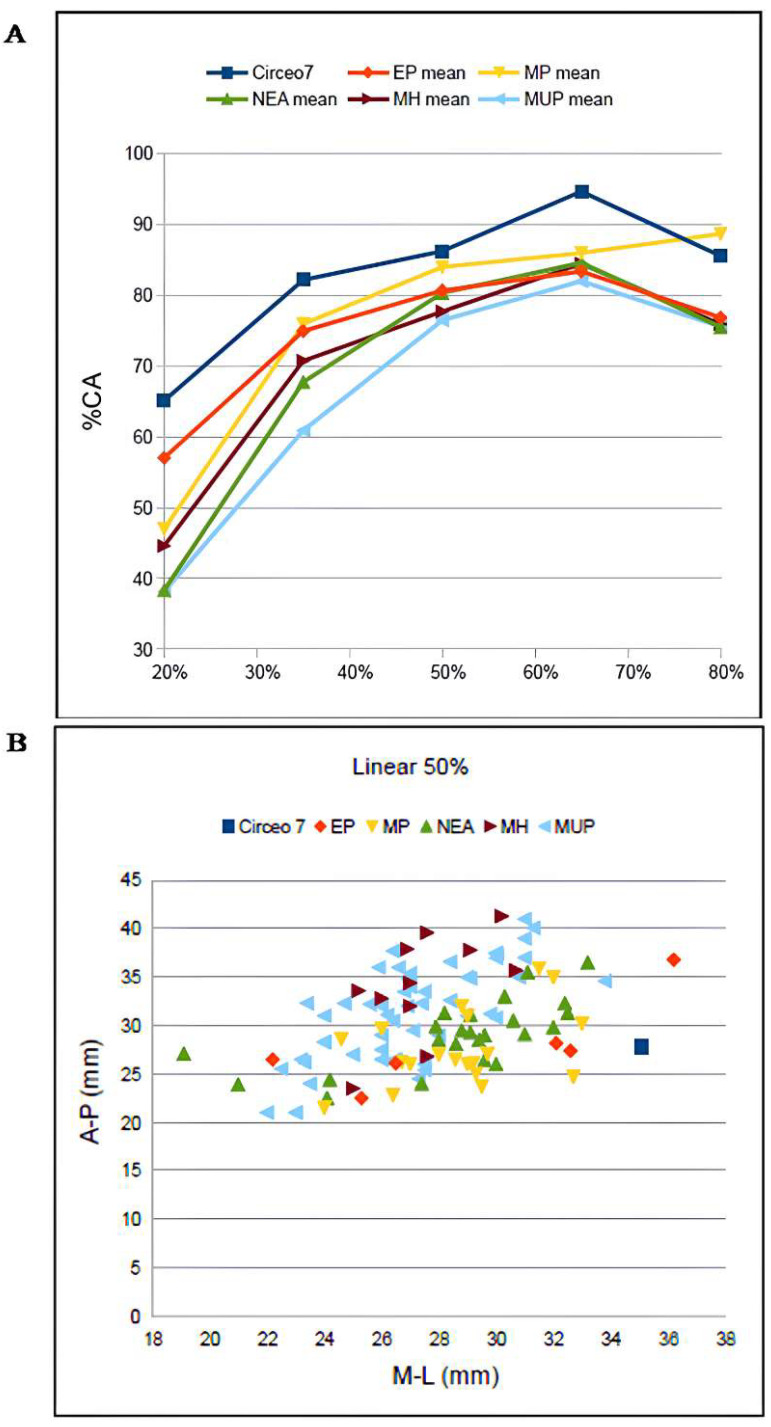
(**A**) Mean percent cortical (%CA) for five diaphyseal sections of the femur Circeo 7 (C7). (**B**) Bivariate plot of the midshaft anteroposterior versus mediolateral external diameters. Abbreviations: **EP**, Early Pleistocene; **MP**, Middle Pleistocene; **Nea**, Neanderthal; **MH**, Middle Paleolithic Modern *Homo*; **MUP**, Early/Middle Upper Paleolithic Middle.

**Figure 19:**
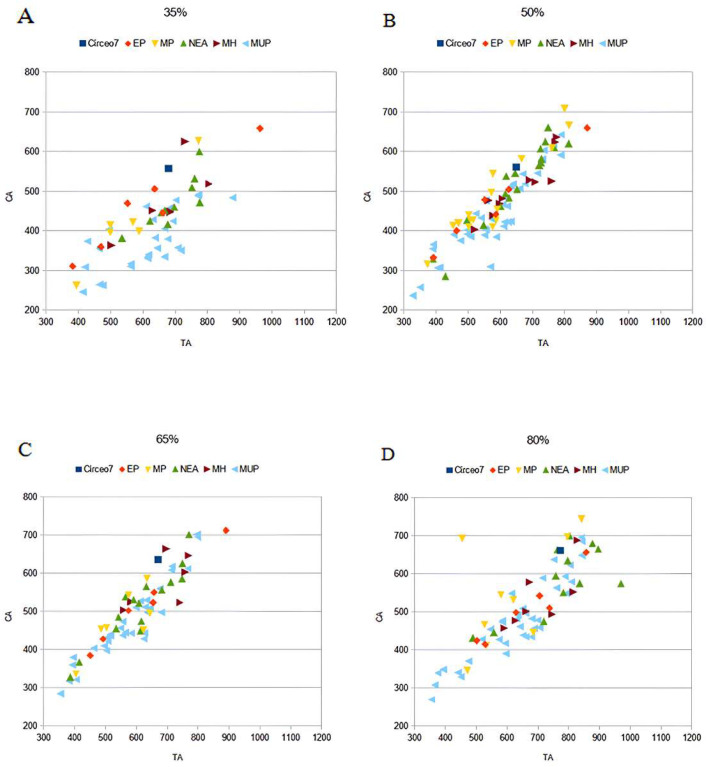
Bivariate plots of cortical area versus total area of Circeo 7 (C7) femur, for the 35% (**A**), 50% (**B**), 65% (**C**), and 80% (**D**) cross-sections. Abbreviations: **EP**, Early Pleistocene; **MP**, Middle Pleistocene; **Nea**, Neanderthal; **MH**, Middle Paleolithic Modern *Homo*; **MUP**, Early/Middle Upper Paleolithic Middle.

The results of the study by Trinkaus and Ruff [80] re-evaluating the transverse diaphyseal femoral properties of Pleistocene humans show that there was a variably significant decrease in relative cortical area during the Pleistocene, especially in the central portions of the femoral diaphysis compared to Upper Paleolithic humans. The plots of mean %CA values (Figure 18A) indicate, consistent with Trinkaus and Ruff [80], that there is considerable variability; however, C7 and the Lower and Middle Pleistocene samples (Early Pleistocene, EP, and Middle Pleistocene, MP) exhibit the highest %CA values. In contrast, the Early/Middle Upper Paleolithic (MUP; which includes Middle Paleolithic Modern Humans [MPMH] and Early/Upper Middle Paleolithic Modern Humans [EUP/MUP]) sample consistently shows the lowest average %CA. However, at the 65% cross-section, the Neandertals approach the high value for the Middle Pleistocene sample, and at the 80% cross-section, only the C7 specimen stands out from the other five samples. C7, when compared to the distribution of cortical area relative to total subperiosteal area across cross-sections from 35% to 80%, aligns with the comparison samples. According to Trinkaus and Ruff [80], these data show a significantly greater spread, with significant differences between samples observed only at the 35% and 50% cross-sections (Figure 19). At the 35% cross-section, the lower values observed in the MUP sample account for the significant difference, while at the 50% cross-section, the high values of C7 and the Middle Pleistocene specimens are responsible for the significant difference compared to the pre-MUP sample. The high %CA appears to be a characteristic of Pleistocene *Homo femora* [80], and the C7 values would seem to place it within it. The transverse diaphyseal femoral properties show high mean values, particularly at the 50% and 80% cross-sections. The mean ratios of the anteroposterior to mediolateral area second moments distinguish the Early Pleistocene sample, which has consistently lower ratios, from the Middle Pleistocene and Neanderthal samples (including C7), which have similar ratios. In contrast, early modern human samples have higher ratios, resulting in a substantial contrast between archaic *Homo* and early modern human *femora* [80]. The Imax/Imin indices for the second moments of area at the midshaft of C7 are consistent with those observed in Castel di Guido, Zhoukoudian, and Neanderthal specimens (Ferrassie 2, Fond-de-Forêt 1, Saint-Césaire 1, and Tabun 1). At the mid-shaft, C7 shows no reduction in mediolateral diaphyseal strength (second moment of mediolateral area, Iy) relative to anteroposterior strength (second moment of anteroposterior area, Ix) and exhibits little or no change in anteroposterior reinforcement. When individual values for midshaft external diameters are compared (Figure 18B), significant differences persist across samples. Although some overlap exists, the early modern human samples (MUP and MH) have higher values than the archaic human distributions (EP and MP), while C7 and the Early Pleistocene (EP) sample have the lowest values. Overall, the three archaic samples display considerable variation, with C7 falling outside the range of all human distributions compared. Based on the overall morphometric results, we hypothesize a phyletic relationship with Lower/Middle Pleistocene hominins and Neanderthals, placing C7 within the range of variation observed in European Neanderthals.

***Multivariate analyses.*** In association with the morphological analyses, statistical analyses were performed by investigating two groups of variables: linear measures and thicknesses. To analyze the relationships of this pair of variables, a multivariate statistical analysis was performed using principal component analysis (PCA).

**Figure 20:**
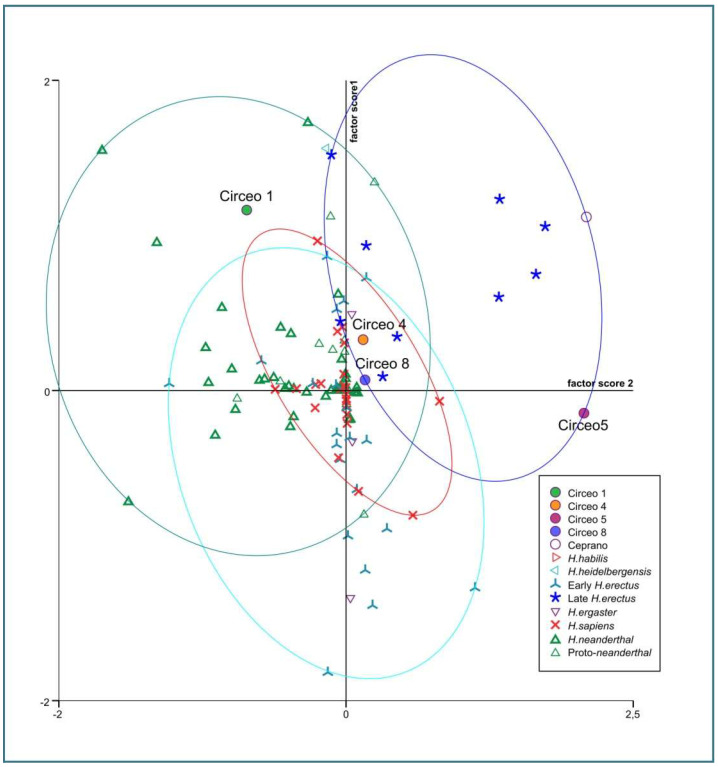
Principal component analysis (PCA) showing the distribution of Circeo 5, Circeo 4, Circeo 8, Circeo 1, Ceprano, *H. habilis*, other Neanderthals and Proto-Neanderthals, *H. erectus*, and *H. sapiens* using all measurements (23 variables). The ellipses indicate the groupings. The *H. erectus* sample conforms to the non-metric morphological results and occupies a space that does not overlap with the Neanderthal model in the PCA. The overall observed pattern of separation between Neanderthal and *H. erectus* specimens is consistent with that described. Circeo 5 differs from all other Neanderthal specimens, including Circeo 1, in that it plots at the extremity of the Neanderthals range on PC1, at the boundary between Early *H. erectus* and Late *H. erectus* (which includes Circeo 4, Circeo 8, and Ceprano). Its position on the plot may indicate high levels of variation in late Neandertals and its taxonomic attribution may not represent a classic Neanderthal.

The exploratory factor analysis showed a Kaiser–Meyer–Olkin (KMO) test of sample adequacy with a value of 0.744, which indicates sufficiently acceptable sampling (range 0.7–0.8). Furthermore, the Bartlett test of sphericity is highly significant (approximation X2 = 1465.4 with 253 degrees of freedom and *p* = 0.001), indicating that the correlations are sufficiently high to not be comparable to 0. The extraction of the principal components suggested the use of 2. The eigenvalues indicating the amount of variance explained by each principal component relative to the first two principal components have values of 5.554 and 3.791, respectively. Considering that eigenvalues greater than 1 are considered significant, the result indicates that the components explain more variance than a single original variable. Since the first two components with eigenvalues well above 1 suggest that they are good and significant, the value of the total variance explained by the first two principal components is 40.63%. This result can be considered good. In general, a higher value is preferable because it indicates that a greater amount of variability in the original data is captured by the principal components. However, in our case, obtaining a value around 40–50% can be acceptable, especially considering that the data is complex and multidimensional. From the resokult, it is clear that Neanderthals are grouped together, showing, as expected, modest variability (Figure 20) also in comparison with Proto-Neanderthals and *Heidelbergensis*. The same goes for *H. erectus*, which, although differentiated between early and late, tends to concentrate in two distinct quadrants. The early (Dmanisi, Asian, and African *H. erectus*) show affinities with *H. habilis* and *Homo rudolphensis*, while the recent ones (Indonesian *H. erectus*: Ngandong group) are rather limited and Ceprano is also placed within them. The position of Circeo 1 appears fully inserted in the classical Neanderthal context, while that of Circeo 4, 5, and 8 appears more problematic. Circeo 4 and 8 are contextualized between Proto-Neanderthal, Neanderthal, and *Sapiens*, while Circeo 5 appears rather detached, confirming the morphological observations and positioning itself between Late *H. erectus* and *H. sapiens*.

## 5. Discussion and Conclusions

### 5.1. Population Variability and Composition

In this study, we examine newly discovered hominin remains from Grotta Guattari, dated to before 40,000 years ago, within the broader paleo-demographic context of Middle and Late Pleistocene Eurasia. Our analysis focuses on their morphological variation and affinities, with the goal of clarifying their phyletic relationships. Most of the hominin remains were recovered from the internal areas of the cave (“Antro del Laghetto” and “Antro dell’Uomo”), while two teeth (C10a, b) and a mandible (C3) were found in an external fault that shares the same stratigraphic context as the internal area.

#### 5.1.1. Affinities with Neanderthals and Pre-Neanderthals

Analysis of all the human remains (both I and II findings) reveals substantial morphological variability, including a “classic” Neanderthal morphotype (C1) and other specimens exhibiting mixed anatomical features indicative of distinct morphs or taxa. This observed variability suggests the possible coexistence of multiple morphs at the site. The overall analysis of the sample shows traits shared with a *clade/taxon H. erectus s.s.* and *s.l*., Proto-Neanderthal, Neanderthal, Late *H. erectus*, and *H. Sapiens*. The C1 skull displays all the classic morphological features characteristic of recent Neanderthals, such as those seen in Feldhofer, La Ferrassie, La Chapelle-aux-Saints, and La Quina. In contrast, the cranial remains of C4, and particularly the C5 *calvarium*, exhibit a complex mosaic of *plesiomorphic* and *autapomorphic* traits, complicating their classification within the group of classic Neanderthals. In C4 and C5, the supratoral region closely resembles that of Biache-Saint-Vaast 2, as well as the Late European *Erectus* specimen from Lazaret 24. However, the mandibular *fossa* of C5 differs from those of Middle Pleistocene European fossils (Ceprano, Castel di Guido, SH, Steinheim, Petralona, and Ehringsdorf), which typically display an *autapomorphic* flat articular eminence and a protruding postglenoid process [41], both of which are absent in C5. Additionally, in the mastoid region, all Neanderthal specimens—including C1 and C5—share the *plesiomorphic* trait of an anteriorly obliterated digastric *sulcus*, as seen in Middle Pleistocene European fossils such as Ehringsdorf H, La Chaise (Suard), and Biache-Saint-Vaast 2. While “classical” European Neanderthals, like C1, possess an anterior mastoid tubercle (an autapomorphy), this feature is absent in C5, as well as in earlier Asian or European Neanderthal specimens [41,62].

#### 5.1.2. Possible Connections with Distant Populations

The morphological similarities between C5 and *H. erectus s.s.* are primarily related to the presence of a frontal *keel*, as described in the Trinil 2 holotype, while affinities with *H. erectus s.l.* are associated with the morphology and certain features of the mandibular *fossa*. According to Martínez and Arsuaga [41], two *autapomorphic* traits can be used to characterize the temporal bone of *H. erectus*: I) a strong reduction in the *postglenoid* process, and II) the absence of the styloid process; both are absent in C1 but documented in C5. Furthermore, the morphology of the temporomandibular joint in C5 closely resembles that of Indonesian specimens (Javanese *H. erectus p-deme*), suggesting a possible phenetic relationship with Late *Erectus* from Southeast Asia. This feature is considered distinctive of the Ngandong specimen, with which the Circeo specimen shares several other cranial morphometric characteristics. Taxonomic analysis of the occipital bones of C5 and C8 reveals the presence of *plesiomorphic* traits shared with *H. erectus* that are rarely found in Neanderthals, as well as the absence of a well-defined “occipital bun”, a feature typical of classic Neanderthal morphology. Specifically, C5 exhibits an esocranial configuration similar to that described in some Indonesian specimens (Ngawi1, S3, and Ngandong), whereas C8 displays features characteristic of classic Neanderthal morphology. Analysis of the dental sample reveals similar variability and affinities with South Asian lineages and Neanderthals, particularly due to the presence of *plesiomorphic* traits such as the “buccal vertical groove” and the anterior fovea associated with the mid-trigonid crest. The buccal vertical groove is a rare trait found on the upper premolar C14 and documented in Chinese *H. erectus* of the Lower-Middle Pleistocene (Zhoukoudian, Xichuan, Hexian, and Yiyuan) in Neanderthal (Krapina), in Late Southeast Asian *H. erectus* (*H. luzonensis*), and in *H. sapiens* (Dushan 1) [73,74]. Therefore, in analogy with the entire human skeletal sample, the Guattari Cave dental sample also shows a mosaic of traits shared with *H. erectus*, Neanderthals, and *H. sapiens*. Traits of the Middle Eastern (Israel) archaic *Sapiens* emerge in the vertical parieto-temporal morphology of C5 very similar to Manot1 [52] and in the coxal bones (C16a, b) characterized by a single iliac buttress, as documented in the Skhul/Qafzeh group [76]. The mandibular *symphysis* of C6, similar to that of C3, exhibits apomorphic features such as a slight *incurvatio mandibulae* and the *mental foramen* located between M1 and P4, traits common in Neanderthals (including C2). In contrast, C2 displays a *plesiomorphic* configuration, similar to most Middle Pleistocene European mandibles. These mandibular features corroborate the findings from the cranial remains. Additionally, the M3 dental sample from Circeo exhibits morphometric and endostructural affinities with Neanderthals and other Eurasian hominins from the Middle Pleistocene. The distribution pattern and absolute enamel thickness are similar to the Neanderthal model in the former case, and to the modern human condition in the latter. The overall results for the C7 femur place it within all Middle Pleistocene human distributions and within the range of variation in European Neanderthals. In this study, we used cranial metric data to infer the taxonomic ancestry of the Circeo specimens relative to the species compared. The principal component analysis (PCA; Figure 20) produced a complex pattern, as expected given the wide chronological and geographical distribution of the fossils. Notably, affinities with Asian *H. erectus* emerge, even though the samples originate from geographically distant and isolated areas such as Java. Despite these limitations, several relevant aspects can be identified. The PCA distinguishes at least three main groups: (1) Late Asian *H. erectus* specimens, including the European Ceprano specimen; (2) early *H. erectus* specimens, such as those from Dmanisi, as well as Asian and African *H. erectus*; and (3) Proto-Neanderthal and Neanderthal specimens. Circeo 5 appears to fall at the boundary between the two *Erectus* groups and is distinct and distant from the Neanderthal group, in which Circeo 1 is fully included. Circeo 8 and Circeo 4 are closely positioned: Circeo 8 lies between the Neanderthal and Proto-Neanderthal groups, while Circeo 4 is situated between the Late *Erectus*, Proto-Neanderthal, and *Sapiens* groups. The metric variables are the parameter that influences the differences in shape, size, and thickness of each sample and the PCA reveals for the Circeo sample a morphological space characterized by a variability in shape and thickness, providing results similar to the morphological variability that emerged. Our analysis reveals a clear divergence within the sample: Circeo 1 is firmly situated within the classic Neanderthal context, whereas the other specimens present more complex taxonomic implications due to their combination of mixed metric and non-metric traits, including both *plesiomorphic* and *autapomorphic* features. These findings suggest affinities with various morphological models and point to a potentially ancient taxonomic origin for these specimens.

### 5.2. Evolutionary Scenarios and Paleogenomic Implications

The Circeo sample shows *autapomorphic* features independent of *H. erectus* and *plesiomorphic* features shared with Proto-Neanderthals, Neanderthals, Late *Erectus* of Southeast Asia, and *Sapiens*, which suggests morphological affinity between these *taxa*. The morphological qualitative *data* finds a positive response in the quantitative metric results (Figure 20), which place the entire Circeo sample in a mixed but geographically well-defined context between Europe and Asia, as was predictable, but with a new and unexpected extension towards Southeast Asia.

The human remains from Grotta Guattari display a variability that may indicate the coexistence of multiple morphs or *taxa* at the site. Two main chronological phases are relevant: an older phase, dated to approximately 120 ka [10,11], associated with the external atrial area of the cave (which shows evidence of structured human activity but lacks skeletal remains), and a more recent phase, dated to about 65–66 ka [10], involving both the internal and external areas and yielding human remains. These chronologies suggest that the site was occupied at different times (MIS5.5, MIS5d, and MIS4) by multiple human lineages, including Neanderthals. Based on these findings, we can propose interpretative hypotheses regarding the evolutionary relationships and migratory dynamics of hominins in Eurasia during the Late Pleistocene.

**Figure 21:**
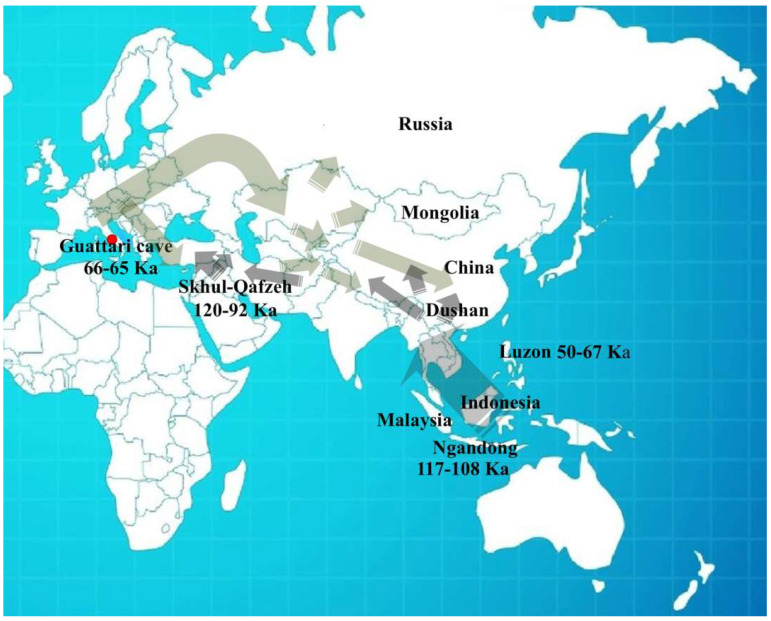
Expansion models hypothesis of the Middle and Final Pleistocene. I dispersal, oldest, Asia→Europe (117–108 ka) and II late and rapid dispersal, Europe→Asia→Southeast Asia (66–50 ka).

During the Pleistocene, Java experienced relative isolation from the Asian mainland due to cyclical glacial and interglacial phases [47], which alternately facilitated or hindered the crossing of geographical barriers and influenced human evolution in both insular and continental contexts. As suggested by Zanolli et al. [81], while it is possible that hominins (at least *H. erectus* and *H. floresiensis*) reached the Philippines via land bridges (Sonda–Palawan–Luzon) during glacial periods of the Lower and Middle Pleistocene, it is also plausible that reverse migrations occurred, with trajectories extending from Southeast Asia to continental Asia and ultimately to Europe. The evidence for human presence at Guattari Cave between 120 ka and 66 ka coincides with the broader period of occupation by Javanese *H. erectus* on Luzon Island (66.7–50 ± 1 ka [73]) and at Ngandong (117–108 ka [82]). The chrono-morphological similarities between these Indonesian human samples and those from Circeo, along with the presence in both of unique traits documented in *H. erectus* (*s.s.* and *s.l.*) and *plesiomorphic* traits also found in European Middle Pleistocene hominins, may suggest new migratory scenarios and inform novel models of human evolution and dispersal (Figure 21).

**Hypothesis scenario 1.** Late *H. erectus* may not have become extinct in Ngandong [82] and the *Sapiens* hominins Skhul/Qafzeh may not represent an extinct lineage [83].

Based on the chronological data relating to the Ngandong group (117–108 ka, [82]) and the Skhul/Qafzeh (Israel) group (92 ± 5 ka and 120–80 ka, [84]), the human remains from Circeo could indicate the presence of both Javanese *H. erectus p-deme* and Middle Eastern *Sapiens* (Israel) on the European continent. This evidence may support the hypothesis of contact during a first (I) dispersal from Asia to Europe.

**Figure 22:**
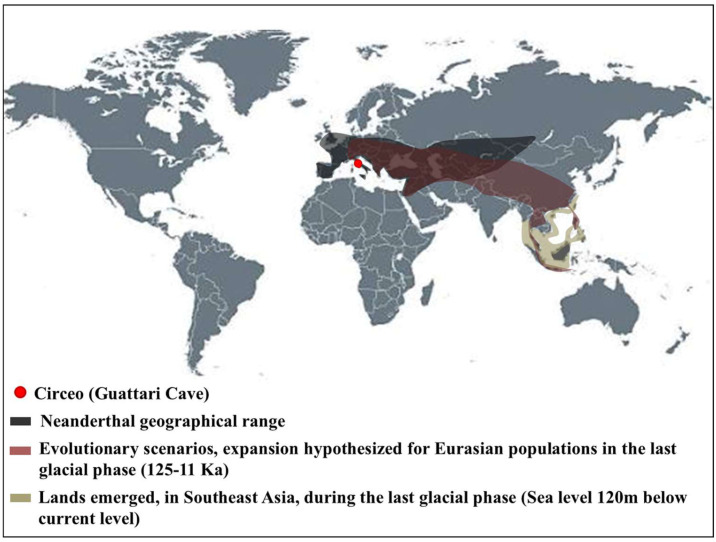
The range of Neanderthals and the hypothesis of expansion of Eurasian populations including Southeast Asia. Neanderthal geographical range [38]; Land emerged [86].

**Figure 23:**
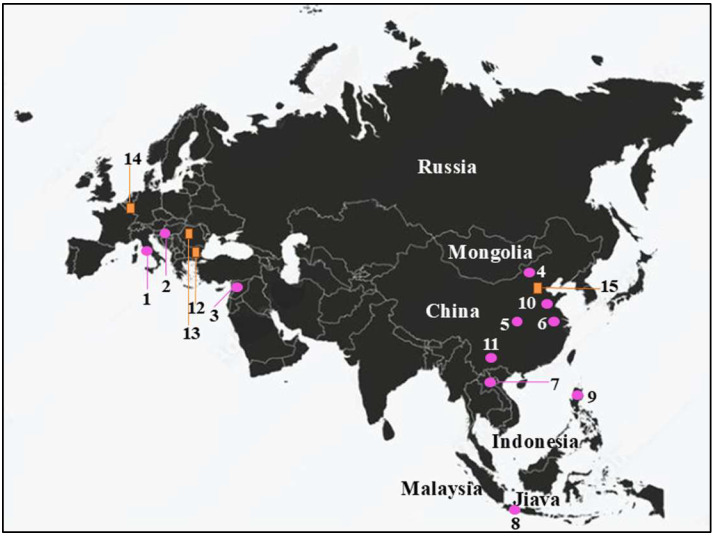
Localization and attempted correlation between the morphological traits (epigenetic) discussed in the text and the genomic *data*. According to the results of Hajdinjak et al. [38], analyses of the genomes of Neanderthals and modern humans have shown that gene flow occurred between the two hominin groups approximately 60–50 ka BP, probably in Southwestern Asia. When comparing the Bacho Kiro Cave individuals to present-day populations, Hajdinjak et al. [38] found that three of these individuals share more alleles (that is, more genetic variants) with present-day populations from East Asia, Central Asia, and the Americas than with populations from western Eurasia. Groups related to the Bacho Kiro Cave individuals contributed to later populations with Asian ancestry as well as some western Eurasian humans such as the Goyet Q116-1 individual in *Belgium*. Xichuan (not dated) from Xing et al. [87]. **Morphometric Data** (pink round)**: 1.** Circeo, Gattari Cave (Italy), 121–105 ka human presence: 59–51 ka, Circeo 1-3 Neanderthal remains and 66–55 ka, Circeo 4-16b **present study** remains; **2.** Krapina (Croatia) 130–123 ka, Neanderthal remains; **3.** Skhul-Qafzeh (Israel) 120–92 ka, *H. sapiens* remains; **4.** *Zhoukoudian*, (China) Early–Middle Pleistocene (770–230 ka), *H. erectus* remains; **5.** Xichuan (China) age unknown (from Mid-Middle to Late-Middle Pleistocene), *H. erectus*; **6**. Hexian, Middle Pleistocene (412 ± 25 ka), *H. erectus*; **7.** Tam Ngu Hao (TNH2-1) [Southeast Asia] 164–131 ka, Neanderthal-Denisova; **8.** Ngandong (Indonesia) 117–108 ka, Late *H. erectus* (*evolutus*)—Sangiran, Early Pleistocene *H. erectus*; **9.** Luzon, (Indonesia) 67–50 ka, Late *H. erectus* (*evolutus)*; **10.** Yiyuan (China), Late Pleistocene *H. sapiens*; **11.** Dushan (China), 15 Kyr H. sapiens. **Genome Data and interbreeding *H. sapiens* [38]** (orange square): **12.** Bacho Kiro (Bulgaria) [F6-629; BB7-240; CC7-335], 45–42 ka, 3–3.8% Neanderthal DNA; **13.** Oase 1 (Romania), 42–37 ka, 6.8% Neanderthal DNA; **14.** Goyet (*Belgium*) [Q116-1], ~35 ka; **15.** Tianyuan (China), ~40 ka, interbreeding Neanderthals/ancient and present-day East Asian populations [73].

Contact between *Erectus*, southern Neanderthals/Denisovans, and *Sapiens* has already been documented at Tam Ngu Hao 2 [85], with genetic evidence indicating the eastward dispersal of Neanderthals into the Russian Altai during the Late Pleistocene. These findings expand the geographical scope of migratory dynamics, suggesting that Central Western Europe was also involved in exchanges with Asia and Southeastern Asia (Figure 21, Figure 22 and Figure 23).

**Hypothesis scenario 2.** If the datings are exact, the chronological difference between Circeo (~66–65 ka and ~59–51 ka), Luzon (50–67 ka, [81]), and Manot 1 (54.7 ± 5.5 ka, [88]), associated with the individual variability found, would allow us to hypothesize, in this final stage of the Pleistocene (approximately between 66 and 50 ka), possible local interbreeding in Central Western Europe with a subsequent phenomenon of dispersal (II dispersal) towards Eastern Europe to Eastern and Southeastern Asia. In the Grotta Guattari site, there is a classic Neanderthal *morph* (C1), and *morphs/taxa* distinct from this characterized by a mixture of morphological and metrical characters. The hypothesis of a mosaic-like variability model is therefore outlined, which could represent the final result of evolutionary relationships and migratory dynamics between Neanderthals, *Erectus s.l.* (Late Javanese and European), and *Sapiens*. It is believed, in support of this hypothesis, that hybrid populations are characterized by high degrees of individual variability [89,90,91].

The hypothesis of these scenarios proposes, for the Final Pleistocene, the possibility of complex demographic models, with the possible survival of archaic populations, and migratory and dispersion processes from Europe towards Southeast Asia and vice versa. Movements and genetic flows during this period were likely influenced by the severe glacial climatic conditions of MIS 4, which would have simultaneously favored the formation of geographical connections through land bridges [81] (Figure 21 and Figure 22).

**Hypothesis scenario 3**. Excluding the hypotheses of scenarios 1 and 2, it is however possible that the mosaic of *plesiomorphic* and *autapomorphic* traits that emerged on the human sample from Circeo reflects contact between different *clade/taxon*. Such contact, even if it did not occur precisely in this context, must have taken place during the first half of the Late Pleistocene (I and II dispersal) along the route between Europe and Eastern and Southeastern Asia. A vast geographical area which has already been placed at the center of attention by molecular studies and whose results have opened up new interpretative fronts regarding migratory and evolutionary dynamics, with interbreeding and genetic flows, has led these populations of the Final Pleistocene to modern humans. The genetic results confirm, in fact, the presence of gene interbreeding between these continental and insular populations of Southeastern Asia, Denisovans and Neanderthals, results which have allowed us to hypothesize the presence of various migratory waves in the Late Pleistocene whose modalities still remain unknown [92,93,94,95,96,97,98].

Genetic studies on ancient and modern mitochondrial DNA (mtDNA) have documented the existence of important Eurasian population turnover in the Late Pleistocene [99]. From the results of these studies, it emerges that all current non-Africans belong to two basal mtDNA haplogroups (hgs), M and N [99]. The M lineage is absent in contemporary Europeans but occurs at high frequency in modern Asians, Australians, and Native Americans [100]. However, analysis of 55 European mitochondrial genomes [37] revealed the presence of the M lineage in individuals predating the last Glacial Maximum, a discovery with important implications for the timing of modern human dispersal in Eurasia, potentially including the Circeo sample. The time to the most recent common ancestor of each of these two clades was estimated [101] independently at ~50 ka (95% confidence interval [CI], 53–46 ka) and at ~59 ka (95% CI, 64- 54 ka), respectively, a time surprisingly very close to the chronology estimated for Guattari Cave and almost overlapping with that of Luzon. This time range was later narrowed [37] to between 44 and 55 ka, which coincides with the presence of the first modern humans in Australia and Europe [37]. The interpretation of these results has been read as (i) evidence of an early spread of modern humans, hg M, to Asia via a probable southern route followed by a subsequent spread, hg N, via a northern route [102]; (ii) evidence of a unique pattern of Eurasian dispersal that first reached Asia and then western Eurasia after the loss of hg M [103]; (iii) evidence of a unique, late, and therefore rapid dispersal of a population originally containing both M and N hgs, which contributed to all the mitochondrial diversity of current non-Africans [100]. The authors [37] attribute the loss of hg M to demographic changes that took place later within Europe. According to their findings, the initial expansion occurred before the diversification of M and N, with subsequent migrations introducing both lineages into Europe [37]. Although the hominins of Circeo and those of Southeastern Asia share similar time intervals, they are separated by vast geographical distances, an incongruity that may be explained by the results of this study [37]. These results would seem to validate what emerged from our study by providing genetic confirmation (mtDNA) to the existence of contacts and crosses between lines, diachronic and synchronic, very distant geographically. Specifically, the expansion models of the final phases of the Pleistocene proposed by Posth et al. [37] are in line with the models (hypothesis scenario 1 and 2) that emerged from our analysis, which predicts a first and oldest Asia→Europe dispersal of basal haplogroups M and N, with a loss of M probably due to the presence of numerous *lineages* in Eurasia, but found in the mtDNA of *Belgium* and France [37], and a second late and rapid dispersal Europe→Asia→Southeastern Asia, where M pre-existed, and where *lineage* is present with high frequency in modern Asians and Australians. Genomic evidence, therefore, documents with certainty the existence of migratory and evolutionary dynamics in the Late Pleistocene, and above all the existence, in Eurasia, of gene flows capable of covering large latitudes and overcoming geographical barriers extending to the island areas of Southeast Asia. The morphometric diversity found in the Circeo sample could be relevant for discussions of the morphological diversity of the first modern humans who spread to Europe during the Late Pleistocene and the relationships between Neanderthals, the first modern humans in Europe, and subsequent populations of Eurasia [38] [Figure 23].

### 5.3. Broader Significance for Paleontology in the Mediterranean and Eurasian Environment

In the reconstruction of the dispersions and evolutionary trajectories of *Homo* in Eurasia in the Final Pleistocene, the identification of traits indicative of a Javanese *morph* in the Neanderthal context of Circeo could be relevant for discussions of evolutionary scenarios in the first half of the Final Pleistocene. The first point to consider is the close chrono-morphological similarity observed between the Circeo sample and the last Indonesian *Erectus*, including *H. luzonensis*. This similarity could prompt a re-evaluation of the hypothesis regarding the extinction of *H. erectus* in Southeast Asia. It is believed that the hominins of Southeast Asia, in the final stages of the Pleistocene, lived in a sort of insular isolation until extinction [82], but this may not be the case. The late discovery [85] of a Denisovan molar (TNH2-1) in the Tam Ngu Hao2 Cave in Southeast Asia, which presents Neanderthal traits (mid-trigonid crest), indicates the possibility that a phyletic link exists between Neanderthals and meridional Denisova of Southeast Asia [85] and therefore between Europe and Southeast Asia. In confirmation, genetic studies on ancient and modern human mitochondrial DNA (mtDNA) have revealed the existence of important turnover of the Eurasian population in the Late Pleistocene [37]. In the absence of molecular findings, searching for combined features in the human fossil record from Circeo may help define this as an alternative hypothesis on human movements in Eurasia.

Between 120 and 110 ka (after the final stage of the MIS 5.5), the Late *Erectus* of Southeast Asia may have come into contact, in continental Asia, with the *Erectus*-Neanderthal/Denisovia (Altai and meridional) and early *Sapiens* who circulated in the geographical area which extends from the Altai mountains to Central–Eastern Europe up to the extreme Southeast Asia. The mixture of distinct traits, documented in the Circeo sample, could be relevant for discussions of migratory flows in the first half of the Late Pleistocene in Eurasia, including Southeast Asia. This hypothesis foresees the possibility that a first and older dispersal from Asia → Europe and a second late and rapid dispersal from Europe → Asia → Southeast Asia may have occurred (Figure 21). The morphological results and combined metric results (PCA) of the overall Circeo sample highlight the presence of a classic Neanderthal morphology (C1), atypical and rare *Erectus* traits in a Neanderthal context mixed with typically Neanderthal traits and a morpho-metric divergence of C4 and C5 compared to C1. The combined morphological and metric results of the overall sample from Circeo highlight a high degree of individual variability, which could be relevant for discussions of possible interbreeding between multiple lineages and dispersions in a late phase of the Pleistocene. Genetic *data* suggest that Denisovans interbred with modern humans, Neanderthals, and an unknown third archaic hominin *lineage*, perhaps a late surviving *H. erectus*, although its identity remains unclear [94,104]. Among the archaic or *post-erectus* hominins that could represent the Denisovans [105], Xuchang [106] and the Maba have been reported. The Xuchang skull presents a mosaic of traits similar to *H. erectus* and of Neanderthal, and the Maba skull shows some typical Neanderthal traits [107] and a morphology considered congruent with the genetic proximity of Denisovans and pre-Neanderthal populations [108,109]. According to Kaifu [1], the archaic Denisovans lived over a wide geographical and ecological range, from Siberia to tropical Asia, and according to the author, there is no doubt that the first settlers of Sahul were descendants of populations who migrated using the southern routes of the Himalayan mountains in the Late Pleistocene. Considering the existing controversy on the times and paths followed by populations *pre*-modern human and the vast geographical area taken into consideration, we believe that a Central Western European involvement within this turnover could be plausible. A turnover which involved, in the Final Pleistocene, the last Asian *Erectus* (Indonesian), the Denisovians (Altai and Meridional), Neanderthals, and archaic Middle Eastern *Sapiens* in multiple dispersals to and from Southeast Asia, as demonstrated by mitochondrial DNA results [37] and Denisovans admixture (southern) detected in extant Australo-Melanesian populations [1]. The Circeo human sample, based on its combination of *plesiomorphic* and *autapomorphic* traits, could represent an important pre-40 ka specimen for understanding the migratory and evolutionary dynamics of the last Neanderthals and *Erectus*, as well as their phyletic connections with early *Sapiens* and modern Euro-Asian populations. The PCA supports this statement and shows morphological affinities between Europe and Asia of the Final Pleistocene and modern humans, which has already emerged and been documented by numerous genetic *data*. The long-term presence of *H. erectus* and Neanderthals in Eurasia suggests that they were meta-populations, with a long and undoubtedly successful biological history, having survived multiple ice ages with climatic fluctuations and related climatic–environmental changes that represented an uphill battle for survival. The hominins who resided in Guattari Cave between 120 and 60 ka could represent a population influenced by a first wave of migration towards Central Western Europe and the native populations that populated the region. It was undoubtedly a dynamic meeting of different genetic flows, in which a unique territorial adaptation to a climatically and nutritionally advantageous area can be observed. The new population, belonging to a Neanderthal time horizon and found in the Guattari Cave site, could have been affected by climatic–environmental conditions and land bridges, which are relevant factors in discussions of dispersal and genetic diversity of the first modern humans who spread across Eurasia during the Late Pleistocene [38]. The morphological model that has emerged demonstrates variability among Eurasian hominins of the Final Pleistocene, with evolutionary implications that warrant further investigation, as they may help define new geographical and phylogenetic pathways. We therefore believe that the Circeo hominins, representing a population sample of the final phases of the European Pleistocene (before 40 ka), have the value of contributing to the debate on the evolutionary dynamics and migratory scenarios of Eurasian populations during the Middle and Final Pleistocene.

## Data Availability

Data are available in the manuscript and Supplementary Materials.

## Supplementary Materials

The following supporting information can be downloaded at: https://www.mdpi.com/article/10.3390/genes17020132/s1, Comprehensive supplementary catalog of specimens. Supplementary Figure S1: Internal path of the cave. Supplementary Figure S2: Interior of the cave and excavation area. Supplementary Figure S3: Three-dimensional reconstruction of Circeo 7 (C7) femur. Supplementary Figure S4: Circeo 12 (C12) permanent lower left third molar (LLM3), volumetric bifurcation index (VBI). Supplementary Figure S5: Circeo 4 (C4) and Circeo 5 (C5). Supplementary Figure S6: Circeo 4 (C4), coronal suture. Supplementary Figure S7: Circeo 5 (C5) *basicranium*, asymmetry of the *supratubalis* process. Supplementary Figure S8: Circeo 5 (C5), temporal bone left *petrous* part (arborizing sigmoid *sinus*). Supplementary Figure S9: Circeo 8 (C8), occipital bone (*squama occipitalis*). Supplementary Figure S10: Circeo 9 (C9), palatine process of maxilla. Supplementary Figure S11: Upper teeth (maxillary). Supplementary Figure S12: Lower teeth (mandibular). Supplementary Figure S13: Circeo 14 (C14), permanent upper right second premolar (URP4), 3D reconstruction. Supplementary Figure S14: Circeo 15 (C15), permanent upper right first molar (URM1). Supplementary Figure S15: Circeo 7 (C7), right femur (diaphysis). Supplementary Figure S16: Circeo 7 (C7), right femur (pilaster). Supplementary Figure S17: Circeo 16a (C16a), left coxal bone. Supplementary Figure S18: Circeo 16b (C16b), right coxal bone. Supplementary Figure S19: Circeo 5 (C5) and Circeo 4 (C4), frontal bone. Supplementary Figure S20: Enamel thickness cartography of Circeo 11(C11), permanent lower right third molar (LRM3), and Circeo 12 (C12), permanent lower left third molar (LLM3). Supplementary Figure S21: Enamel thickness cartography of Circeo 2 (C2), and Circeo 3 (C3), permanent lower right third molar LRM3. Supplementary Table S1: Fossils included in this study for morphological and metric comparative analysis. Supplementary Table S2: Circeo 5 (C5) *calvarium* morphology compared with Lower, Middle, and Upper Pleistocene specimens. Supplementary Table S3: Linear skull measurements. Circeo 4 (frontal bone) and Circeo 5 *calvarium*, individual values compared with other specimens of Pleistocene. Supplementary Table S4: Occipital bone linear measurements. Circeo 8 and Circeo 5 occipital bones, individual values compared with other specimens of Pleistocene. Supplementary Table S5: Frontal bone thickness. Circeo 4 frontal bone and Circeo 5 *calvarium*, individual values compared with other specimens of Pleistocene. Supplementary Table S6: Parietal bone thickness. Circeo 4 parietal bone and Circeo 5 *calvarium*, individual values compared with other specimens. Supplementary Table S7: Frontal bone thickness. Circeo 4 frontal bone and Circeo 5 *calvarium*, individual values compared with European specimens of Pleistocene. Supplementary Table S8: Occipital bone thickness. Circeo 8 and Circeo 5 occipital bones, individual values compared with other specimens of Pleistocene. Supplementary Table S9: Circeo 6 mandible compared with Circeo 2 and Circeo 3 mandibles (original *data*) and other specimens of Pleistocene. Supplementary Table S10: Circeo dental sample compared with other specimens of Pleistocene. Supplementary Table S11: Circeo mandibular M3 dental sample. Three-dimensional enamel thickness and full crown variables assessed in mandibular third molars from Guattari Cave and compared with samples/populations. Supplementary Table S12: Circeo mandibular M3 dental sample 3D taurodontism indices. Supplementary Table S13: Circeo6 (C6) mandible. Ante- and *post-mortem* tooth loss. Supplementary Table S14: Circeo 7 (C7) femur diaphysis—linear diaphyseal diameters. Supplementary Table S15: Cross-sectional geometric parameters of the Circeo 7 (C7) femur and comparison. Distal (20%) femoral diaphysial cross-sectional parameters. Supplementary Table S16: Cross-sectional geometric parameters of the Circeo 7 (C7) femur and comparison. Mid-Distal (35%) femoral diaphysial cross-sectional parameters. Supplementary Table S17: Cross-sectional geometric parameters of the Circeo 7 (C7) femur and comparison. Midshaft (50%) femoral diaphysial cross-sectional parameters. Supplementary Table S18: Cross-sectional geometric parameters of the Circeo 7 (C7) femur and comparison. Mid-proximal (65%) femoral diaphysial cross-sectional parameters. Supplementary Table S19: Cross-sectional geometric parameters of the Circeo 7 (C7) femur and comparison. Proximal (80%) femoral diaphysial cross-sectional parameters [110,111,112,113,114,115,116,117,118,119,120,121,122,123,124,125,126,127,128,129,130,131,132,133,134,135,136,137,138,139,140,141,142,143,144,145,146,147,148,149,150,151,152,153,154,155,156,157,158,159,160,161,162,163,164,165,166,167,168,169,170,171,172,173,174,175,176,177,178,179,180,181,182,183,184].
