# Supplementary material for: Hominin Variability and Evolutionary Relationships at Guattari Cave During the Middle and Late Pleistocene (San Felice Circeo, Latina, Italy)
Source: Genes (Basel). 2026 Jan 26;17(2):132. doi: 10.3390/genes17020132 (PMC12940338; doi:10.3390/genes17020132)
Supplement: Supplementary file 1 [file genes-17-00132-s001.zip › genes-4021948-supplementary.pdf]

Supplementary Information

Hominin variability and evolutionary relationships at Guattari Cave during the Middle and Late Pleistocene (San Felice Circeo, Latina, Italy)

M. Rubini[1,2,3], P. Zaio[1,2,3]*, F. Spanó[4], F. Cognigni[5], M. Rossi[6], A. Gozzi[1], F. Di Mario[1]

1. *Superintendence of Archeology, Fine Arts and Landscape for the Provinces of Frosinone and Latina, Rome, Italy.*

2. *Istituto Italiano di Paleontologia Umana, Piazza Ruggero Bonghi 2, 03012 Anagni, Italy.*

3. *University of Basel, Switzerland.*

4. *Emergency Diagnostics, Policlinico Umberto I, University of Rome "LaSapienza", Italy.*

5. *Carl Zeiss S.p.A, Research Microscopy Solutions, Via Varesina, 162, Milan, 20156, Italy.*

6. *Department of Basic and Applied Sciences for Engineering (SBAI), University of Rome La Sapienza, Via Antonio Scarpa 14, Rome, 00161, Italy.*

* *Corresponding author*

Comprehensive supplementary catalogue of specimens

Descrittive Morphology

Circeo 4 (frontal bone)

In the frontal profile, the medial region is receding and the *sulcus supratoralis* is light and discontinuous as it is interrupted, in the glabellar region, by a median *post-toral* convexity probably accentuated by the pneumatization of the frontal *sinus*. In the frontal and superior views, the *post-toral* convexity could mimic a frontal keel even if a decentred localization with respect to the frontal sagittal line is evident.

Dimensions of the orbits:

The orbits are rounded, and the dimensions of the superior orbital cavity are height ~ 18.2mm and length ~ 33.2mm. The height of the *torus* (highest point 18.7mm) remains more or less unchanged from the medial region (15.8mm) to the lateral one (15.4mm).

Frontal sinuses:

The *supraorbital* arches are incomplete, exposing part of the frontal *sinus.* This appears to be made up of several asymmetrical chambers (at least 6 or 7 can be distinguished) separated by *intrasinus* bony *septa.* Each *sinus* extends superiorly to the medial end of the *supraorbital torus* and back into the orbital portion of the frontal bone. The three right chambers are separated vertically and horizontally oriented bony *septa*. The dimensions (mm) of the three upper, lower and lateral chambers are respectively: 6.6x9.7, 8.5x8.2 and 10.9x14. The fourth middle-central chamber, with dimensions of 32.1x24.3, seems to merge with the left *sinus.* The latter is larger and consists of three chambers separated by septa, of which the central, larger one is separated from the right *sinus* by a *septum* oriented to the right. The dimensions (mm) of the three upper, lower, and lateral chambers are respectively: 11.2x20.8 with a depth of 20.1; 6.4x8.0 and 8.6x18.3. The left lateral recess extends and reaches approximately the middle of the left *supraorbital torus*. The upper part of the left middle-central chamber extends to the *supratoral sulcus* and

its pneumatization determines the extracranial convexity which interrupts the straight path of the *supratoralis sulcus*.
On the internal wall of the middle-central chamber, a *foramen* (4.4x3.4mm) with rounded margins is visible followed by a canal that extends at least for approximately 5.2 mm in depth. The absence of surrounding alterations and a localization near the internal frontal crest associated with vascular morphology do not exclude the existence of a possible relationship with the *Superior Sagittal Sinus* (*SSS*).

Circeo 5 *calvarium*
Frontal Bone:
Dimensions of the orbits:
The orbits are rounded, and the dimensions of the superior orbital cavity are height ~20.7mm and length~ 42.9mm. The height of the torus (highest point ~ 21.2mm) decreases from the medial region (14.7mm) in a lateral direction (11.4mm→10.4mm).
Temporal bone:
The small temporal bone (*squama temporalis*) shows a linear upper profile with a maximum height from *porion* of 37.8mm on the right side and 35.2mm on the left, and a length of ~55.7mm on the right and ~58.5mm on the left. The *squamous* suture descends vertically, at a right angle, to the parietomastoid suture. The zygomatic process extends posteriorly over the *porus acusticus externus*, in a horizontal suprameatal and supramastoid ridge in line with the *torus occipitalis*. Above the root of the left zygomatic process there is a *postglenoid foramen*, indicating the presence of a *petrosquamous sinus* (*PSS*). The mastoid and supramastoid ridges, especially on the right side, appear to be separated by a slight supramastoid groove. The *porus* is oval (width 7.1mm; height 8.9mm and transverse width 10.0mm), oriented almost vertically and is separated from the mastoid process. The tympanomastoid fissure, which separates the tympanic plate from the mastoid process, is absent on the right but may be present on the left. On this side, a slight line of separation between the plate tympanic and the mastoid process is in fact visible, interrupted by a small *post-mortem* damage. The juxtamastoid eminence is incomplete and eroded, and both the juxtamastoid and occipitomastoid crests appear to be absent.
Basicranium:
In the *basicranium* (Figure 13 and Supplementary Figure S7) the mandibular *fossa*, bilaterally is preserved and deep. It has a squamotympanic fissure (STF) that runs into the roof of the *fossa* itself and a coronally oriented tympanic plate [41]. The posterior wall of the TMJ (Temporomandibular Joint) is formed exclusively by the tympanic plate which ends with a thickened and wrinkled crest (possibly the process *supratubalis*) extending, only on the left, up to the *meatus.* The presence of an entoglenoid tubercle is uncertain. The *postglenoid-entoglenoid* length is 22.0 mm. The styloid *foramen* is not alligned with the digastric *sulcus*. The carotid *foramen*, visible only on the right, is posterior to the STF fissure and shows a thickening of the opening margin, of ~2.1mm, and a narrowing of the channel (hole opening diameter 6 mm) which could indicate progressive carotid narrowing (Figure 13a). On the left side, unfortunately, only a small portion of the *foramen* ovale is preserved, preventing evaluation of whether it was double (accessory foramen) or bifurcated. The *sigmoid sinuses* are vertical, and near the left *sigmoid sinus* there is a distinct *sulcus* (Figure S8) which could represent the residue of an atypical drainage pattern of the *intracranial sinuses* (arborizing sigmoid *sinus*).
Occipital bone:
The occipital bone shows ectocranial thickening on the superior nuchal line (underdeveloped nuchal *torus*). The transverse *torus occipitalis* expands laterally in the direction of the *asterion*, most noticeably on the right side. Between the superior and supreme nuchal line there is a small and smooth *suprainiac fossa*, circular in shape. This localization determines a depression of the external occipital protuberance which interrupts the slight *torus* occipital resulting in a double arch morphology. The *inion* is located well above the endinion. A probable *tuberculum linearum* is located near a very poorly developed external occipital crest. The occipitoparietal area is flat and the occipital plane, although presenting a rounded protuberance (swelling) delimited inferiorly by the superior nuchal line, remains in line with the convexity of the nuchal plane. The posterior projection of the *squama occipitalis* (*chignon*) present in C1 skull and more generally in classical Neanderthals is absent in this specimen. The nuchal plane area is incomplete. Sutural synostosis is characterized by interdigitations on the outer table and incomplete obliteration on the inner table. Overall, individual C5 is a young adult female who presents with mild internal frontal hyperostosis.

Circeo 6. Mandible:

***Mandibular foramen*:**

C6 has on the right side (the left side is incomplete) a single oval mandibular *foramen*, horizontal and parallel to the masticatory plane and located under M_1 (P_4). It measures 3.3 x 2.4 mm and is located ~15.3 mm from the alveolar crest (bone loss due to tooth loss) and ~15.5 mm from the basal crest.

Alveolar bone:

The alveolar ridge of the central incisors does not present horizontal atrophy, and the bone level is normal. The presence of a thin fracture line involving only the external alveolar bone is noted in correspondence with the right central and lateral incisors.

After tooth loss, a series of alveolar bone changes (new bone formation and volumetric resorption) occur, resulting in a change in the dimensions and contours of the alveolar ridge [42]. *Data* from one study (meta-analysis) conducted on a modern sample showed an average reduction in buccolingual alveolar ridge thickness of 3.87 mm and vertical mid-buccal resorption of 1.67 mm after unassisted alveolar bone healing [43]. The multifactorial etiopathogenesis of tooth loss does not allow us to determine a specific cause, but for C6, gradual tooth loss due to pyogenic factors (exogenous or endogenous) is excluded, at least as far as the central incisors are concerned. In fact, the alveolar ridge of the central incisors does not show horizontal atrophy, and the bone level is normal. Bacterially induced alveolar bone resorption usually proceeds by a flattening of the alveolar crest (horizontal bone loss) and an enlargement of the bone socket in which the tooth is located (vertical bone loss) [44]. We therefore believe it is probable that the loss of the incisors of the C6 individual can be more easily traced back to a traumatic event which could be confirmed by the presence of a short fracture line in correspondence with the external alveolar bone.

Circeo 8. Occipital bone:

The ectocranial surface morphology of the fossil is well preserved except for some areas of erosion. The endocranial surface is covered with concretions. The occipital plane is convex and low and flattens into the vertical *suprainiac fossa*. The nuchal plane is incomplete. An occipital bun is absent. The "nuchal *torus*", heavily eroded, is an ectocranial thickening located above the superior nuchal line. The *inion* is located immediately above the endinion. The *suprainiac fossa*, located on the superior margin of this thickening, contributes to the determination of its "bilateral arch" morphology. The *torus* has no margins but an upper and lower thinning. The transverse *torus* is thickened medially, lacks significant lateral development. External occipital protuberance is absent. The *suprainiac fossa* is an elliptical depression above the inion which does not extend higher on the occipital plane. Its surface is pocked. In Neanderthals, the *suprainiac fossa* is more pocked in children [45]. The interdigitations of the lambdoid suture, without traces of synostosis, and the pocked surface of the *suprainiac fossa* indicate that this occipital bone could belong to a young individual, therefore not compatible with C4.

Circeo 9. Palatine process of maxilla:

Although the finding shows severe erosive aggression, the presence of a progressive alveolar ridge atrophy due to tooth absence is evident. The *sinuses* sit above the upper teeth. If the upper teeth are missing, the *sinuses* can drop down and invade the space meant to be occupied by the teeth roots. The maxilla in the palatal view (palatine) presents slight traces of *palatine torus* along the margins of the medial palatine suture. There is an asymmetrical thickness between the right and left sides of the palate. Residual dental alveoli are present on the right while the left anterior alveolar ridge shows bone remodeling. Based on the features of the internal nasal region presented by Schwartz et al. [46] we provide a description of the topographic relief of the nasal cavity wall of C9, which preserves part of the nasal *fossae* with the spinal crests and nasal ridges (Figure S10).

***Nasal aperture*:**

The nasal view shows a large piriform opening. Although better delineated on the left both sides bear a probable low posterior nasal crest flow into the base of the spinal ridge. Both sides bear a crisp middle nasal crest that emerges internally to the lateral nasal margin, midway of the nasal aperture, and that fades out before reaching the inferior margin of the nasal aperture. A blunt anterior nasal crest descends up to the region of the anterior spinal-ridge tubercle, absent here. Both sides bear *intranasal* and *subnasal fossae*. Bilaterally, the mediolaterally-thick and superiorly blunt spinal ridges are separated along the midline by a thin groove. The bone behind the posterior nasal crest is mediolaterally-concave and trough-like nasal cavity floor. The anterior most extremities of these ridges, eroded and slightly damaged, no clear evidence of anterior spinal crest spines or tubercles. The dimensions (mm) are: *Nasion-Prosthion*

Height (*n-pr*, NPH) ~15.3, total height 26.2; Maxillo-Alveolar Length (*pr-alv*, MAL) ~ 39.5; Maxillo-Alveolar Breadth (*ecm-ecm*, MAB) ~ 52.6 and 39.8 (*end-end*); Nasal Breadth (*al-al*, NLB) ~ 25.

Circeo7. Right femur (diaphysis):

Circeo7 is without both epiphyses, probably due to endogenous action of hyena. The *maximum* length of the preserved diaphysis is 310 mm. Femoral shaft is strongly curved, platymeric (Index 74.5) in the subtrochanteric part and with weak midshaft pilastric index (79,2mm). The *linea aspera* is continuous and undivided. Its degree of expression is modest but in its proximal portion it is more prominent, probably due to a pilaster consisting of a slight underlying bony crest (Figure S16). Distally the line deviates in a medial direction, becomes smoother, less marked and tends to fade. The areas of muscle insertion appear modest in both the proximal and distal thirds. The cortical distribution pattern of C7 for the 80%, 65% and 50% cross-sections show a constant *maximum* cortical thickness on the medial side and variable from lateral to lateroposterior on the lateral side (Figure S3). Supplementary Tables S4 to S8 show the diaphyseal cross-sectional properties of the proximal, distal, and mid-diaphyseal sections of C7 and the comparative specimens. Supplementary Tables S4-S8 show the diaphyseal cross-sectional properties of the proximal, distal and mid-diaphyseal sections and comparative values.

Circeo 16a. **Coxal bone.**

The partial iliac bone preserves the incomplete iliac wing, a small portion of the auricular surface, a remnant of the tubercle of the iliac crest, a small, preserved margin of the anterior superior iliac spine and the anterior inferior iliac spine. The latter is accentuated by an evident *supra-acetabular sulcus* (and by *post-mortem* damage) which extends between the acetabulospinal buttress and the acetabular rim. Finally, a portion of the *acetabulum* is preserved (*fossa*), with exposure of the spongy bone on the acetabular rim, and a portion of the greater sciatic notch. The portion of the greater sciatic notch present has a height of ~12mm and a width of 30.6mm. The *ischium* and *pubis* are completely missing. In the lateral view, the presence of a single ventral iliac buttress acetabulocrystal (vertical) is noted with a height of ~79mm and 28.9mm distant from the anterior superior iliac spine. The thickness of the buttress varies from a *maximum* of 23.9mm near the acetabular *fossa* to a *minimum* of 15.8mm near the tubercle of the iliac crest. Its central thickness is 19.5mm. In medial view there is a well-defined arcuate line and a thick iliosciatic buttress (24.4mm). The height from the acetabular *fossa* to the iliac crest is ~115.4mm.

Circeo 16b. Coxal bone:

The partial iliac bone preserves the incomplete iliac wing, a portion of the auricular surface, a small remnant of the iliac crest (tubercle iliac crest?), a small preserved margin of the anterior inferior iliac spine, the latter accentuated by an evident supra-acetabular *sulcus* which extends between the acetabulospinal buttress and the acetabular rim, a portion of the acetabular *fossa* with exposure of the spongy bone on the acetabular rim and finally a portion of the greater sciatic notch, preserved for a height of ~22.3mm and a width of 39.5mm. The *ischium* and *pubis* are completely missing. In the lateral view, the presence of a single ventral iliac acetabulospinal buttress with a height of ~42.6mm is noted (concretions are present). The thickness of the buttress varies from a maximum of 25.0mm near the acetabular fossa to a minimum of 17.0mm. Its central thickness is 21.0mm. There is a thick iliosciatic buttress (23.1mm). The height from the acetabular *fossa* to the iliac crest is ~106mm. Given the small value of this height, a link with C5 cannot be excluded.

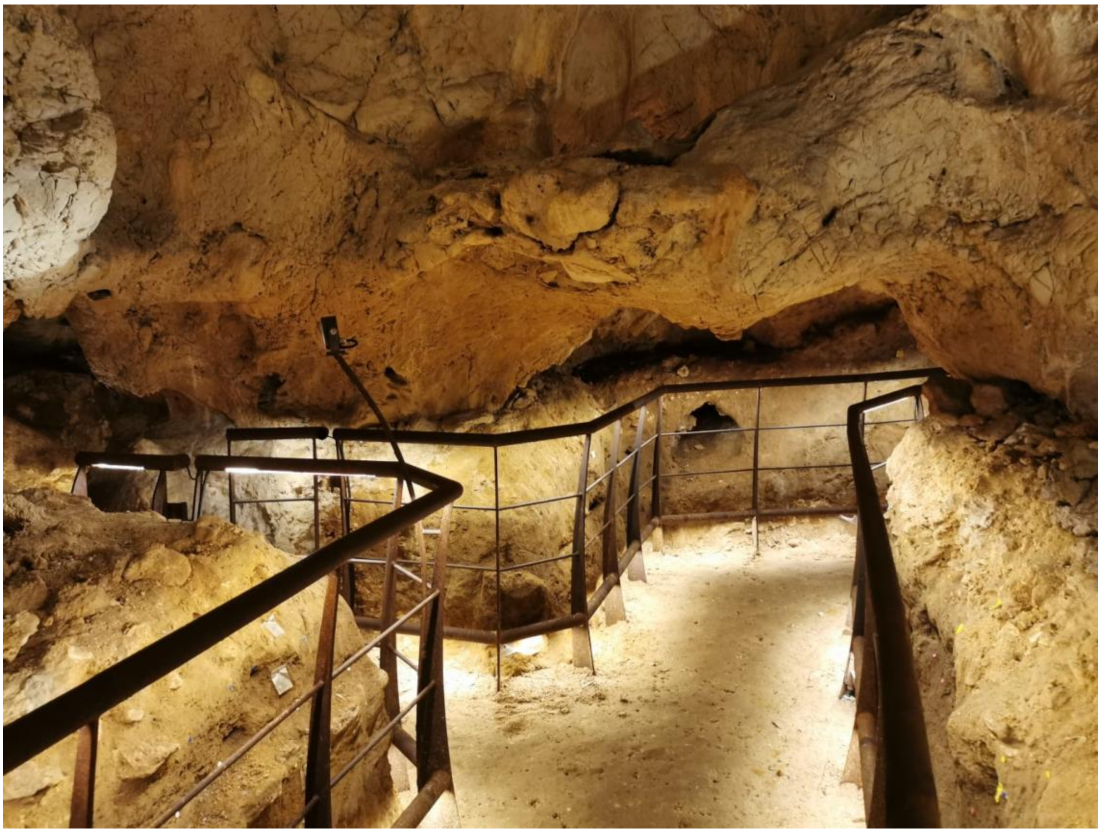

Figure S1. Internal path of the Cave

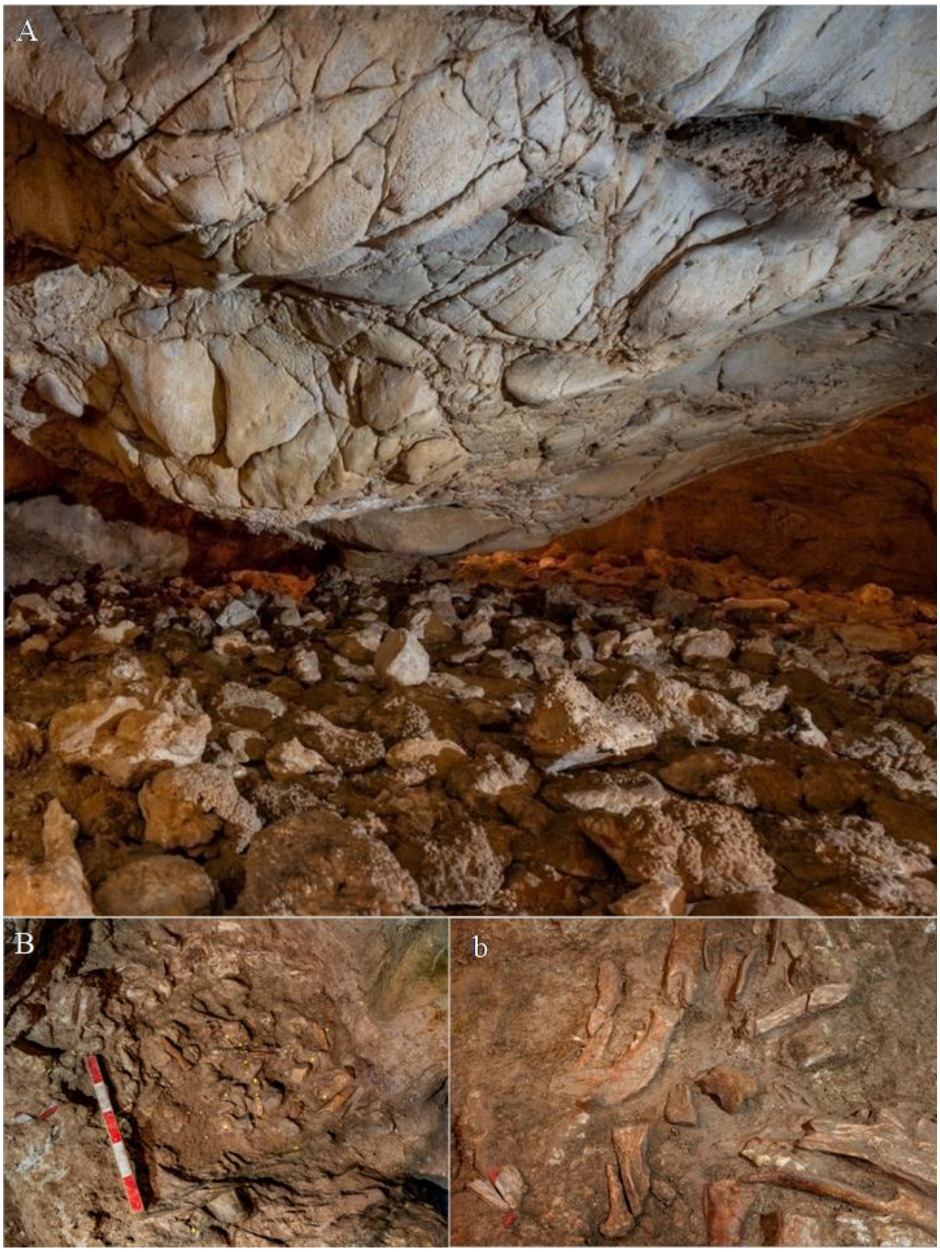

Figure S2. A, Interior of the Cave and B, b, excavation area.
(Photo credit Mauro Rubini and Paolo Petrignani)

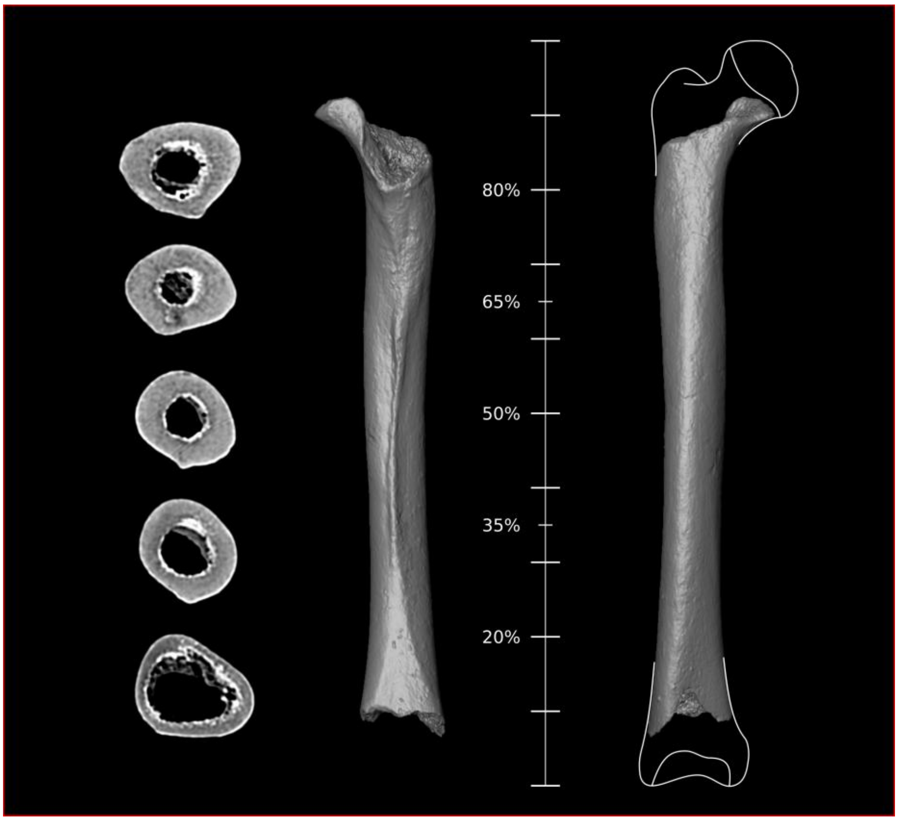

Figure S3. 3D reconstruction Circeo 7 (C7) femur. Entire diaphysis, approximate total length, and individual cross-sections. No evidence of a centro-diaphyseal trabecularization process C7 is a probable young individual.

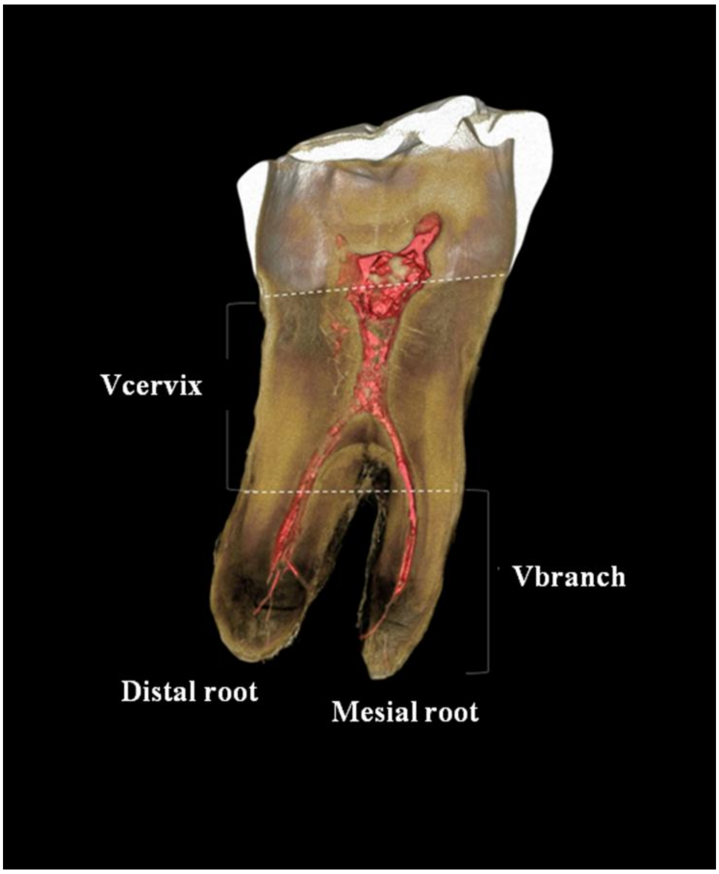

Figure S4. Circeo12 Lower Left third molar (LLM3). VBI volumetric bifurcation index in % [= Vcervix/ (Vcervix +Vbranch) ×100].

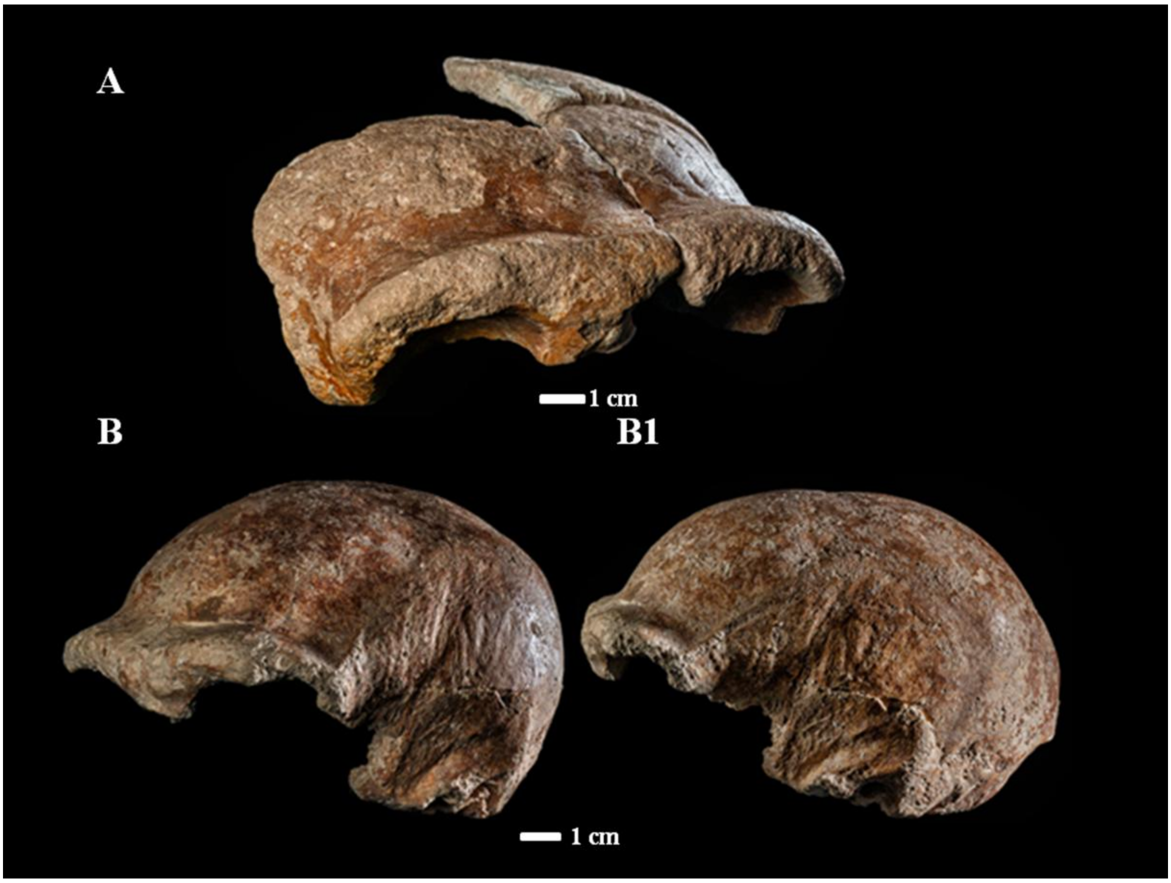

Figure S5. Circeo 4, (**A**), **Circeo 5** (**B, B1**). (Photo credit Mauro Rubini and Paolo Petrignani).

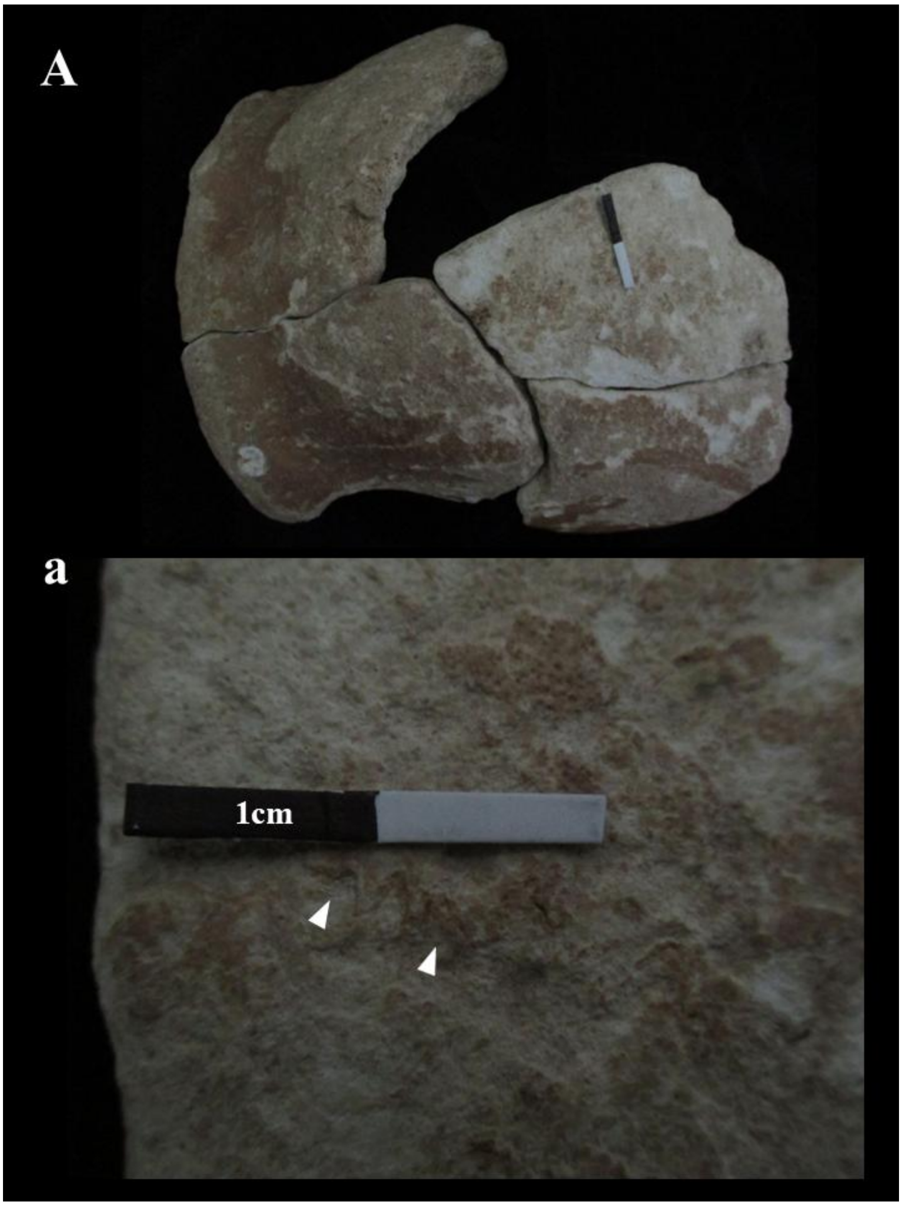

Figure S6. Circeo 4. A, superior view; **a**, coronal suture.

.

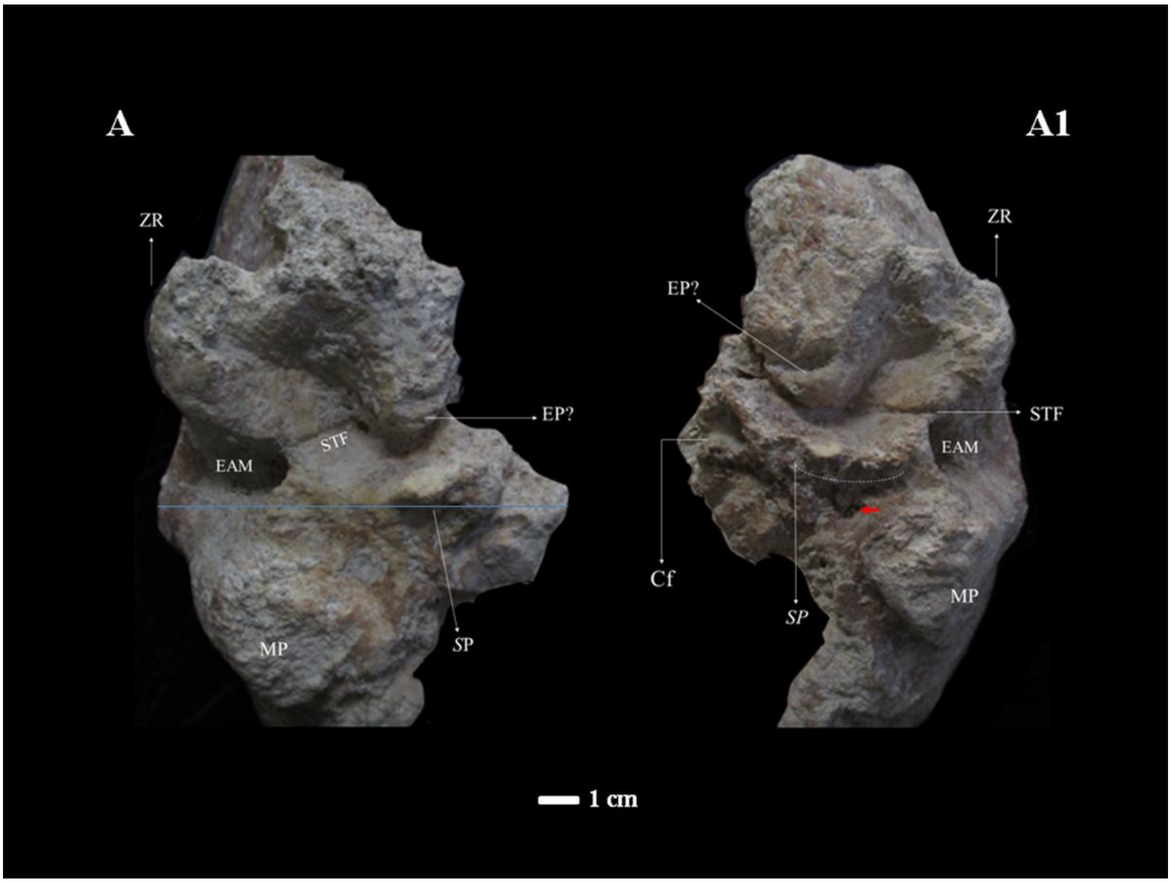

Figure S7. Circeo 5 *basicranium, asymmetry of the supratubalis process*. Temporal bone right (**A**) and left (**A1**) mandibular *fossa* (TMJ) viewed in *norma basalis*. **red arrow**, *styloid foramen* (on the right it is not visible because it is closed by concretions); ***S*P**, *Supratubalis* process (only on the left does it extend to the External *Acoustic Meatus* **EAM**); **STF**, Squamotympanic fissure; **Cf** Carotid *foramen*; **MP**, Mastoid Process; **ZR**, Zygomatic Root; **PP**, Preglenoid (entoglenoid) Process (?). Note (In A and B) the absence of the postglenoid process, the styloid process, the vaginal process and the position of the squamotympanic fissure (**STF**) that runs into the roof of the fossa itself and coronally oriented tympanic plate.

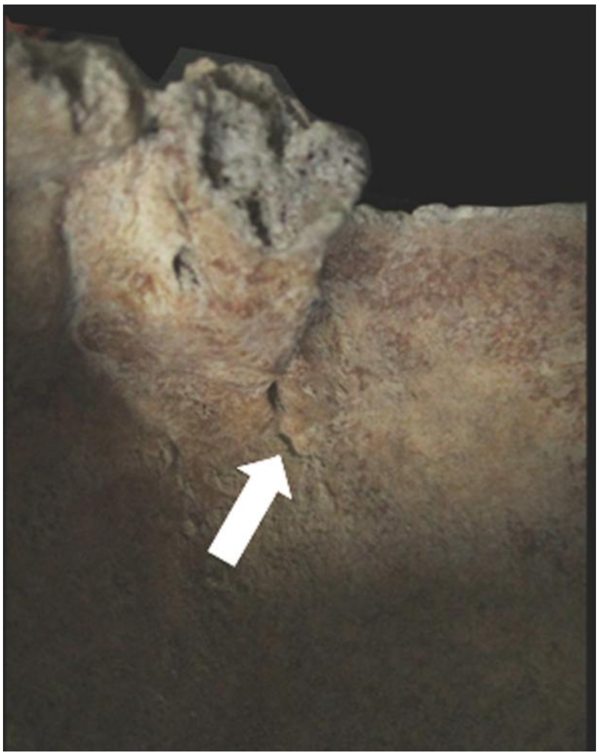

Figure S8. Circeo 5, temporal bone left petrous part. A distinct groove (white arrow) that diverges from the *sinus sigmoid* could represent the residue of an atypical intracranial *sinus* drainage pattern (arborizing *sigmoid sinus*).

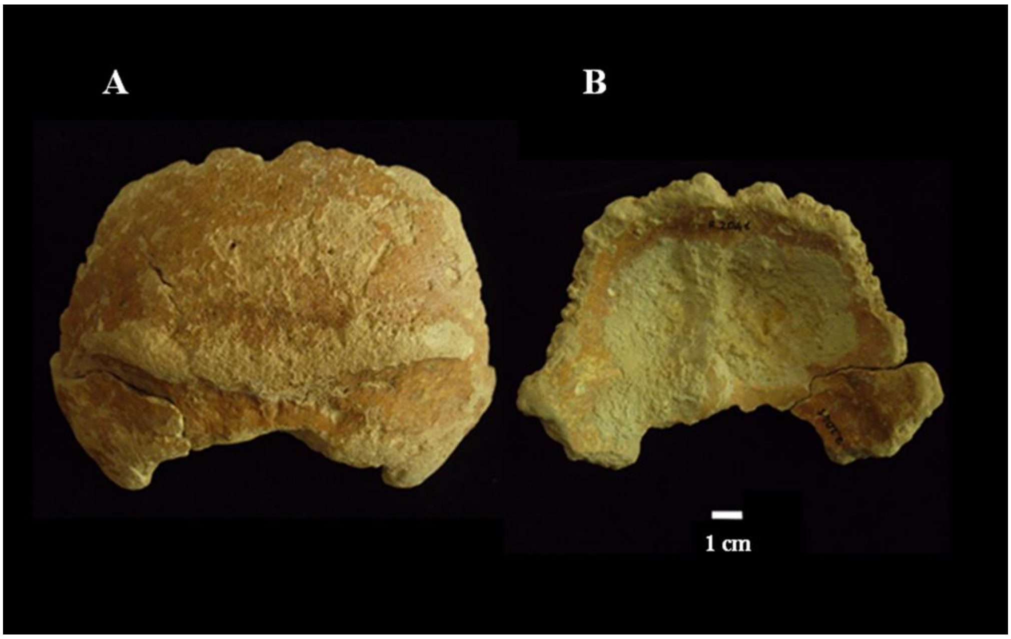

Figure S9. Circeo 8, occipital bone *(squama occipitalis)*. View: **A**, ectocranial; **B**, endocranial.

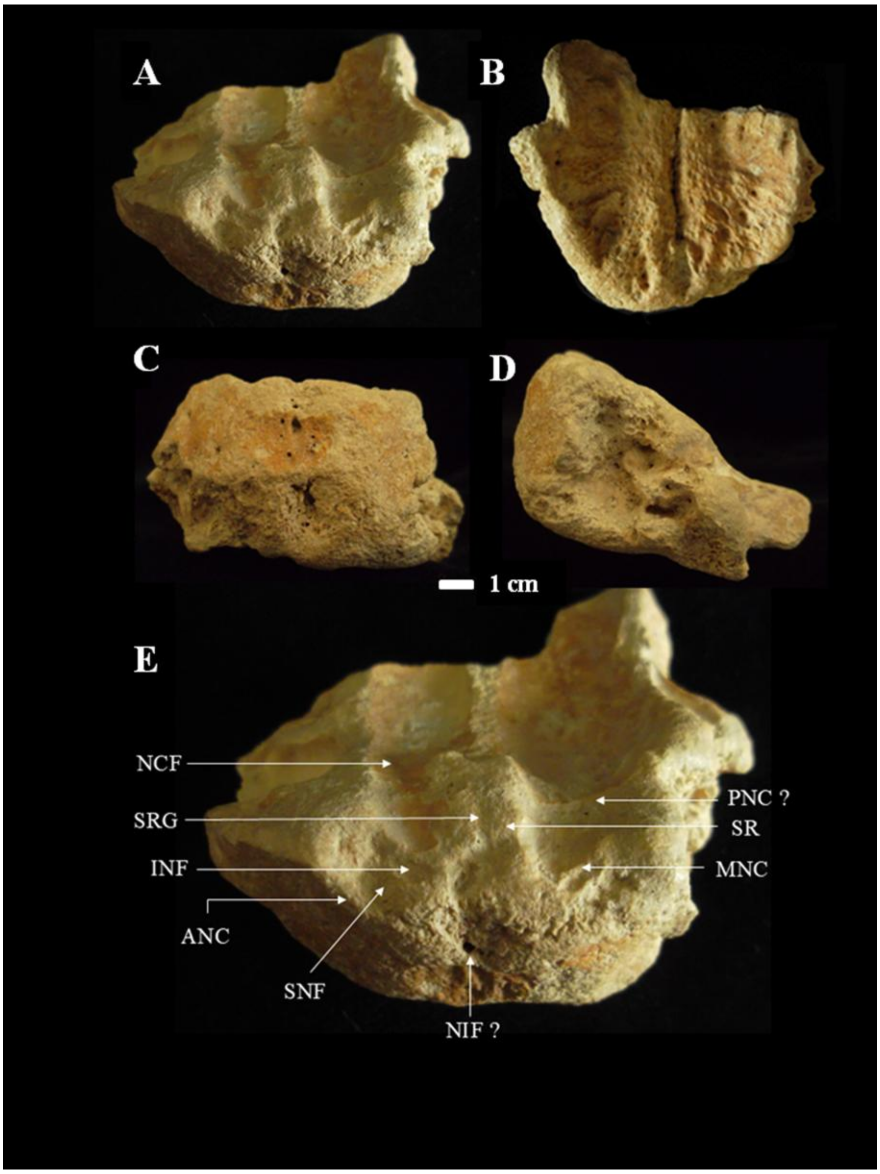

Figure S10. Circeo 9, palatine process of maxilla. Maxilla: superior (**A**), inferior (**B**), anterior (**C**) and left lateral (**D**) views. **E**, superior-view closeup and illustration of features identified: **SNF**, Subnasal Fossa; **ANC**: Anterior Nasal Crest; **INF**, Intranasal Fossa; **SRG**, Spinal-Ridge Groove; **NCF**, nasal cavity floor; **PNC**, Posterior Nasal Crest (?); **SR**, Spinal Ridge; **MNC**, Middle Nasal Crest; **NIF**, Naso-Incisive *Foramen* (?).

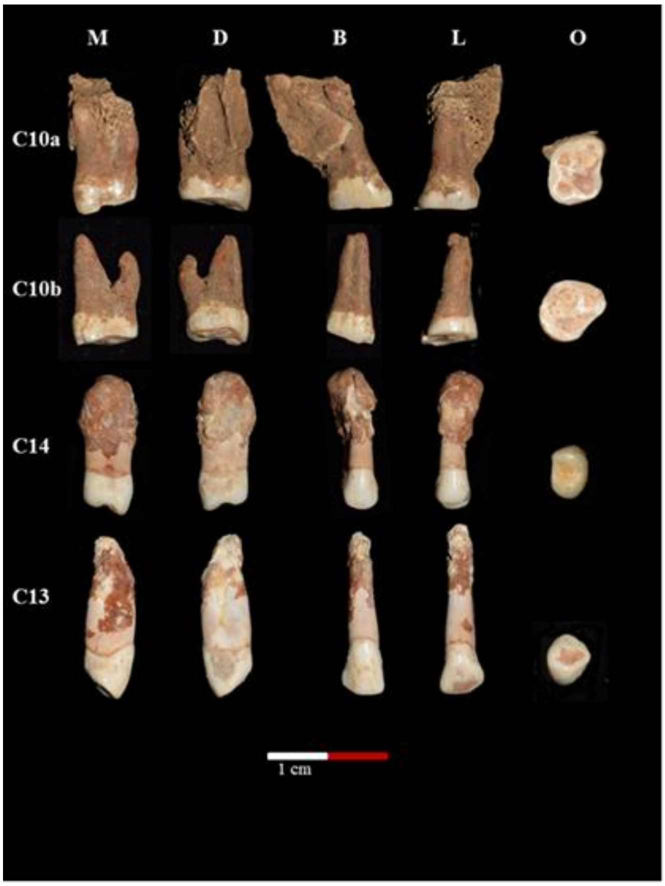

Figure S11. Upper teeth. View: M**,** mesial; **D,** distal; **B**, buccal; **L,** lingual; **O,** occlusal. **C**, Circeo

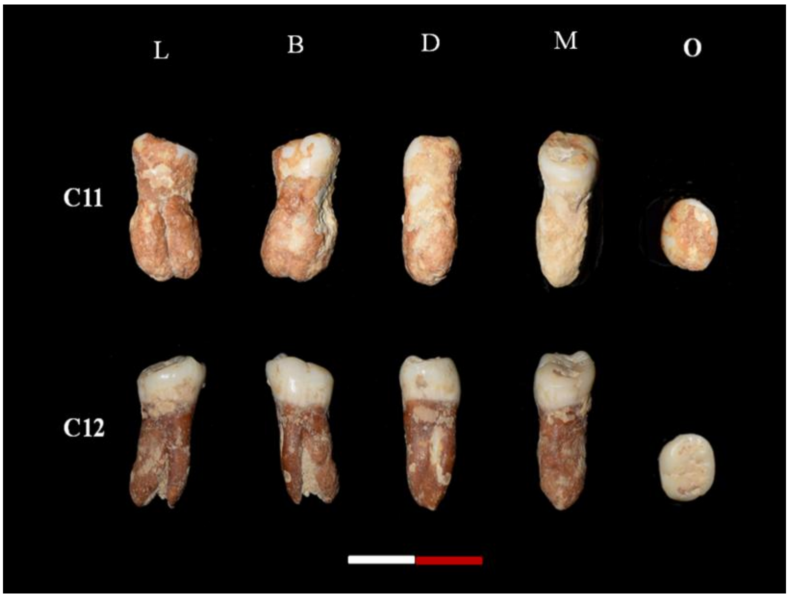

Figure S12. Lower teeth (mandibular). View: **L**, Lingual; **B**, Buccal; **D**, Distal; **M**, Mesial; **O**, occlusal. **C**, Circeo.

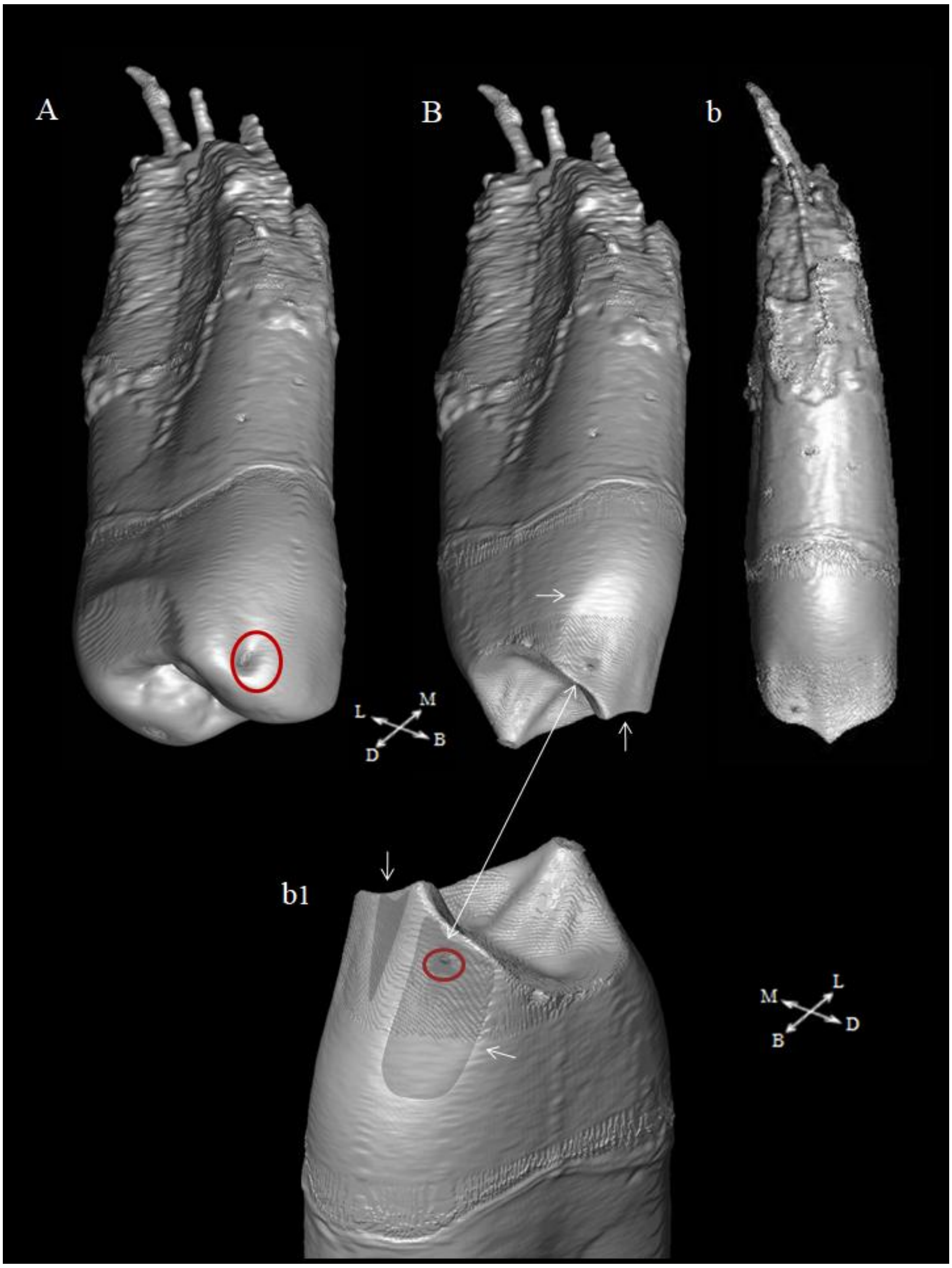

Figure S13. Circeo 14 upper right second premolar (URP4). 3D reconstruction of its external surface **A**, Anterior-distal view at the outer-enamel surface (vestibular)and **B**, enamel-dentine junction (enamel digitally removed). **b**, buccal (vestibular) view enamel-dentine junction. On the distal vestibular surface of the crown there is an archaic character from the Middle and Upper Pleistocene consisting of a buccal vertical groove (red circle). The vertical groove is associated with a clear concavity at the enamel-dentine junction (white arrows and white double arrow) and a weak and indistinct concavity is also present on the mesial aspect associated with a small vertical ridge (both highlighted in **b1**). Note the distal contact facet in **A**.

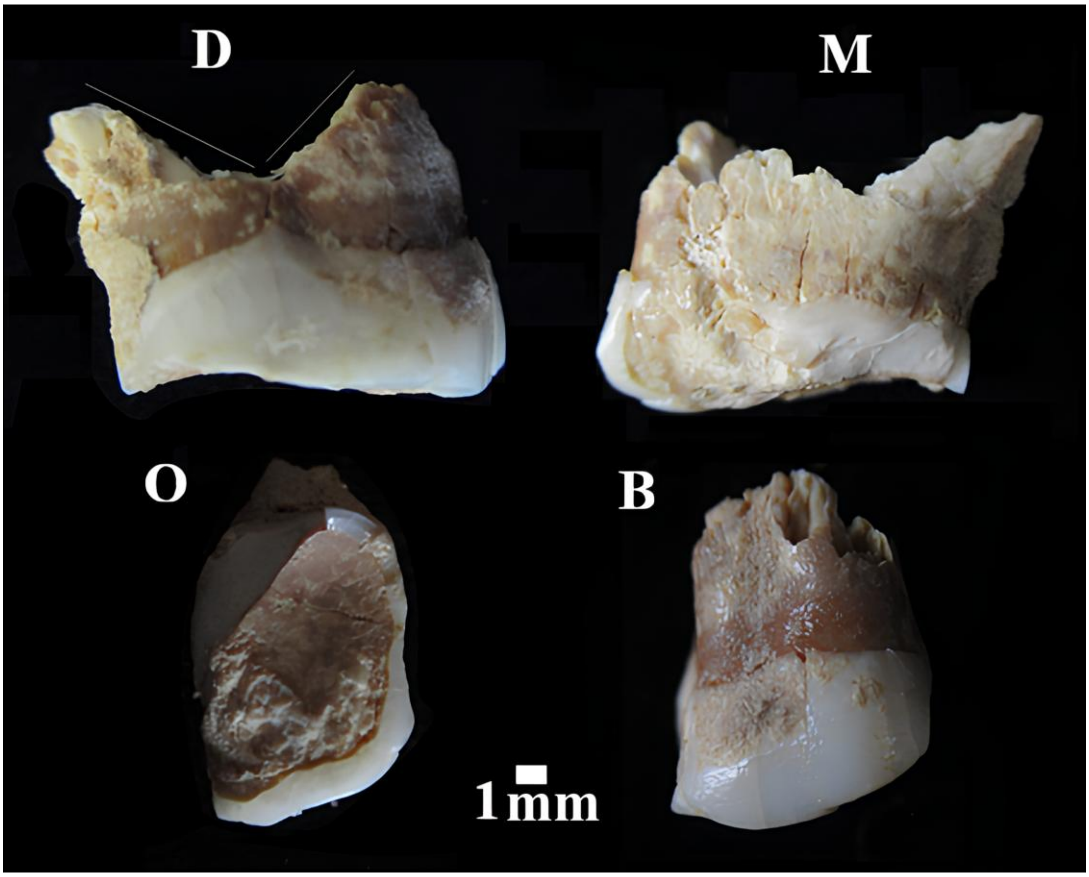

Figure S14 . Circeo 15, permanent upper right first molar (M^1). The crown, incomplete, shows a mesiodistally compressed morphology and the roots, incomplete, are small and strongly splayed. Abbreviations: **D**, Distal; **M**, Mesial, **O**, Occlusal, **B**, Buccal.

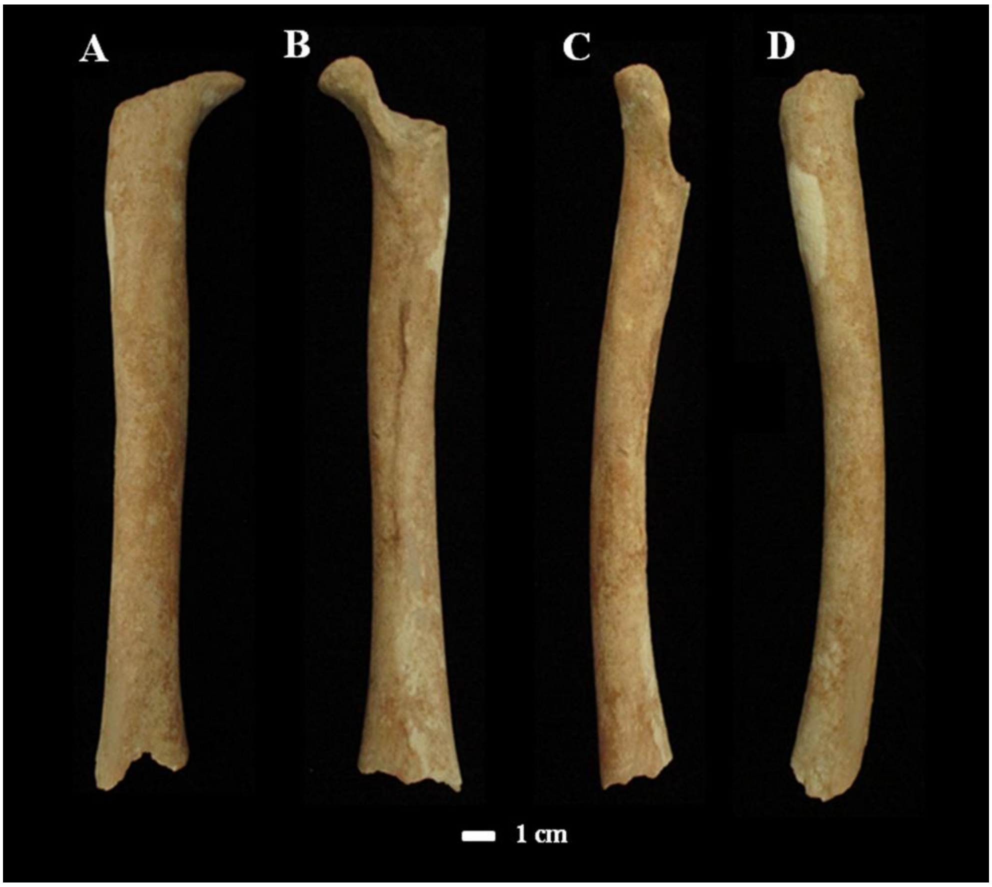

Figure S15. Circeo 7, right femur (diaphysis). View: **A**, anterior; **B**, posterior; **C**, medial; **D**, lateral.

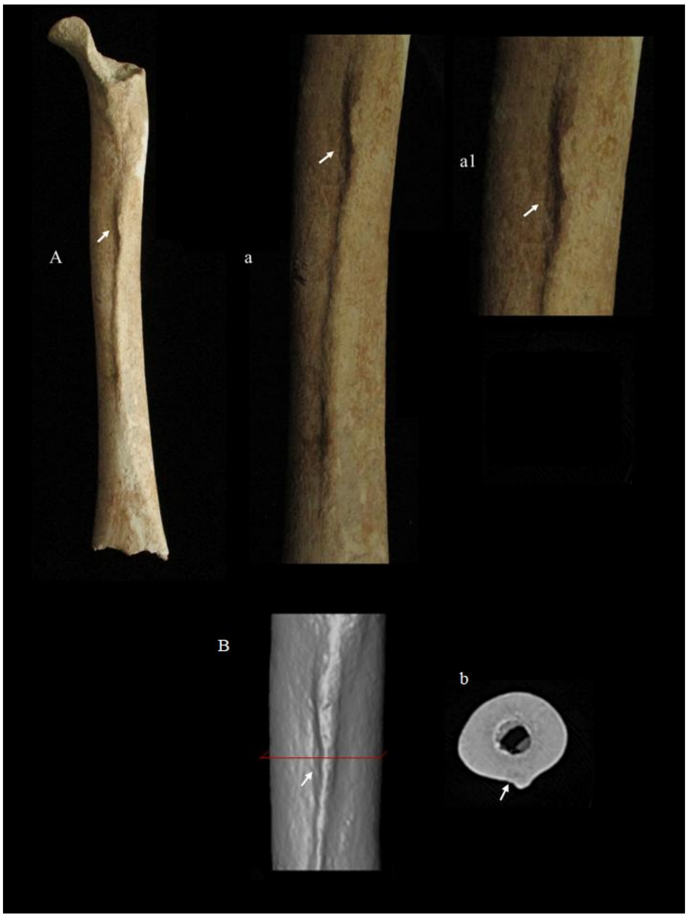

Figure S16. Circeo 7 right femur (diaphysis). A, a, a1 femur posterior view. **B**, 3D reconstruction of mid-diaphysis femur and **b**, cross-section. Probable pilaster white arrows. Not to scale

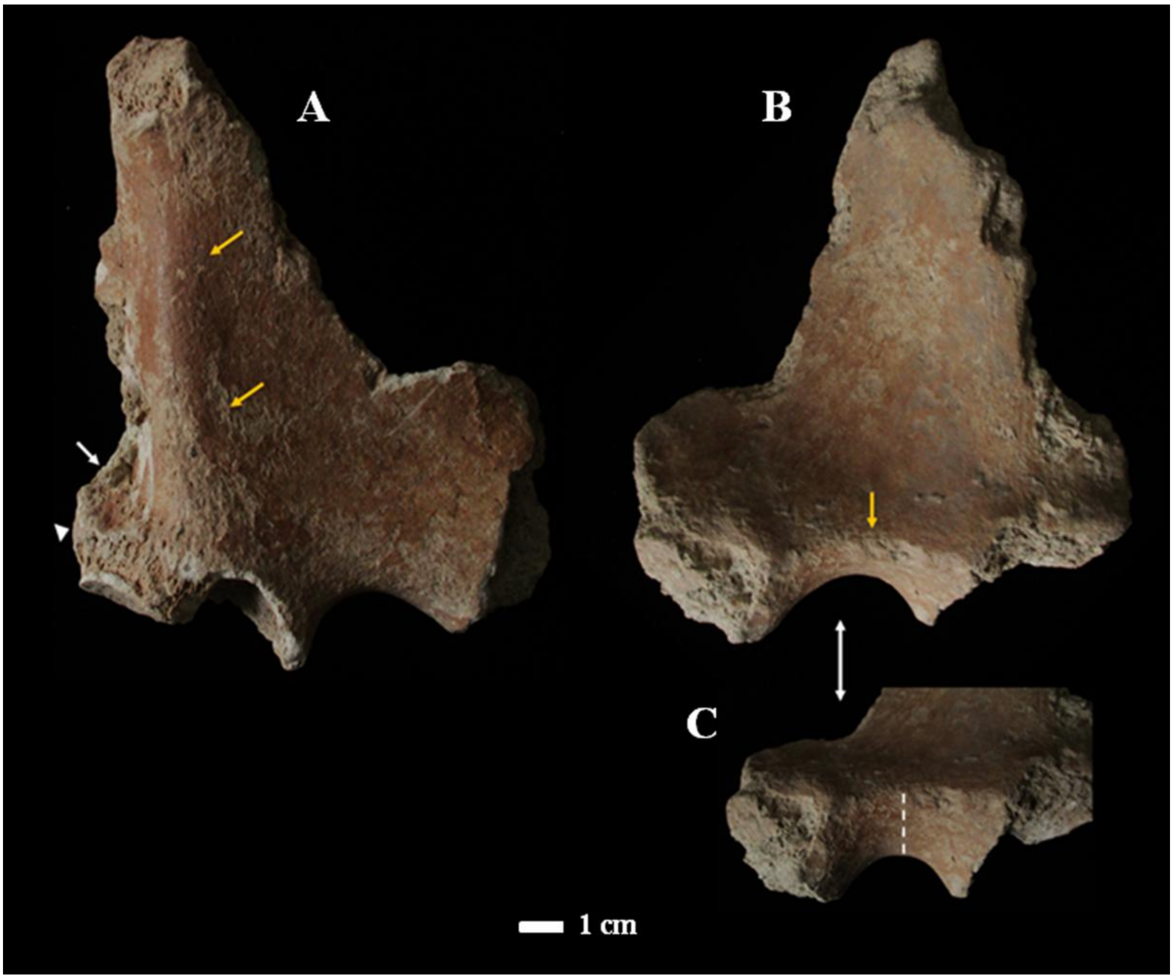

Figure S17. Circeo 16a, left coxal bone. **A**, medial view, single ventral iliac buttress acetabulocrystal (vertical) [orange arrows]; supra-acetabular sulcus (accentuated by damage post- mortem) [white arrow]; anterior inferior iliac spine [white arrowhead]. **B**, lateral view, well-defined arcuate line (orange arrow) and thick iliosciatic buttress (**C**) [white dotted line].

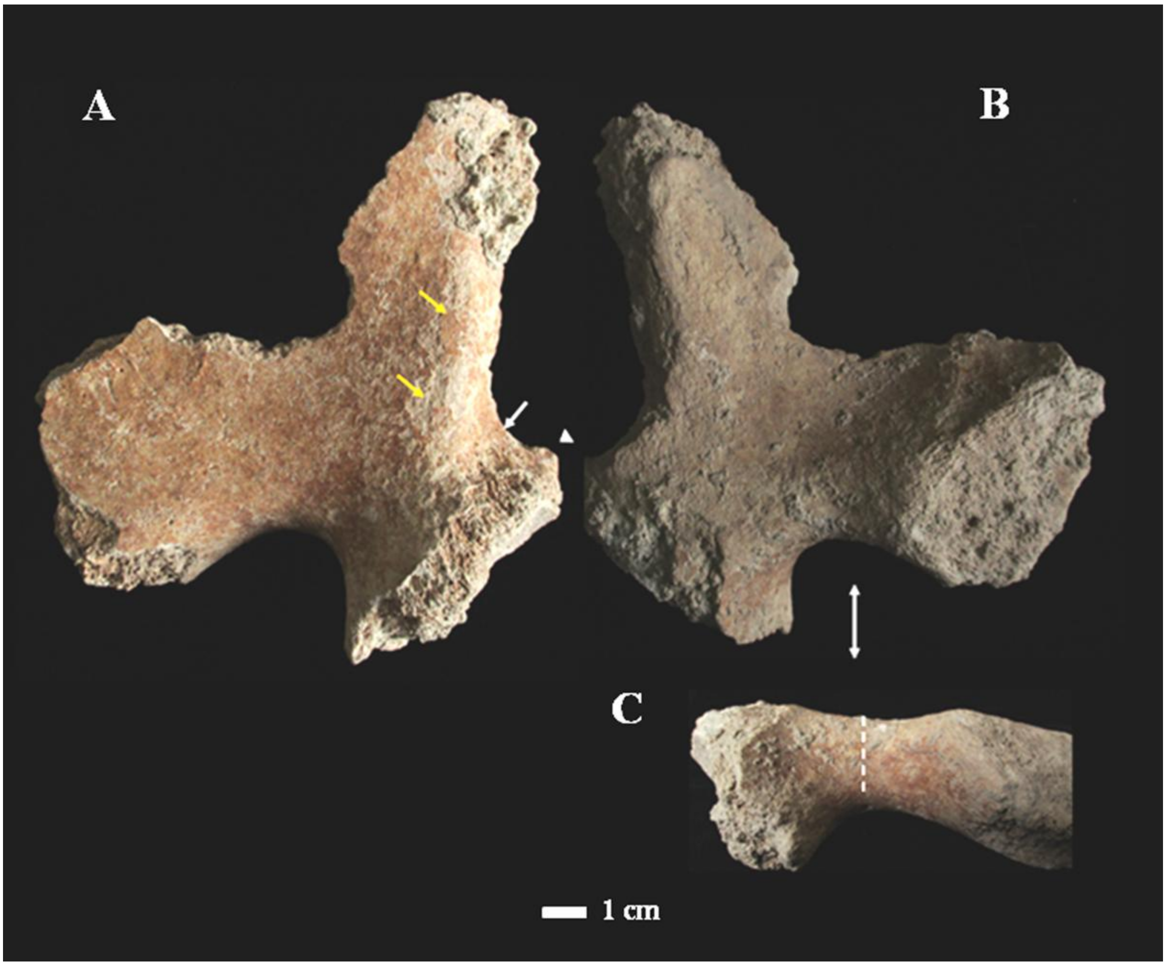

Figure S18. Circeo 16b, right coxal bone. **A**, medial view, single ventral iliac acetabulospinal buttress (orange arrows); supra-acetabular sulcus (white arrow); anterior inferior iliac spine [white arrowhead]. **B**, lateral view, thick iliosciatic buttress (**C**) [white dotted line].

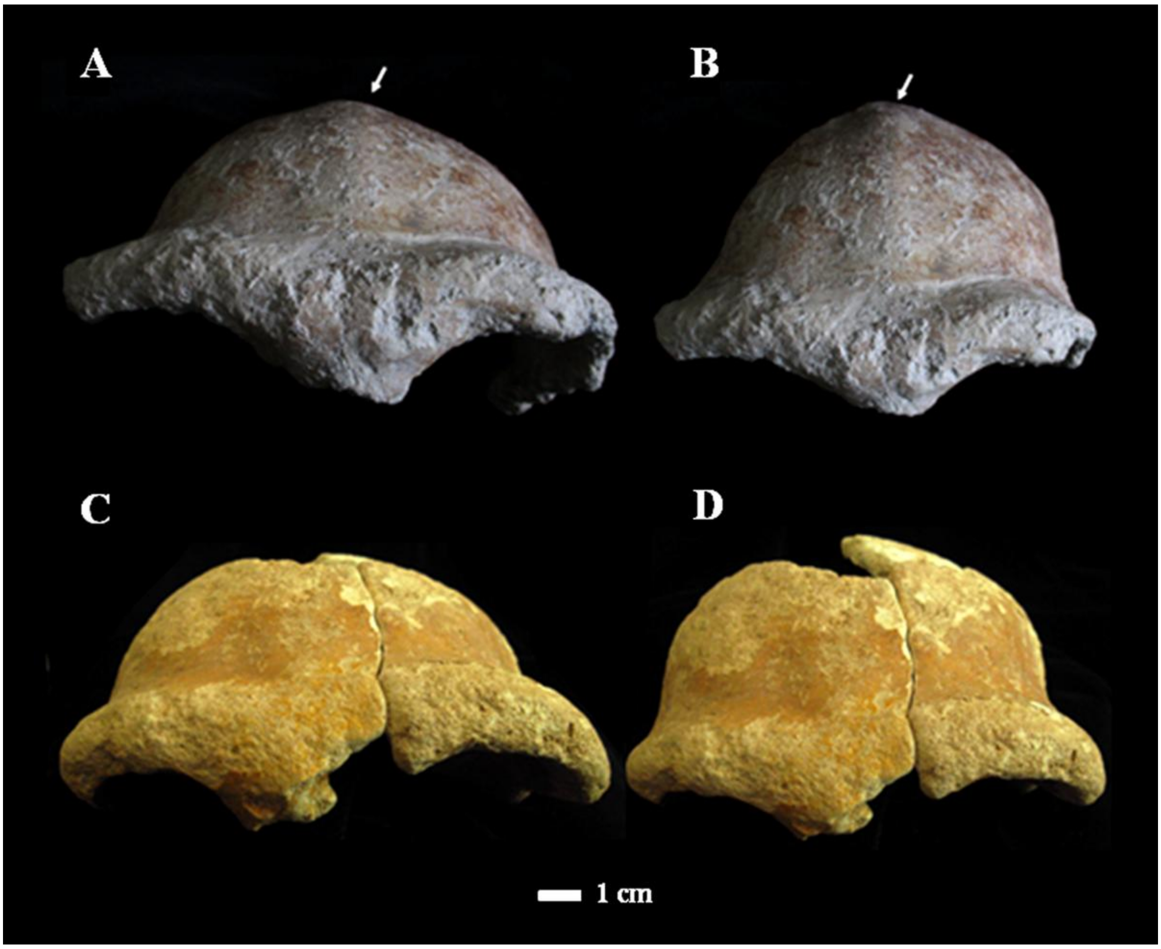

Figure S19. Circeo5(A, B) and Circeo4 (C, D), frontal bone. View: Anterior (A, C) and anterior-superior (B, D).

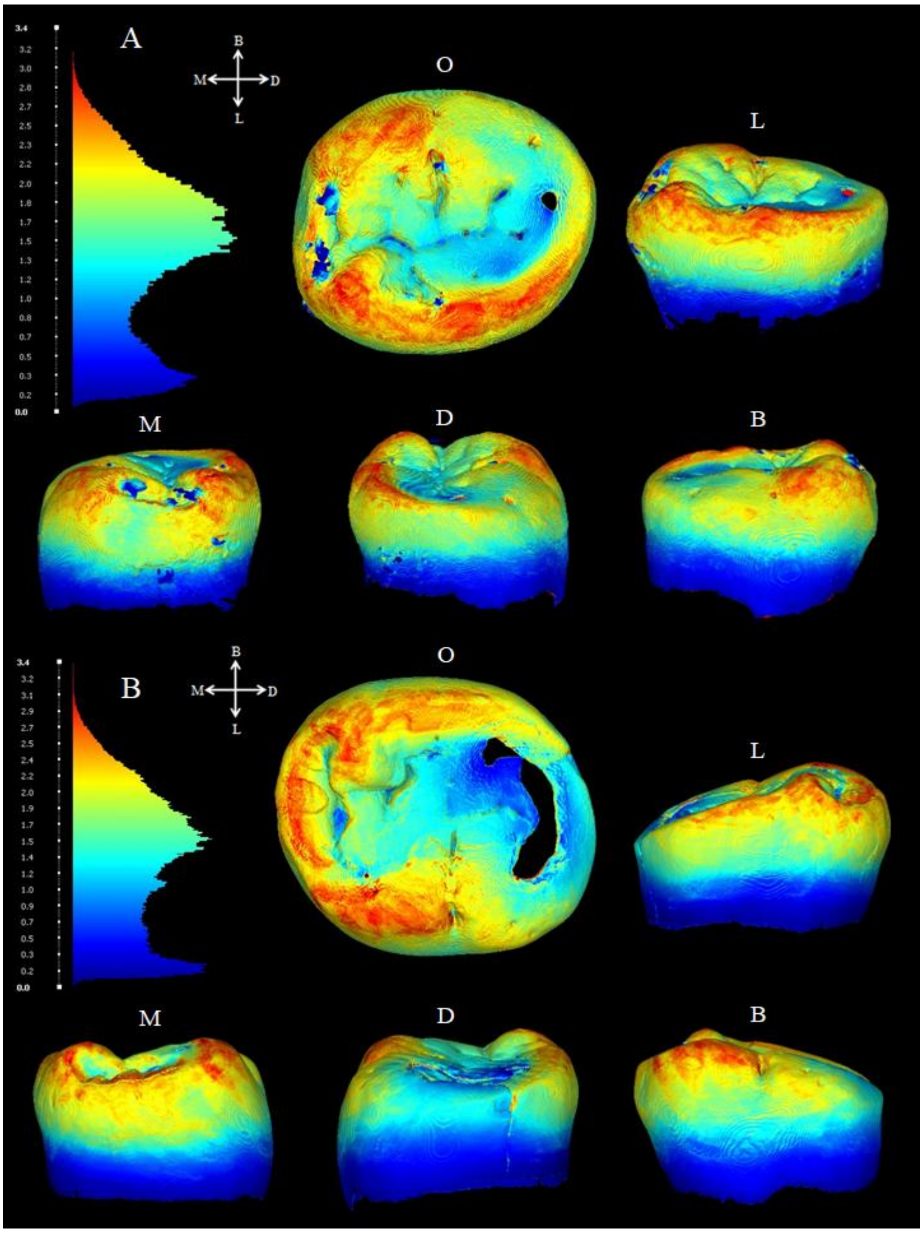

Figure S20. Enamel thickness cartography of: **A**, Circeo11(C11) LRM3 and **B**, Circeo 12 (C12) LLM3. Topographic thickness variation is rendered by a pseudo-color scale ranging from thinner blue to thicker red. Abbreviations: **O**, occlusal, **M**, Mesial; **D**, Distal; **B**, Buccal; **L**, Lingual; **LRM3**, lower right third molar; **LLM3**, lower left third molar. Not to Scale.

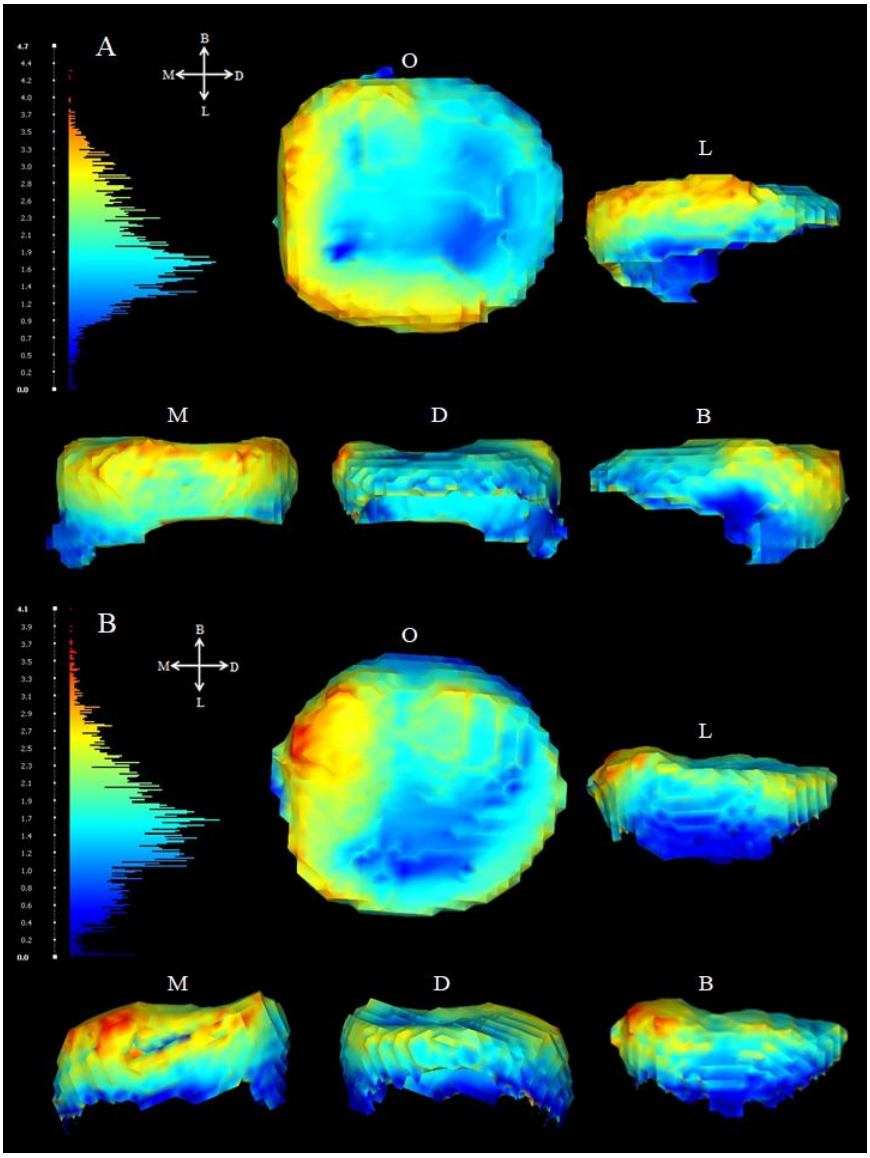

Figure S21. Enamel thickness cartography of: **A,** Circeo 2 (C2) LRM3 and **B,** Circeo 3 (C3) LRM3. Topographic thickness variation is rendered by a pseudo-color scale ranging from thinner blue to thicker red. Abbreviations: **O**, occlusal, **M**, Mesial; **D**, Distal; **B**, Buccal; **L,** Lingual; **LRM3**, lower right third molar. Not to Scale.

Table S1. Fossils included in this study for morphological and metric comparative analysis.

Samples included in cranial comparison	
**Sample/specimens (*Genus*)**	**Date source**
**Proto-Neanderthal**	
Arago 47	[110]
Arago 21	[70, 50, 49]
Petralona 1	[70, 49, 110, 111]
Sima de los Huesos: SH 1, 2, 3, 4 (+AT 600), 5 (+AT 700), 6, 8 (+AT 433); Occipital: II, IV	[49, 110]
Bilzingsleben	[110, 111]
Reilingen (occipital)	[110]
Steinheim	[110, 111]
Swanscombe 1	[110, 111]
**Neanderthal**	
Neanderthals group	
(Early, MIS 7-5 and Late, MIS 5d-2 [109])	
Ehringsdorf 1, 2	[111]
Kůlna (Moravia)	[111]
Saccopastore I	[112, 113, 51]
Apidima 1 (occipital), 2	[110]
Biache-Saint-Vaast 1, 2	[51, 50, 110]
Krapina: 3; 5; C; D; E; 6 (+Occ.6); 8; 10; 11; 16; 18.1; 18.2; 18.5; 18.6; 18.9; 20; 21; 32 (32,1?); 34.1; Krapina 2 (Jouvenile 10-12y)	[113, 51, 114, 115, 110, 111]
La Chaise Suard: S1 (S1+S17), S9, 16	[113, 110]
La Chaise Bourgeois-Delaunay: BD6, BD17 a, b	[113, 110]
Šalá (Slovak Republic)	[110]
Gibraltar 1	[111, 113, 51, 112,110]
Amud 1	[116, 111, 51, 112, 110]
El Sidrón: SD-1149, SD-1219 (Occipital)	[113, 110]
Fontéchevade II	[110]
Tabun 1	[111, 113, 114, 112]
Spy: 1, 2	[111, 113, 114, 112, 110]
Vindija: 204, 205, 252,258,261, 281,282, 293	[110, 111]
La Pélénos	[110]
Petit-Puymoyen PPm7 occipital (Adolescent)	[110]
La Chapelle aux Saints 1	[111, 113, 114, 111, 70, 110]
La Ferrassie: 1, 3 (Sub-adult)	[111, 113, 114, 112, 70, 110]
La Quina: H5, 13	[111, 113, 50, 110]
Shanidar 1, 2, 4, 5	[111, 112]
Circeo 1	[111, 117, 113, 112]
Feldhofer (Neanderthal 1)	[111, 112, 113, 114, 51, 110]
Salzgitter-Lebenstedt 1	[113, 114, 110]
Le Moustier	[111, 112]
Carihuela	[50]
H. habilis	
KNM-ER 1813	[1, 70]
KNM-ER 3883	[47, 118, 1, 70]

Samples included in cranial comparison	
**Sample/specimens (*Genus*)**	**Date source**
KNM-WT 15000	[47, 118]
SK 847	[47]
H. rudolfensis	
KNM-ER 1470	[1, 70]
Buia	[47]
H. heidelbergensis	
Broken Hill (Kabwe)	[1, 70, 119, 111]
Bodo 1	[111]
Ndutu (Steinheim)	[111]
Florisbad	[111]
Ternifine (Algeria)	[111]
**Ceprano**	**Original data**
H. ergaster	
Melka Kunture	[111]
KNM-ER 3733	[47, 118, 1, 70]
Olduvai Hominid: OH 9, OH 12	[111, 47, 1, 70]
Daka-Bouri (VP2/66)	[1, 70]
H. erectus	
Kocabaş (Denizli-Turkey)	[70]
Dmanisi: 3444, 2282, 2280, 2700, 4500	[47, 118, 1, 70, 110]
Lazaret 24 (Late *H. erectus*)	[50]
Sambungmacan: 1,3, 4 (*H. erectus* Middle Pleistocene SM3 0.5 Ka)	[120, 121, 47, 1, 70]
Sangiran: 2, 3, 4, 9, 10, 12, 13, 17, 18, 27 (S17 0.8 Ma)	[111, 121, 47, 118, 1, 70, 110]
Bukuran (Early Pleistocene of Java)	[1]
Nanjing 1 (~ 0.58-0.62 Ma / ~580-620 Ka)	[47]
Trinil 2	[111, 121, 47]
Lantian	[111]
Yunxian II (China) [936 Ka]; Nankin 1 (Hulu cave, China) [641 ± 39 Ka]	[70]
Zhoukoudian: 1, 2, 3, 4, 5, 6,10,11, 12 (ZKD 3-11-12 780 ± 80 Ka)	[111, 47, 1, 70, 110]
Ngandong: 1,3,5,6,7,9, 10, 11, 12 (Late Pleistocene)	[111, 121, 47, 122,1, 70]
Ngawi 1 (late *H. erectus* Middle–Upper Pleistocene)	[121, 1]
Hexian (PA831) (412 ± 25 Ka or roughly contemporaneous with the Zhoukoudian [ZKD] specimens)	[111, 1]
Dali (270 Ka)	[111, 1, 119]
Harbin (minimum uranium-series age of 146 ka)	[119]
Salè (Morocco)[250-200 Ka]	[111]
**Pleistocene/Holocene**	
H. sapiens	
Xuchang 1; Xujiayao: 4, 5, 6, 9, 10,11; Maba (China)	[119, 111]
Wadjak 1 (Indonesia)	[111]
WLH 50 (Australian)	[122]
Mungo 1,3; Kow Swamp 16; Keilor; Tandou 2 (Australian)	[111]
Australian Pleistocene/Holocene	[123, 111]

Samples included in cranial comparison	
**Sample/specimens (*Genus*)**	**Date source**
Salkhit (Mongolia)	[124]
Jebel Irhoud: Irhoud 1 (Morocco North African Late Middle Pleistocene)	[111, 88, 119, 110]
Skhul (Middle East, Israel): 2, 4, 5, 6, 9	[111, 125, 51, 110]
Qafzeh (Middle East, Israel): 3, 5, 6, 7, 9	[111, 51, 114, 110]
Manot 1 (Middle East, Israel)	[52, 88]
Mladeč (Czech Republic): 1, 5, 6	[114, 88, 110, 111]
Dolní Věstonice (Czechia Republic): 3, 13, 14, 15, 16	[110]
Pavlov 1 (Czechia Republic)	[110]
Předmosti (Czechia Republic): 1, 3, 4, 9, 10, 13, 14	[110, 111]
Cotte de St. Brelade (Jersey)	[111
Cro-Magnon (France): 1,2,3	[114, 111]
Fontechevade 5 (France)	[111]
Oberkassel1,2 (Germany)	[111]
Boskop; KRM 41658 (South Africa)	[111]
Lukenya (Kenya)	[111]
Laetoli 18 (Tanzania)	[110]
Omo 1,2 (Ethiopia)	[111]
**Holocene**	
French, Yuendumu; Africans; Chinese; Phillipines; Hebrides; American white; Belgian; Byblos; Sialk Copper Age; Sialk Iron Age; Hastière; French Neolithic; Susa; Palmyra	[111]

Samples included in mandibular morphological and metric comparison	
**Sample/specimens**	**Date source**
**Africa**	
**African archaic Middle Pleistocene:**	[126]
KNM-BK 67 (Baringo-Kapthurin 67), KNM-BK 8518 (Baringo-Kapthurin 8518), Sidi Abderrahman 2, Thomas 1 (Quarry I), Thomas Gh 10717 (Quarry I), Tighenif 1,2,3	
**Pliocene and Early Pleistocene** (*Australopithecus*):	[127]
Hadar, Laetoli, Makapansgat, Sterkfontein, Malapa	
**Early Pleistocene** (Early *Homo*):	[127]
Koobi Fora, Olduvai, Sterkfontein	
**Early Pleistocene** (*H. ergaster or H. erectus*):	[127, 128]
Koobi Fora, Olduvai, West Turkana, Swartkrans, KNM-ER: 730, 731, 819, 992, 1506, 1808, 3950: OH 22, 23, 51, KGA10-1	
**North Africa Middle Pleistocene** (*H. erectus*):	[127]
Tighenif, Rabat, Thomas Quarry, Sidi Abderrhaman	[128]
KNM-BK 67, 8518 (East Africa)	
Tigehnif 1, 2, 3; Thomas Quarry	

Samples included in mandibular morphological and metric comparison	
**Sample/specimens**	**Date source**
**East Asia**	
Hexian (PA831)	[127]
**Javanese *H. erectus***	
**Early Pleistocene from Java:**	[127, 128]
Pucangan/Sangiran, Sangiran,grenzbank, Bapang-AG, Sangiran 1b, 5, 6a, 8, 9, 22, Bk7905, Bk 8606	
Sangiran 9 and 22	[129]
**Chinese *H. erectus***	
**Early MP *Homo* From Zhoukoudian and Lantian:**	[128]
Lantian, Zhoukoudian PA86, Zhoukoudian: A1, G1, H1, H4, K1, M2	
**Early and Middle Pleistocene from China:**	[127]
Changyang, Chaoxian, Dingcun, Jianshi, Lantian, Meipu, Panxian Dadong, Tongzi,Yiyuan, Yuanmou, Zhoukoudian Locality 1 and 4	
	[126]
Zhoukoudian LC G1.66 (CAST), Zhoukoudian LC GI/II (CAST), Zhoukoudian LC H1.12 (CAST), Zhoukoudian LC A II, Zhoukoudian LC M, Zhoukoudian LC Kotzetang; Lantian Chenjiawo, Hexian, Kedung Brubus 1 (mand. A), Sangiran 1(mand. B),5 (mand. of 1939),6 (Meganthropus A),8 (Meganthropus B),9 (mand. C),22 (mand. F),33 (mand. H/Bk 7905), Sangiran Sb 8103	
**Late Pleistocene,**	
	[127]
Wajak (Java), Minagatawa (Japan), Tianyuan, Xujiayao, Huanglong Cave, Liujiang, Xintai, Zhiren Cave, Zhoukoudian (ZKD) Upper Cave, Luna, Bailian Cave, Baojiyan, Cangwu, Chuandong, Duan, Huli Cave, Jimuyan, Longlin, Xichou, Longtanshan	
Penghu 1 (*Homo sp.*) first archaic *Homo* from Taiwan	[127, 126]
Xiahe	[130, 126]
**West Asia**	
**Early Pleistocene** (Dmanisi)	[127, 126, 128]
D2735, D2600, D211	
**European Lower Pleistocene**	
Gran Dolina (Atapuerca, Spain), ATD6-5, ATD6-113	[130]
Sima dell'Elefante [TE] (Sierra de	
Atapuerca, Spain), ATE-9-1	
**European Archaic and Middle Pleistocene**	
**Proto-Neanderthal**	
Atapuerca Sima de los Huesos SH 1, 4, 5 (Cr.15), 7, 12, 15 (Cr.17), 19, 21 (Cr.5), 22, 26 (Cr.10), 27, 28	[130]
Mauer	[130, 126]
Arago II, XIII	[130, 126]
Montmaurin 1	[70, 130, 126, 127]
Ehringsdorf F	[130]
Visogliano 2	
Aubesier 11	

Samples included in mandibular morphological and metric comparison	
**Sample/specimens**	**Date source**
Mala Balanica (BH 1)	
Payre 15	
**Neanderthal**	
**European Neanderthal**	
Sima de los Huesos: AT 1, AT 75 (Individual VI), IV (AT 250/793), AT 300 (Individual XII), AT 605 (Individual XXII), AT 607 (Individual XXIII), XXI (CAST) [AT 888/721/776/3867-3878], 950 (Individual XXVIII), XIX (AT 952/505/604), Arcy II (Grotte de l'Hyène), Banyoles (Bañolas Quarry), El Sidrón: 1,2,3; Circeo: 2,3; Krapina: 57 (Mandible G), 58 (Mandible H), 59 (Mandible J), Abri Suard S36 (La Chaise), Bourgeois-Delaunay 1 (La Chaise), La Ferrassie 1, La Naulette 1, La Quina: 5, 9, Le Régourdou 1, Saint-Césaire 1, Sima de las Palomas: 1, 6, 23, 59, Spy 3, Subalyuk 1, Vindija: 206, 226, 231, 250,11.39, 11.40, 11.45, Weimar-Ehringsdorf F1009, Zafarraya 2	[126]
**Circeo 2**	**Original data**
**Circeo 3**	**Original data**
La Chaise (Bourgeois-Delaunay 1), Amud 1, Kebara 2, Shanidar 1, Tabun 1, Spy 1,	[130]
La Ferrassie 1, La Quina: H5, H9, Regourdou, Circeo 2, 3; Cova del Gegant, Vindija: 206, 207, 226, 231, Krapina: 57, 58, 59, 63, 66,,68, 69; Zafarraya 2, Sima de las Palomas: 1, 6,,23, 59	
**Asian Neanderthal**	
Amud 1, Chagyrskaya 6, Kebara 2, Shanidar 2,4; Tabun 1	[126]
H. sapiens	
Early H. sapiens	
Dar es Soltane II H5, Dire Dawa, Jebel Irhoud 11, El Har(h)oura 1 (Zouhrah Cave), Klasies River: KRM 21776, KRM 13400, KRM 14695, KRM 16424, KRM 41815, Qafzeh: 25, 9, Skhul: IV, V, Tabun2_C2, Témara 1 (Grotte des Contrebandiers)	[126]
**Late Pleistocene east Asian humans**	
Minatogawa: 1 (CAST), A (CAST), Tam Hang South: 10,11, 13bis, 13ter, Tam Pa Ling 2, Tianyuan (Tianyuandong), Wadjak 2 (CAST), ZKD UC: 101 (CAST), 104 (CAST), 108 (CAST)	[126]
**Upper Palaeolithic *H. sapiens***	
Abri Pataud 1, Arene Candide: 18 (Specimen 20), 2, Asselar, Barma del Caviglione, Chancelade, Cro Magnon: 1, 3, Dar es Soltane: II 2, II 3, Dolni Věstonice: 3, 13, 14, 15, 16, El Mirón, Grotte des Enfants 4, Hayonim: 8,17, 19, 20, 25, 27, 29, 29a, Isturitz: 106, 115, Le Roc: 1, 2, Muierii 1, Nahal Oren: 6, 8, 14, 18, Nazlet Khater 2, Oase 1, Oberkassel: 1 (male),2 (female), Ohalo: II 1, II H2, Pavlov 1, Předmostí: 21 (female), 3 (male), Sunghir: 1, 6, Villabruna 1	[126]

Samples included in mandibular morphological and metric comparison	
**Sample/specimens**	**Date source**
**Modern Humans**	
Recent *H. sapiens* N 77	[130] (A morphometric comparison of upper Pleistocene mandibles. In Anthropology. Northern Illinois University. DeKalb,IL)

Fossil and recent comparative samples used for dental crown linear measurements	
**Samples/specimens**	**Date source**
**Superior Maxillary**	
**Proto-Neanderthal**	
Arago 21,26, 9, 54,14,68	[131, 132]
Atapuerca Sima de los Huesos	[133]
Atapuerca Sima de los Huesos: AT-1, AT-2, AT-3, AT-75, AT-83, AT-172, AT-250+793, AT-300, AT, 301, AT-303, AT-304, AT-505+952+604, AT-511, AT-605, AT-888+721, AT-950, AT-1157, AT-1775, AT-1957, AT-2193	[70]
**Neanderthal**	
Krapina, Scladina, La Quina, Roc de Marsal, Sima de las Palomas, El Sidrón, Spy, Engis (Schmerling Caves), Le Moustier, Wezmeh	[131]
Krapina	[70]
**Inferior Maxillary**	
**Proto-Neanderthal**	
Montmaurin-LN	[70]
Mala Balanica (BH-1)	
Arago 2,13	
Mauer	
Atapuerca Sima de los Huesos	[133]
Atapuerca Sima de los Huesos	[70]
AT-1, AT-2, AT-3, AT-75, AT-83, AT-172, AT-250+793, AT-300, AT, 301, AT-303, AT-304, AT-505+952+604, AT-511, AT-605, AT-888+721, AT-950, AT-1157, AT-1775, AT-1957, AT-2193 Burgos (Spain)	
**Neanderthal**	
Krapina: J, H, G	[70]
Ehringsdorf F, La Ferrassie 1, La Quina 5, Regourdou, Spy I, Amud 1, Shanidar 1	

Fossil and recent comparative samples used for crown 3D complete crown and lateral 3D measurements

**Inferior Maxillary (M3)**	
**Circeo 11 (C11)**	**Present study**
**Circeo 12 (C12)**	**Present study**
Circeo 2	Original data
Circeo 3	Original data
Australopithecus	
A. africanus (mandibular M3)	[30]
STW 412B, STW 529, STW 560B	
H. antecessor (TD6 level)	[134, 33]
Atapuerca-Gran Dolina: ATD6-5, ATD6-113, ATD6-96	
**North African *Homo* (NAH)**	
Tighenif: Tighenif_2	[134, 33]
***H. erectus* (HER)**	
Sangiran: NG9107.2	[134, 33]
**Proto-neanderthal**	
Sima de los Huesos (SH)	[134, 33]
Atapuerca-SH: AT-30, AT-811, AT-143, AT-1468, AT-599, AT-942, AT-1959, AT-2438, AT-2273, AT-2777, AT-3182, AT-3943	
**European Middle Pleistocene *Homo* (EMPH)**	
Montmaurin-La Niche	[134, 33]
**Neanderthal (NEA), average (N.11 and N.6) and individual values**	
Abri Suard: S36, S43	[134, 33]
Krapina: KRD9, KRD57, KRD85, KRD5, KRD7, KRD106	[134, 33]
Le Moustier 1 (2)	[134, 33]
Regourdou 1 (2)	[134, 33]
La Quina: Q760-H9	[134, 33]
Abri Bourgeois-Delaunay: BD1	[135, 133, 33]
Combe Grenal: CG XII	[134, 33]
**Modern humans (MH), average and individual values**	[134]
Modern humans from Europe (n = 8): B998-scht3, B996-scht1, B996-scht1, B996-scht2 M3g, B998-scht2-mand2, MH-CZ, MH-UdP, San Canziano	
Modern humans from South Africa, North America and Europe (n = 44)	[33]
Modern humans from Europe (n = 8)	

Fossil and recent comparative samples used for crown 3D complete crown and lateral 3D measurements
Modern humans from Spain (n = 20)

Linear diaphyseal diameters and cross-sectional geometric parameters for pleistocene *homo* femora

Samples/specimens	Date source
**Proximal and midshaft Femur**	[47, 80]
Early Pleistocene	
Middle pleistocene	[80, 136]
Neanderthal	[80, 136]
MPMH (Middle Paleolithic modern human)	[136]
EUP (Early Upper Paleolithic)	[80]
EUP/MUP (Early/Mid Upper Paleolithic modern humans)	
**Distal Femur**	
NTK-F-07-05 (Central Narmada Valley, India)	[137]
Measurements on original Eastern Indian housed in the Anthropological Survey of India, Paleoanthropology Laboratory, Kolkata and on original at Tautavel, France	
Neanderthal	
Homo erectus (Java)	
Homo erectus (Arago Tauteval)	
Homo sapiens (Modern Eastern Indians)	

Table S2. Circeo 5 (C5) *calvarium* morphology compared with lower, middle and upper Pleistocene specimens.

Specimens	Styloid process fused to the *basicranium*	Specimens	Anterior mastoid tubercle	Specimens	Bridge of bone on the digastric groove
**C5** (present study)	**No**	**C5**	**No**	**C5**	**Yes**
**Circeo1**(original data)	**Yes**	**Circeo 1**	**Yes**	**Circeo 1**	**Yes**
ZKD 102	Yes	ZKD 101-102	No	ZKD 101-102	No
Qafzeh 9	Yes	Dolni Vestonice 3	No	Dolni Vestonice 3	No
Skhul 5	Yes	Predmostì 3	No	Predmostì 3	No
Dolni Vestonice 3	Yes	Predmosti 4	No	Skhul 5	No
Predmostì 3	Yes	Qafzeh 9	No	Qafzeh 6-7- 9	No
Predmosti 4	Yes	Skhul 5	No	Spy 1	Yes
Spy 1	Yes	Gibraltar 1	No	Gibraltar 1	Yes
Spy 2	Yes	Spy 1	Yes	Saccopastore 1	Yes
La Quina 5	Yes	Spy 2	Yes	Saccopastore 2	No
La Chapelle	Yes	Saccopastore 1	No	La Quina 5	No
La Ferrassie 1	Yes	La Quina 5	Yes	La Ferrassie 1	Yes
Le Moustier	Yes	La Chapelle	Yes	La Chapelle	Yes

Specimens	Styloid process fused to the basicranium	Specimens	Anterior mastoid tubercle	Specimens	Bridge of bone on the digastric groove
Engis 2	No	La Ferrassie 1	Yes	Le Moustier	Yes
La Quina 18	Yes	Amud	Yes	Engis 2	Yes
Shanidar 1	No	Saccopastore 2	No	La Quina 18	Yes
Krapina sample	Yes	Shanidar 2	No	Tabun C1	Yes
Steinheim	Yes	Shanidar 5	No	Amud 1	Yes
Petralona	Yes	La Quina 18	Yes	Shanidar 1	No
SH sample	Yes	Krapina sample	No	Krapina sample	Yes
Laetoli 18	No	BroKen Hill	No	Steinheim	No
Omo 2	Yes	Ndutu	No	La Chaise Suard	Yes
Ndutu	Yes	Omo 2	No	Biache-Saint-Vaast	Yes
BroKen Hill	Yes	Laetoli 18	No	SH sample	No
Yunxian	No	SH sample	No	Laetoli 18	Yes
Xujiyao	Yes	H. erectus sample	No	Omo 2	No
Hexian	No	ER 3883	No	Ndutu	No
Dali	Yes	ER 3733	No	BroKen Hill	No
Narmada	Yes	OH 9	No	Ngandong 12	No
Ngandong sample	No			Skull XI	No
Sangiran 4	No			Skull III	Yes
ZKD sample	No			Sangiran sample	No
OH 9	Yes			OH 9	No
ER 3733	Yes			ER 3733	No
ER 3883	No			ER 3883	No
ER 3735	Yes				

Comparison data taken from *Ref.* [41]

Table S3. Linear skull measurements (mm). Circeo 4 (frontal bone) and Circeo 5 *calvarium* (present study) individual values compared with other specimens of Pleistocene (Proto-neanderthal, Neanderthal, *H. erectus* early and late and *H. sapiens*). **SH**, Sima de los Huesos; **ZKD**, Zhoukoudian; **SM**, Sambungmacan; **Ng**, Ngadong; **WLH 50**, Australian sample.

	**Length Max Glabella-Opisthocranion**	**Breadth Max Euryon-Euryon**	**Frontal diameter Minimum frontal breadth**	**Frontal Breadth Max Maximum frontal breadth**	**Supraorbital torus breadth**	**Porion-Bregma height**	**Max width within the ext. orbital apophyses bi-frontomalare temporale breadth**	**Postorbital Breadth**
**Sample**	**g-op M1**	**eu-eu M8**	**ft-ft M9**	**co-co M10**		**M20**	**fmt-fmt M43**	
**Circeo 4**	**-**	**-**	**112**	**-**	**124,9**	**-**	**(106 -110)**	**106,5**
**Circeo 5**	**195**	**145**	**112**	**123**	**(115.6-119)**	**99**	**104,1**	**106**
Circeo 1	204	155	109	127	120	111	-	-
Ceprano (original data)	-	-	(106)*	(120)*	131	-98	(125)*	(107)*
Buia	204	-	-	-	-	-	-	-

	Length Max Glabella-Opisthocranion	Breadth Max Euryon-Euryon	Frontal diameter Minimum frontal breadth	Frontal Breadth Max Maximum frontal breadth	Supraorbital torus breadth	Porion-Bregma height	Max width within the ext. orbital apophyses bi-frontomalare temporale breadth	Postorbital Breadth
**Sample**	**g-op M1**	**eu-eu M8**	**ft-ft M9**	**co-co M10**		**M20**	**fmt-fmt M43**	
Daka-Bouri BouVP2/66	180	145	89	105	125	101	124	95
Arago 21	-	-	109,1	113,1	-	-	123	-
Petralona 1	209	150	108	120	133	-	130	-
Atapuerca SH 3	-	-	102,1	115	-	-	-	-
Atapuerca SH 4	201	164	117	126	-	-	-	-
Atapuerca SH 5	185	146	105,7	118,5	-	-	-	-
Atapuerca SH 6	186	100	-	-	-	-	-	-
Broken Hill	206,3	146,9	97,6	118,3	139	107,1	134,4	104
Biache-Saint-Vaast 2	-	-	104,4	-	128,7	-	127,6	-
Gibraltar 1	193	149	100	-	-	107	118	-
Saccopastore 1	181	142	119	-	-	101	114	-
Feldhofer (Nea 1)	199	146,7	108	-	120,5	-	121,6	-
Le Moustier	196	150	-	-	-	111	-	-
La Quina 5	205	138	102	-	111,7	-	112,5	-
La Ferrassie 1	208	158	108	126	119,9	114	120	-
Krapina 3	-	-	103	-	120,7	-	120,7	-
La Chapelle-aux-Saints 1	208	157	106	129	123,5	111	118	-
Amud 1	204	158	113,5		125,6	120	125,3	
Tabun 1	183	141	-	-	-	98	113,9	-
Shanidar 1	207	154	-	-	-	-	-	-
Spy 1	209	144,3	-	-	-	111	.	-
Spy 2	209	153,2	-	-	-	114	-	-
Kocabaş	-	-	88	106	-	-	118	-
Dmanisi 3444	163	-	67,5	94	104	89	105	78
Dmanisi 2282	166	-	66	-87	-	93	105	86
Dmanisi 2280	-	-	74	106	-	-	114	-
Dmanisi 2700	155	-	67	-85	-	-	97	-
Dmanisi 4500	165	-	-	-	112	75	-	76
KNM-ER 1813	145	-	66	88	99	80	93	69
KNM-ER 1470	168	-	70	90	115	94	106	82
KNM-WT 15000	175	-	73	97	-	-	107	-
KNM-ER 3733	182	127	83	109	119	96	116	92
KNM-ER 3883	182	-	80	108	120	93	115	88
OH 9	206	138	84	105	135	99	130	100
Trinil 2	183	134	85	103	-	-	-	-
Sangiran 2	183	141	82	102	104	94	-	84
Sangiran 17	207	144	96	115	125	104	119	101
Sangiran 9	186	-	-	-	111	93	-	87
Bukuran	194	-	-	-	113	98	-	88
Yunxian	-	-	102	136	-	-	130	-

	Length Max Glabella-Opisthocranion	Breadth Max Euryon-Euryon	Frontal diameter Minimum frontal breadth	Frontal Breadth Max Maximum frontal breadth	Supraorbital torus breadth	Porion-Bregma height	Max width within the ext. orbital apophyses bi-frontomalare temporale breadth	Postorbital Breadth
**Sample**	**g-op M1**	**eu-eu M8**	**ft-ft M9**	**co-co M10**		**M20**	**fmt-fmt M43**	
Nankin (Hulu cave)	-	-	83	101	-	-	110	-
ZKD mean	194	134	86	-	-	-	-	-
ZKD 2	194	-	84	-	-	-	-	-
ZKD 3	188	-	81	105	-	-	109	-
ZKD 5	-	-	87,4	-	-	-	-	-
ZKD 10	199	150	89	-	119	106	-	98
ZKD 11	192	145	84	106	113	94	111	93
ZKD 12	195,5	147	91	110	118	101,5	119	95
Nanjing 1	186	-	-	-	-	-	-	-
SM 1	199	146	102	-	118	107	-	107
SM 3	178,5	127	101	110	114	98	112	101
SM 4	199	-	-	-	122	102	-	116
Lazaret 24	-	-	98,4	-	-	-	121	-
Ng 1	195,6	150,2	-	119	-	-	-	105
Ng 5	220,3	150,7	100	123,5	-	-	116	111
Ng 6	221	147,4	101	117,5	122	112	-	108
Ng 7	192	142	104	119	121	103	-	106
Ng 9	202	159	-	120,5	-	-	-	109,6
Ng 10	202	158,5	102	123,1	124	109	121	110
Ng 11	203	150,7	112	122	132	112	-	114
Ng 12	201	-	103	117	124	108	123	107
Ngawi	187	-	-	144	114	102	-	-
Java range	177-219	127-149	79-102	-	-	329	-	-
Hexian	191	-	96	116	114	97	111	101
Dali	212,2	156,4	105,7	122,7	125	102,9	124,4	106,4
Harbin	221,3	164,1	116,1	128,1	-	113,9	140,2	-
Maba	196,5	-	98,1	116,6	-	-	117,2	-
Xuchang	217	177	122,8	138,8	-	114,6	140	-
Salkhit	-	-	98,06	-	-	-	-	-
WLH 50	212.2	151,6	112	124	-	-	-	115
Irhoud 1	198,9	151,5	108,5	-	-	112,4	127,2	-
Mladeč 1	-	142	-	-	-	-	-	-
Manot 1	-	135	-	-	-	-	-	-
Skhul 4	206	148	-	-	-	-	-	-
Skhul 5	192	143	103,5	-	123,8	-	123,8	.
Skhul 9	195	145	78	102	-	99	-	-
Qafzeh 6	196	144,5	-	-	-	-	-	-
Qafzeh 9	198	140	103	-	111,7	-	111,7	-

Comparison data was taken from *Refs.* in Table S1.

* Restoration

Table S4. Occipital bone linear measurements (mm). Circeo 8 and Circeo 5 occipital bones individual values compared with other specimens of Pleistocene (Proto-neanderthal, Neanderthal, *H. erectus* early and late and *H. sapiens*).

**Sample**	**Bi-asterionic breadth M12**	**Lambda-*inion* M31(1)**	**Lambda-*asterion* M30(3)**	***Inion-asterion***
**Circeo 8** (present study)	**123,2**	**61,5**	**86,9**	**~73,2**
**Circeo 5** (present study)	**118,5**	**59,8**	**86,3(r) 87,8(l)**	**71,1**
Circeo 1	127	63,1	94,3	67,7
Ceprano (original data)	-125	-62	-96,7	-78,9
Broken Hill	120,4	-	-	-
Daka-Bouri BouVP2/66	116	-	-	-
Atapuerca SH 1	120	-	-	-
Atapuerca SH 2	136	-	-	-
Atapuerca SH 3	113,5	-	-	-
Atapuerca SH 4 (+AT 600)	132	-	-	-
Atapuerca SH 5 (+AT 700)	116,5	-	-	-
Atapuerca SH 6	117,6	-	-	-
Atapuerca SH 8 (+AT 433)	141	-	-	-
Atapuerca SH occipital II	112,6	-	-	-
Atapuerca SH occipital IV (Late adolescent)	112,5	-	-	-
Petralona	124	63,5	-	-
Bilzingsleben	138,6	-	-	-
Steinheim	107	-	-	-
Swanscombe	121,8	-	-	-
Apidima 1	115	-	-	-
Biache-Saint-Vaast 1(Late adolescent/ adult)	108	-	-	-
Krapina 5		61,6	86,3	71,8
La Chaise Suard S9	111	55	-	-
La Chaise Bourgeois-Delaunay BD6	121,5	61,2	76,9	57,1
La Chaise Bourgeois-Delaunay BD17	121	-	-	-
Gibraltar 1	98	57,4	86,2	67,7
La Chapelle-aux-Saints 1	126,1	60,6	93,3	71,6
La Ferrassie 1	121,6	59,5	89,6	68,6
Feldhofer (Nea 1)		59	-	-
Saccopastore 1	109,7	57,5	92,5	67,3
La Quina H5	112	60	-	-
Spy 1	121	58,9	90,7	66,8
Spy 2	124	60	89,7	70,7
Salzgitter-Lebenstedt	117,5	60,5	79,9	71,3
Šal'a	124	-	-	-
Amud 1	134	-	-	-

Sample	Bi-asterionic breadth M12	Lambda-*inion* M31(1)	Lambda-*asterion* M30(3)	*Inion-asterion*
El Sidrón SD-1219	120,6	59,4	90,4	68,7
Fontéchevade II	126	-	-	-
Shanidar 1	118,2	68,4	-	-
Tabun C1	107	56,5	79,6	63,5
KNM-ER 1813	94	-	-	-
KNM-ER 1470	104	-	-	-
KNM-WT 15000	106	-	-	-
KNM-ER 3733	119	-	-	-
KNM-ER 3883	115	-	-	-
OH 9	123	-	-	-
Dmanisi 3444	104	-	-	-
Dmanisi 2282	103	-	-	-
Dmanisi 2700	105	-	-	-
Dmanisi 4500	92	-	-	-
Zhoukoudian 2	103	-	-	-
Zhoukoudian 3	117	-	-	-
Zhoukoudian 10	116	54	-	-
Zhoukoudian 11	120	51	-	-
Zhoukoudian 12	118	60	-	-
Nanjing 1	111	-	-	-
Trinil 2	92	-	-	-
Sangiran 2	122	54	-	-
Sangiran 4	135	51	-	-
Sangiran 9	117	-	-	-
Sangiran 10	120	50	-	-
Sangiran 12	125	48	-	-
Sangiran 17	124	58	-	-
Bukuran	126	-	-	-
Sambungmacan 1	126	59	-	-
Sambungmacan 3	118	50,5	-	-
Sambungmacan 4	133	-	-	-
Ngandong 1	131	87,6	52	77,8
Ngandong 5	128	85,3	62,4	73,2
Ngandong 6	120,5	86,4	59,7	73,3
Ngandong 7	124	-	59	-
Ngandong 9	123,9	80,5	61,8	79,5
Ngandong 10	127	84,4	55,1	67,3
Ngandong 11	126,7	85,2	57	76,4
Ngandong 12	126	-	66	-
Ngawi	127	-	-	-
Java range	92-142	-	45-66	-
Hexian	131	-	-	-
Dalì	121,4	71	100/92	-
Harbin	134,4	-	-	-
WLH 50	123	92,8	66	72,6

Sample	Bi-asterionic breadth M12	Lambda-*inion* M31(1)	Lambda-*asterion* M30(3)	*Inion-asterion*
Xuchang XUC1	136,7	85	102,1	80,7
Irhoud 1	123,2	-	-	-
Mladeč 1	112	-	-	-
Manot 1	95	-	-	-
Qafzeh 3	121	-	-	-
Qafzeh 6	120	-	-	-
Qafzeh 9	111	62	-	-
Qafzeh 11 (Sub-adult 12y)	107	-	-	-
Skhul 4	132	-	-	-
Skhul 5	122	66	-	-
Skhul 6	140	-	-	-
Skhul 9	120	64	-	-
Dolní Věstonice 3	108	-	-	-
Dolní Věstonice 13	120	-	-	-
Dolní Věstonice 14	115	-	-	-
Dolní Věstonice 15	112	-	-	-
Dolní Věstonice 16	114	-	-	-
Pavlov 1	117	-	-	-
Předmosti 3	112	-	-	-
Předmosti 9	107,5	-	-	-
Předmosti 13	104	-	-	-

Comparison data was taken from *Refs.* In Table S1

Table S5. Frontal bone thickness (measurements mm). Circeo 4 frontal bone and Circeo 5 *calvarium* individual values compared with other specimens of Pleistocene. (Proto-neanderthal, Neanderthal, *H. erectus* early and late and *H. sapiens*). **SOTTM**, Supraorbital Torus Thickness at Midorbit.

Sample	Bregma	SOTTM	Torus highest point
**Europe**			
**Italy**			
**Circeo 4** (present study)	**-9,6**	**15,4**	**18,7**
**Circeo 5** (present study)	**10,3**	**13,2**	**21,2**
**Ceprano** (original data)	(11,9)*	21 (l)	21,3 (l)
**Greece**			
Petralona 1	10,5	-	-
**Spain**			
Carihuela	-	6-3,5	-
**England**			
Swanscombe	7	-	-
**France**			
Lazaret 24	-	11	-
Biache St-Vaast 2	-	14-dic	-
Arago 21	-	11-dic	-

Sample	Bregma	SOTTM	Torus highest point
La chapelle	5,5	-	-
La Quina 5	5	-	-
La Ferrassie 1	6	-	-
Le Moustier	6	-	-
**Croatia**			
Vindija 261	5,9	-	-
Krapina 3	8,2		
Krapina D	8,5	-	-
Krapina 16	7	-	-
**Belgium**			
Spy 1	8	-	-
Spy 2	7	-	-
**Spain**			
Gibraltar	7	-	-
**Germany**			
Steinheim	6	-	-
Bilzingsleben	7	-	-
Neanderthal	7,5	-	-
**Israel**			
Amud 1	9	-	-
Tabun 1	4	-	-
**Morocco**			
Salè	8	-	-
**Africa**			
**Earliest Africa**			
KNM-ER 3733	-	8,5	-
KNM-ER 3883	-	13	-
KNM-WT 15000	-	10,4	-
OH9	-	18	-
SK 847	-	7	-
**Early Africa**			
OH12	10	-	-
Daka (Bou-VP-2/66)	-	18,5	-
Buia (UA 31)	-	17	-
Broken Hill	8,8	-	-
Florisbad	12	-	-
**Georgia**			
Dmanisi 2280	-	11	-
Dmanisi 2282	-	10	-
Dmanisi 2700	-	9	-
**China**			
Lantian	16	-	-
Dali	-	20	-
**Middle China**			
Zhoukoudian 2	7,5	17,4	-
Zhoukoudian 3	9,5	12,1	-
Zhoukoudian 4	10,5	-	-

Sample	Bregma	SOTTM	Torus highest point
Zhoukoudian 5	7	-	-
Zhoukoudian 10	10	16,5	-
Zhoukoudian 11	7	13,6	-
Zhoukoudian 12	9,5	14,6	-
**Earliest Indonesia**			
Trinil 2	9	-	-
Sangiran 27	-	28	-
**Early Indonesia**			
Sangiran 2	8,8	12	-
Sangiran 3	10,5	-	-
Sangiran 4	5,5	-	-
Sangiran 10	8	19	-
Sangiran 12	9	-	-
Sangiran 13	10	-	-
Sangiran 17	9	18	-
Sangiran 18	11	-	-
Ngawi 1	-	16	-
Sambungmacan 1	-	15	-
Sambungmacan 3	11	13	-
Sambungmacan 4	-	15	-
**Late Indonesia**			
Ngandong 1	9,5	13	14,7
Ngandong 3	10	-	-
Ngandong 5	8,8	14,5	17,9
Ngandong 6	12	-	-
Ngandong 7	-	15	-
Ngandong 9	11,5	12	15,9
Ngandong 10	-	9	18,5
Ngandong 11	11	12	22
Ngandong 12	-	14	-
**Pleistocene/Holocene**			
**Australian**			
WLH 50	14	21,5	21,5
Mungo 1	4,5	-	-
Mungo 3	7	-	-
Kow Swamp 16	7,5	-	-
Keilor	9	-	-
Tandou 2	8	-	-
Range Pleistocene/Holocene	6,1-17,2	-	-
**China**			
Xuchang (XUC) 1	8	10	-
Xujiayao10	8,6	-	-
Xujiayao 6	6,5	-	-
Xujiayao 4, 5	9	-	-
Maba	7	-	-
**Indonesia**			
Wadjak 1	8	-	-

Sample	Bregma	SOTTM	Torus highest point
**Ethiopia**			
Omo 1	8	-	-
Omo 2	9	-	-
**Tanzania**			
Laetoli 18	12	-	-
**South Africa**			
KRM41658	7		
**France**			
Cro-Magnon 1	8	-	-
**Czech Republic**			
Mladeč 1	7	-	-
Předmostí 3	7,5	-	-
Předmostí 9	4,5	-	-
**Jersey**			
Cotte de St. Brelade	12	-	-
**Germany**			
Oberkassel 1	10	-	-
Oberkassel 2	8		
**Morocco**			
Irhoud 1	7,6	-	-
**Israel**			
Qafzeh 9	6	-	-
Skhul 5	7,5	-	-
Manot 1	6	-	-
**Holocene**			
French (N. 200)	5,4	-	-
Yuendumu (N. 20)	7,4	-	-
Africans (N. 64)	6,7	-	-
Chinese (N. 49)	6,4	-	-
Phillipines (N. 22)	6,7	-	-
Hebrides (N. 16)	7	-	-
American white (N.445)	5,88	-	-
Belgian (N. 200)	5,3	-	-
Byblos (N. 13)	7,4	-	-
Sialk Copper Age (N. 10)	7,1	-	-
Sialk Iron Age (N. 20)	5,7	-	-
Hastière (N. 24)	6,4	-	-
French Neolithic (N. 15)	6,8	-	-
Susa (N. 14)	6,5	-	-
Palmyra (N. 19)	6,4	-	-

Comparison data was taken from *Refs.* in Table S1

*The thickness was measured on the sagittal frontal in proximity bregma

(N.), Number

Table S6. Parietal bone thickness (measurements mm). Circeo 4 parietal bone and Circeo 5 *calvarium* individual values compared with other specimens of Pleistocene/Holocene.

**Sample/Specimens (*Genus*)**	**Parietal to bregma**	**Parietal to lambda**	**Parietal to middle eminence**	**Parietal to *asterion***
**Europe**				
**Circeo4** (present study)	**-8,8**	-	-	-
**Circeo5** (present study)	**10, 3** (5 post bregma)	**11** (6 lambda thinning)	**12**	**10**
Circeo 1	-	-	**7**	-
**Proto-Neanderthal**				
Ceprano (original data)	-	-	12 (right)	-
Arago 47	-	-	-	14,8
Petralona 1	-	11	9	8
Reilingen	-	-	-	8,25
Steinheim	-	-	6,5	-
Swanscombe	-	-	10,5	-
Proto-Nea range	-	6,5-11	-	8-14,8
**Neanderthal Early-Late**				
Ehringsdorf 1	-	-	10	-
Ehringsdorf 2	-	-	17	-
Neanderthal	-	-	10	-
Kulna	-	.-	11,3	-
Amud 1	-	-	8	-
La Chapelle aux Saints 1	-	8	7,5	5,5
La Quina H5	-	-	6,3	-
La Quina 13	-	-	7,1	-
La Ferrassie 1	-	5,5	7	5
Le Moustier	-	-	6,8	-
Tabun 1	-	-	5	-
Shanidar 1	-	-	8	-
Shanidar 2	-	-	8,2	-
Shanidar 4	-	-	8,1	-
Shanidar 5	-	-	9	-
Vindija 204	-	7,2	8,3	-
Vindija 293	-	-	9,1	-
Krapina D	-	-	7	-
Krapina E	-	-	7,5	-
Krapina 3	-	-	8,5	-
Krapina 5 (parietal)	-	8,3	7,5	-
Krapina 6 (+Occ.6)	-	6	-	-
Krapina 16	-	6	7,5	-
Krapina 20	-	-	8	-
Krapina 21	-	7,1	6	-
Krapina 32-32,1 (?)	-	9,9	8	-
Krapina 34,1	-	7,1	7	-
Krapina sample	-	-	6 to 8	-
Gibraltar	-	-	9,5	-

Sample/Specimens (*Genus*)	Parietal to bregma	Parietal to lambda	Parietal to middle eminence	Parietal to *asterion*
La Chaise Abri Suard S1 (S1+S17)	-	12	-	-
La Chaise Abri Suard 16		8,3	-	-
La Chaise Bourgeois-Delaunay BD17a, b	-	6	-	4,5
La Chaise Bourgeois-Delaunay BD6	-	10	-	-
Feldhofer (Nea1)	-	8	10	-
Spy 1	-	10	9,5	7
Spy 2	-	8	9	6
La Pélénos	-	6	-	-
Gibraltar	-	-	9,5	-
Nea range	-	4,8-11,3	-	4,5-7,1
**Africa**				
**Early Africa**				
Melka Kunture	-	-	15	-
Ndutu	-	-	11,5	
Florisbad	-	-	12	-
Broken Hill	-	-	9,5	-
**Asia**				
Homo erectus				
**Middle China**				
Zhoukoudian 1	-	-	5	-
Zhoukoudian 2	-	-	9,75	-
Zhoukoudian 3	-	-	11,25	-
Zhoukoudian 4	-	-	10	-
Zhoukoudian 5	-	-	10,5	-
Zhoukoudian 10	-	-	9,25	-
Zhoukoudian 11	-	-	16	-
Zhoukoudian 12	-	-	8,75	-
**Earliest Indonesia**				
Trinil 2	-	-	9	-
**Early Indonesia**				
Sangiran 2	-	-	11	-
Sangiran 3	-	-	8,5	-
Sangiran 4	-	-	9,3	--
Sangiran 10	-	-	11	-
Sangiran 18	-	-		-
Sambungmacan3 (Middle Pleist.)		11,5	12	15
**Later Indonesia**				
Ngandong 1	7,9	10,9	7	-
Ngandong 3	-	-	8	-
Ngandong 5	11,5	12,5	9,5	17
Ngandong 6	-	12,1	-	-
Ngandong 9	7,6	-	-	14,5
Ngandong 10	10,1	9,9	9	16
Ngandong 11	9,2	12	13	-
Hexian	-	-	13,5	-

Sample/Specimens (*Genus*)	Parietal to bregma	Parietal to lambda	Parietal to middle eminence	Parietal to *asterion*
**Pleistocene/Holocene**				
**China**				
Xuchang 1 (XUC)	-	-	7,9	-
Xujiayao 9	-	-	12,9	-
Xujiayao 6	-	-	7	-
Xujiayao 4,5	-	-	10,8	-
Xujiayao 10	7,7	-	12,6	-
Xujiayao 11	-	-	9,6	-
Dali	-	-	12	-
Maba	-	-	9	-
**Australian**				
WLH50	14,9	15	16	17
**Israel**				
Manot 1	-	-	6,5	-
Qafzeh 3	-	12	10	-
Qafzeh 5	-	-	8	-
Qafzeh 6	-	-	8	-
Qafzeh 7	-	-	5	-
Qafzeh 9	-	-	11	-
skhul 2	-	-	10,5	-
skhul 4	-	-	10	-
skhul 5	-	-	4,5	-
skhul 9	-	-	11	-
**South Africa**				
Boskop	-	-	14	-
KRM 41658	-	-	7	-
**Kenya**				
Lukenya	-	-	12	-
**France**				
Cro-Magnon 1	-	-	9,5	-
Cro-Magnon 2	-	-	6,5	-
Cro-Magnon 3	-	-	5,5	-
**Czechia Republic**				
Předmosti 1				
Předmosti 3	-	-	5	5,5
Předmosti 4	-	-	6	6
Předmosti 9	-	-	6	4
Předmosti 10	-	-	5	5
Předmosti 14	-	-	7,5	-
Mladeč 1	-	-	3,5	-
Mladeč 5	-	-	7	-
Mladeč 6	-	-	8	-
**Jersey**				
Cotte de St. Brelade	-	-	6	-
**Germany**				
Oberkassel 1	-	-	5	-
Oberkassel 2	-	-	7	-

Sample/Specimens (*Genus*)	Parietal to bregma	Parietal to lambda	Parietal to middle eminence	Parietal to *asterion*
**Holocene**				
French (N. 200)	-	-	5,7	-
Yuendumu (N. 20)	-	-	4,3	-
Africans (N. 64)	-	-	7,7	-
Chinese (N. 49)	-	-	6	-
Phillipines (N. 22)	-	-	5,7	-
Hebrides (N. 16)	-	-	6,9	-
American white (N.445)	-	-	3,6	-
Belgian (N. 200)	-	-	5,7	-
Byblos (N. 13)	-	-	6,9	-
Sialk Copper Age (N. 10)	-	-	6,4	-
Sialk Iron Age (N. 20)	-	-	5,3	-
Hastière (N. 24)	-	-	6,7	-
French Neolithic (N. 15)	-	-	6,7	-
Susa (N. 14)	-	-	6,6	-
Palmyra (N. 19)	-	-	7,2	-

Comparison data was taken from *Refs.* In Table S1

Table S7. Frontal bone thickness (measurements mm). Circeo 4 frontal bone and Circeo 5 *calvarium* (present study); Ceprano *calvarium* (original data), *i*ndividual values compared with European specimens of Pleistocene.

	Italy			France		Spain	
**Sample**	**Circeo 4**	**Circeo 5**	**Ceprano**	**Arago 21**	**Biache St-Vaast 2**	**Carihuela**	**Lazaret 24**
**Frontal**							
Max	13,5	12,7	-	6	6	6	6
Min	10-nov	7,2	-	2	2	1	1
Bregma	9,6	10,3	(11,9)*	-	-	-	-
Right median frontal point	12,7	11,7	11,2	8,7	6	3,5	7
Left median frontal point	12,7	11,7	-	8,6	5	3	-
Right frontal eminence	11	10	-	7	-	4	6
Left frontal eminence	12	10	-	7	-	4	-
Torus highest point	18,7	21,2	21,3	-	-	-	-
Right supraorbital torus central	15,4	13,2	-	11	14	6	11
Lateral	13,3	12	15	-	-	-	-
Medial	16,2	14,2	18,9	-	-	-	-
At corner	13,7	~ 10,2	9	-	-	-	-
Left supraorbital torus central	15,1	13,2	21	12	12	3,5	-
Thickness at the glabella	~ 23,3	24	-	-	-	-	-
Right coronal border	11,1	-	-	2 to 4	-	4,5 to 6	5 to 6
Left coronal border	10,9	7,1 to 8,2	-	-	-	3 to 4,5	-
Right stephanion	-	8	-	8,8	-	4,5	6,5
Left stephanion	12,2	7,7	-	8,7	-	4	-
Glabella (inner frontal crest)	~ 33,2	25	-28	8,7	28	13,5	36
Ophryon (inner frontal crest)	17,1	13	-20	12,5	12	11	10

		Italy		France		Spain	
**Sample**	**Circeo 4**	**Circeo 5**	**Ceprano**	**Arago 21**	**Biache St-Vaast 2**	**Carihuela**	**Lazaret 24**
Posterior end of the internal frontal crest	10,6	12,1	-11	-	-	7	8
Anterior end of the frontal crest	-	~ 20,1	-21	-	-	-	-

Comparison data was taken from *Refs.* In Table1

*The thickness was measured on the sagittal frontal in proximity bregma

Table S8. Occipital bone thickness (measurements mm). Circeo 8 and Circeo 5 occipital bones individual values compared with other specimens of Pleistocene (Proto-neanderthal, Neanderthal, *H. erectus* early and late and *H. sapiens*).

**Sample/specimens** (*Genus*)	**Lambda**	***Inion***	**internal occipital protuberance**	***Asterion***
**Circeo 8** (present study)	**10,5**	Concretions	Concretions	**12,9**
**Circeo 5** (present study)	**13,5**	**17**	**20**	**11**
Circeo 1	**-**	**11**	**-**	**-**
**Proto-Neanderthal**				
Ceprano (original data)	12	-22,9	-19	14 (r)
Petralona	10	-	-	9,5
Swanscombe	-	9	-	-
Atapuerca SH1	-	-	-	9,5
**Neanderthal (Early)**				
Apidima 1	7,9	-	-	-
Apidima 2	8,3	9,3	-	-
Biache-Saint-Vaast	-	-	-	10,4
Krapina 5	8,8	12,1	-	9
Krapina 6 (+Occ 6)	-	11,9	-	4,5
Krapina 8	-	-	-	8,9
Krapina 10	-	-	-	9
Krapina 11	-	-	-	12
Krapina 18,1	-	-	-	9,3
Krapina 18,2	-	-	-	11,2
Krapina 18,5	-	12,8	-	-
Krapina 18,6	-	-	-	11,7
Krapina 18,9	-	10	9,8	-
Krapina 2 (Juvenile; 10-12)	5	-	-	5
La Chaise Abri Suard S9	11	-	-	-
**Neanderthal (Early/Late)**				
Gibraltar	-	10	-	-
**Neanderthal (Late)**				
El Sidrón SD-1149	-	12	-	-
El Sidrón SD-1219	-	12	-	-
Fontéchevade II	-	-	-	8
La Chapelle aux Saints 1	9	9	**-**	**-**

**Sample/specimens** (*Genus*)	**Lambda**	***Inion***	**internal occipital protuberance**	***Asterion***
La Ferrassie 1	6,5	10	-	-
La Ferrassie 3 (Sub-adult 10y)	3,5	-	-	-
La Quina H5	-	6	-	-
Feldhofer (Nea1)	9	13	-	-
Spy 1	10	10	-	-
Spy 2	9	11	-	-
Salzgitter-Lebenstedt	7,3	-	-	-
Tabun 1	7	6	-	-
Vindija 205	-	14	13	-
Vindija 252	-	-	-	7,6
Vindija 258	-	-	-	8
Vindija 281	-	-	-	7,1
Vindija 282	-	-	-	7,7
Petit-Puymoyen PPm7 (adolescent)	6,5	-	-	-
**Late *H. erectus***				
Ngandong 1	8	18,8	18,5	-
Ngandong 5	15	25	20	-
Ngandong 9	-	20	17	-
Ngandong 10	8,5	22	16,5	-
Ngandong 11	-	25	-	-
WLH 50	16,2	18	20	-
H. sapiens				
Qafzeh 3	12	-	-	-
Qafzeh 7	8	-	-	-
Qafzeh 9	8,5	-	-	-
Cro-Magnon 1	9	-	-	-
Mladeč 5	7,5	-	-	-

Comparison data was taken from *Refs.* In Table S1

Table S9. Circeo 6 mandible. Circeo 6 mandible (**present study**) compared with Circeo 2 and Circeo 3 mandibles (original data) and other specimens of Pleistocene:
ATE, (Atapuerca) Sima del Elefante (TE); **ATD**, (Atapuerca) Gran Dolina; **SH,** Atapuerca Sima de los Huesos; **NEA**., Neanderthals; **Hexian,** mandibular fragment (PA 831) Eastern China (Middle Pleistocene); **Penghu** Eastern China (**Penghu 1** Late Pleistocene); **Xiahe**; **Montmaurin-LN** (Montmaurin-La Niche, Middle Pleistocene)**; Oase1 Peştera Muierii,** (Central European Late Pleistocene); **MH**, Modern Humans; **MP**, Middle Pleistocene; **LP**, Late Pleistocene; **LMP**, Late Middle Pleistocene; **UP**, Upper Palaeolithic; **MF**, Mandibular *Foramen*; **Sy**, Symphysis; **Th**, Thickness; **H.MF**, Height of the body of the mandible at the level of the Mental *Foramina*; **H.Sy**, Symphysis Height (Id-gn, infradental- gnathion); **MF.Loc**, Mental *Foramina* Localization; **Th.MF**, Thickness of the body of the mandible at the level of the Mental Foramina; **a**, distance of *foramen* from the alveolar rim; **b**, distance of *foramen* from the rim basal. **ND**, Not Detectable; **(N)**, Number; **(M)** Mean; **(±)**, Standard Deviation.

Chin and Symphysis (Measurements in mm)

Sample/specimens	H. Sy	H. MF		Th. MF		Th. Sy	Th. M1	Th. M2	Th. M3	MF Loc. main foramen	MF number (N) & size** (long x high)		Bi-canine breadth
		right	left	right	left						right	left	
**Circeo6**	**37,5**	**30,1***<br>14 a<br>15,5 b	ND	**15**	ND	**17,3**	**-17,5**	-	-	(P4-M1)<br>alveolar closure	**(N) 1 oval**<br>**3,3 x 2,4**	**-**	**~ 31,5**
**Circeo2**	34,8	~ 34,2*<br>~ 19,5 a<br>~ 15,8 b	~ 34,2<br>ND a<br>~15,8 b	15,4	-16,7	15,1	15,4<br>(r)	15,8<br>(r)	16,4<br>(r)	M1	(N) 1/2<br>2,5 x 2,1	(N) 2<br>1,9 x 3	30,1
**Circeo3**	35,5	35,8<br>~ 20,7 a<br>~ 13,1 b	34,5<br>~ 17,5 a<br>~ 13,3 b	14,2	12,8	13,6	14,1(r)<br>13,3 (l)	14,6(r)<br>14,1(l)	15.1 (r)	P4- M1	(N) 2<br>3,3 x 2,2	(N) 2<br>1,8 x 1,9	37,5
**East Asia**													
Sangiran 9 [1]	~ 42	38,1	-	28,8	-	18	21,2	22,6	26,4	P4 (P2)	(N)1	-	-
Sangiran 22 [1]	~ **36**	31,1	31,4	-	16,6	16,1	17,1	18,1	-	P3/P4 (P1/P2)	(N)3	(N) 3	-
Hexian PA831 [2]	-	30,2<br>(M_1)<br>Middle distance (a-b)		20,5		-	20,5	22,5	22,2	(I) P4 (II and III) M1	(N) 3 oval<br>(I) 4,6x2<br>(II)3,6x1,5<br>(III)2,7x1,3	-	-
Xiahe LMP [3]	32,6	30,7		17,9		15,4	19,6	21,5	-	P4	-	-	42,6
Early Pleist Java<br>*H. erectus* [2,4]	(N) 2<br>(M) 39	(N) 10<br>(M) **36,7**<br>± 4,9		(N)10<br>(M) 20,1<br>± 3,1		(N)2<br>(M) 17	(N) 7<br>(M) 19,8	-	-	P3-P4	Single/Multiple		-
Asian *H.*<br>*Erectus* [3]	(N) 9<br>(M) **37.6**<br>± 5.66	(N) 13<br>(M) 30,9<br>± 7,93		(N) 13<br>(M) 18,2<br>± 4.30		(N) 11<br>(M) 15,9<br>± 3,87	(N) 12<br>(M) 18,06<br>± 3,63	(N) 8<br>(M) 17,80<br>± 2,40	-	-	-	-	(N) 4<br>(M) 35,28<br>± 2,58

Chin and Symphysis (Measurements in mm)

Sample/specimens	H. Sy	H. MF		Th. MF		Th. Sy	Th. M1	Th. M2	Th. M3	MF Loc. main foramen	MF number (N) & size** (long x high)		Bi-canine breadth
		right	left	right	left						right	left	
Early/ middle Pleist. China *H.erectus*[2,4]	(N) 3<br>(M) 33.1	(N) 8<br>(M) 2,.4<br>± 4,0		(N) 8<br>(M) 16,6<br>± 1,7		(N) 4<br>(M) 13.1	(N) 7<br>(M) 16,2	-	-	P4	multiple		-
Late Pleist.[2]	-	(N) 8<br>(M) 32,8<br>± 3,9		(N) 8<br>(M) 14,8<br>± 1,9		-	-	-	-	-	-	-	-
Penghu 1 (Taiwan)[2,3]	30,5	27,3		20,7		17,8	20,7	-	-	P3-P4	Single	-	-
**West Asia**													
Early Pleist. (Dmanisi)[2,4]	(N) 2<br>(M) 36,2	(N) 3<br>(M) 30,9<br>± 9.7		(N) 3<br>(M) 19,7<br>± 1,8		(N) 2<br>(M) 19,8	(N) 2<br>(M) 18,8	-	-	P3-P4	Single		-
**Africa**													
Pliocene-Early Pleistocene (*Australopithecus*) [2]	-	(N) 17<br>(M) 33,4<br>±3,6		(N) 17<br>(M) 19,5<br>±2,6		-	-	-	-	-	-		-
Early Pleist. (Early *Homo*) [2]	-	(N) 11<br>(M) 31,4<br>±4,3		(N)12<br>(M) 20,2<br>±2,9		-	-	-	-	P3-P4	Single		-
Early Pleist. (*H.*ergaster/*erectus*) [2,4]	(N) 4<br>(M) 31,2	(N) 11<br>(M) 31<br>±3,3		(N) 11<br>(M) 19,7<br>±1,5		(N) 5<br>(M) 19,3	(N) 9<br>(M) 20,3	-	-	-	-		-
Middle Pleist. (*H.erectus* Nord Africa) [2,4]	(N) 4<br>(M) 32,9	(N) 4<br>(M) 33,6<br>±5.2		(N) 4<br>(M) 18,1<br>±1,7		(N) 5<br>(M) 17,6	(N) 7<br>(M) 17,4	-	-	-	-		-
African archaic Middle Pleist.[3]	(N) 6<br>(M) 31,7<br>±4.10	(N) 7<br>(M) 30,8		(N) 7		(N) 6	(N) 8	(N) 8<br>(M) 19,47	-	-	-		(N) 5

Chin and Symphysis (Measurements in mm)													
**Sample/specimens**	**H. Sy**	**H. MF**		**Th. MF**		**Th. Sy**	**Th. M1**	**Th. M2**	**Th. M3**	**MF Loc. main foramen**	**MF number (N) & size** (long x high)**		**Bi-canine breadth**
		right	left	right	left						right	left	
		± 5,21		(M) 17,96<br>± 1,78		(M)17,32<br>± 2,57	(M) 18,03<br>±2,46	±<br>2,52					(M) 34,20<br>±5,25
**Europe**													
Lower Pleistocene [5]													
ATE 9-1	33,5	30		-		15,3	-	-	-	-	-		-
ATD 6-5	-	-		-		-	-	17,9	-	-	-		-
ATD 6-113	-	-		-		-	-	20	-	-	-		-
Archaic MiddlePleist.[3]	(N) 4<br>(M) 31<br>± 2,98	(N) 4<br>(M) 30,2<br>± 2,34		(N) 4<br>(M) 18,5<br>± 2,67		(N) 4<br>(M) 16,88<br>± 2.06	(N) 4<br>(M) 18,08 ±2.76	(N) 4<br>(M)19,05<br>± 3,68	-	-	-		(N) 3<br>(M) 36,73 ±1,17
SH[5]	(N) 10<br>(M)33<br>± 4	(N)12<br>(M) 32,6<br>± 3,5		(N) 12<br>(M) 17<br>± 1,9		-	-	(N)<br>13<br>(M) 17,6<br>± 1,5	-	-	Single		-
SH 1	31,4	29,9		17,6		-	-	18,3	-	M1	-		-
SH 4	36,2	31		17,4		-	-	17,5	-	M1	-		-
SH 5	29,3	31,1		16,6		-	-	16,6	-	M1	-		-
SH 6						-	-	16,7	-	-	-		-
SH 7		37,8		21,8		-	-	20,8	-	M1	-		-
SH 12	33,8	33,7		18,2		-	-	19,7	-	P4-M1	-		-
SH 15	32,3	31,5		15,2		-	-	15,9	-	P4-M1	-		-
SH 19	27	28,6		14.6		-	-	16,7	-	P4-M1	-		-
SH 21	36	35,5		16,7		-	-	16,9	-	M1	-		-
SH 22	34,7	36,5		17		-	-	18,2	-	M1	-		-
SH 26		29,3		16,7		-	-	17,2	-	M1	-		-
SH 27	40,3	38		17,8		-	-	18,1	-	M1	-		-
SH 28	29,4	28,8		14,8		-	-	15,6	-	M1	-		-
Montmaurin-LN [6]	-	(N) 1<br>28,5<br>(M1 level)		-		-	16	-	-	-	Multiple		-

Chin and Symphysis (Measurements in mm)

Sample/specimens	H. Sy	H. MF		Th. MF		Th. Sy	Th. M1	Th. M2	Th. M3	MF Loc. main foramen	MF number (N) & size** (long x high)		Bi-canine breadth
		right	left	right	left						right	left	
Middle Pleist.(5)	(N) 7 (M) 33,3 ± 4,6	(N)6 (M) 32,9 ± 3,9		(N) 8 (M) 17,6 ± 2,2		(N) 6 (M) 17 ± 2,3	-	(N) 8 (M) 18,5 ± 2,7	-	P4-M1	Single		
Asian Nea (3)	(N) 6 (M) **36,10** ±3,36	(N) 7 (M) 33,90 ± 3,51		(N) 7 (M) 17,16 ± 1,89		(N) 5 (M) 17,2 ± 3,92	(N) 5 (M) 17,54 ± 2,67	(N) 3 (M) 17,60 ± 1,54	-	-	-		(N) 6 (M) 36,48 ± 1,63
Europ. Nea (3)	(N) 26 (M) 34 ± 4,59	(N) 43 (M) 31,22 ± 3,64		(N) 43 (M) 15,67 ± 1,56		(N) 26 (M) 15,37 ± 1,72	(N) 27 (M) 16,53 ± 1,66	(N) 35 (M) 16,33 ± 1,62	-	-	-		(N) 18 (M) 36,47 ± 2,41
Nea(5)	(N) 19 (M) 34,6 ± 4,7	(N) 22 (M) 32,3 ± 3,3		(N) 22 (M) 15,6 ± 1,6		(N) 17 (M) 15,8 ± 2,4	-	(N) 18 (M) 16,1 ±1,98	-	-	Multiple		(N) 18 (M) 35,4 ±2,64
Oase 1 Muierii -7	-	-		(N)1 11,6		-	-	-	-	P4	Single		-
Early *H. Sapiens* (3)	(N) 9 (M) **37,3** ± 6,33	(N) 12 (M) 34,90 ± 4,33		(N) 12 (M) 16,28 ± 1,47		(N) 13 (M)15,56 ± 2,25	(N) 12 (M) 17,2 ± 2,45	(N) 9 (M) 8,9 ± 3,08	-	-	-		(N) 6 (M) 38,08 ± 1,80
UP *H. sapiens* -3	(N) 38 (M) 31,9 ± 2,82	(N) 42 (M) 30,93 ± 3,26		(N) 43 (M) 12,74 ± 1,60		(N) 38 (M) 15,29 ± 2	(N) 26 (M) 14,25 ±1,57	(N) 36 (M) 15,02 ±1,89	-	-	-		(N) 28 (M) 32,64 ±2,38
LP east Asian humans (3)	(N) 13 (M) 30,3 ± 4,81	(N) 13 (M) 30,09 ± 3,18		(N) 13 (M) 13,09 ± 1,68		(N) 12 (M) 13,91 ± 1,8	(N) 12 (M) 13,54 ± 2,07	(N) 12 (M) 14,89 ± 2,38	-	-	-		(N) 11 (M) 31,75 ± 3,22

Chin and Symphysis (Measurements in mm)													
**Sample/specimens**	**H. Sy**	**H. MF**		**Th. MF**		**Th. Sy**	**Th. M1**	**Th. M2**	**Th. M3**	**MF Loc. main foramen**	**MF number (N) & size** (long x high)**		**Bi-canine breadth**
		right	left	right	left						right	left	
MH(5)	(N) 76 (M) 31,1 ± 3,3	(N) 77 (M) 30,2 ± 3,1		(N) 77 (M) 12,1 ± 1,7		(N) 78 (M) 14,8 ±1,7	-	(N) 74 (M) 15,3 ±1,6	-	-	-		(N) 37 (M) 34,1 ±3,08

Data does include few individual values and it was not possible to include it in statistical analyses, therefore we only employed it for comparative purposes.

*Bone atrophy; **The value refers to the main *foramina*

Comparison data was taken from *Refs*.: [1][129]; [2][127]; [3][126]; [4][128]; [5][130]; [6][70]; [7][138]

Table S10. Circeo dental sample compared with other specimens of Pleistocene. **Montmaurin-LN** (Montmaurin- La Niche); **BH-1**(Mala Balanica); ***Hl***, (*Homo luzonensis*). **MD**, Mesio-Distal; **BL**, Bucco-Lingual; **DB**, Disto-Buccal; **B**, Buccal; **Mr**, Mesial root; **Dr**, Distal root; **MLr**, Mesio-Lingual root; **Cr**, Crown; **C**, Cervical; **Mr**, Middle-root; **Ra**, Root apex. **M** (Mean); **ND**, No Detectable.

**Superior Maxillary** (Measurements in mm)					
**Sample/Specimens (*Genus*)**	**Tooth**	**MD (M81)**	**BL [M81 (1)]**	**Maximum root height**	***References***
**Circeo13**	**C^1**	**8,4** Cr	**9,2**	**22,6**	Present study
	**31,7** Max height	**5,8** C	**9,5**		
		**5** Mr	**9**		
		**4,5** Ra	**6,1**		
**Circeo14**	**P^4**	**6.8**	**9.2**	**Hypercementosis**	Present study
**Circeo15**	**M^1**	**~ 8,0**	**~ 10,4**	**ND**	Present study
**Circeo10 a-b**	**M^2 a**	**10,1**	**11,9**	**17,1** DB	Present study
	**M^3 b**	**9,5**	**11,8**	**15,2** B	
Homo habilis					
**Ethiopia**					
**(**Hadar)					
A.L. 666-1	^{1}C	-10,3	-	-	[139]
	^{4}P	8,3/9	12,6	-	
	^{1}M	dic-13	-	-	
	^{2}M	12,4/13,5	14,4	-	
	C^1	-10,4	10,2	-	
	P^4	8,4/9	12,6	-	
	M^1	11,9/12,9	12,4	-	
**Tanzania**					
**Western Olduvai Gorge**					
OH 65	^{1}C	9,7	9,4	-	[140, 141]
	C^1	9,6	9,9	-	

Superior Maxillary (Measurements in mm)					
**Sample/Specimens (*Genus*)**	**Tooth**	**MD (M81)**	**BL [M81 (1)]**	**Maximum root height**	***References***
	^{4}P	9	13,1	-	
	P^{4}	9,1	13	-	
	^{1}M	13	13,6	-	
	M^{1}	12,5	13,5	-	
	^{2}M	12,8	14,4	-	
	M^{2}	13,4	14,3	-	
	^{3}M	11	14	-	
	M^{3}	11,5	14	-	
***Homo erectus* Middle Pleistocene**					
**China**					
Yiyuan	^{4}P	8,2	12,3	-	[142]
~420 000-320 000 BP	^{4}P	-7,9	11,2	-	
	M^{2}	-11,6	14,3	-	
Hexian (PA836)	^{1}M	12,3	13,7		[143]
Hexian (PA837)	M^{2}	12,5	15,5		
Hexian (PA833)	^{2}M	12	14		
**Late *Homo erectus***					
**Southeast Asia**					
Hl CCH6	P^{4}	6,6	10,2	-	[73]
Hl CCH8	P^{4}/P^{3}	6,8	10,1	-	
Hl CCH6	M^{1}	8,6	10,1	-	
Hl CCH6	M^{2}	8	10,5	-	
Hl CCH6	M^{3}	7,5	9,7	-	
Hl CCH9	M^{3}	7,2	9,7	-	
**South Africa**					
**Homo naledi**					
LES1	C^{1}	7,5	8,7	-	[144]
	P^{4}	8,1	11,3	-	
	M^{1}	-10,6	11,8	-	
	M^{2}	11,7	12,7	-	
	M^{3}	11,4	12,7	-	
**Europe**					
**Proto-Neanderthal**					
**France**					
Biache-Saint-Vaast	P^{4}	7,9	10,6	-	[131]
(BSV)	M^{1}	11,7	12,5	-	
	M^{2}	11	12	-	
	M^{3}	9,6	11,5	-	
Arago 26	P^{4}	(N1) 8,6	11,4	-	[131, 132]
Arago 9, 21, 54	M^{1} (M)	(N3) 11,8±1,1	13,4±1	-	
Arago 14,21,68	^{2}M-M^{2}- M^{2} (M)	(N3) 12±0,6	14,5±1,4	-	

**Superior Maxillary** (Measurements in mm)					
**Sample/Specimens (*Genus*)**	**Tooth**	**MD (M81)**	**BL [M81 (1)]**	**Maximum root height**	***References***
Arago 21	^{3}M-M^{3}	(N2) 9,6	12,3	-	
**Spain**					
Atapuerca-SH					
(Sima de los Huesos)					
AT-1130 + AT-2194	^{4}P	7,2	9,2	-	[133]
	^{1}M	9,9	11,4	-	
	M^{1}	9,9	11,4	-	
	^{2}M	9	11,9	-	
	M^{2}	9	11,9	-	
AT-144	C^{1}	9,6	10,4	-	
AT-2759	C^{1}	8,4	9,7	-	
AT-163	^{1}C	9,6	10,4	-	
AT-3191	C^{1}	8,8	10,1	-	
AT-818	^{1}C	8,3	8,7	-	
AT-219	C^{1}	8,1	8,8	-	
AT-3075	^{1}C	8,7	10	-	
AT-3255	C^{1}	8,5	10	-	
AT-6	^{1}C	8,5	10	-	
AT-44	^{1}C	8,3	9,1	-	
AT-825	^{1}C	8,5	9,9	-	
AT-958	^{1}C	8,4	9,1	-	
AT-1475	C^{1}	8,7	10,2	-	
AT-1757	^{1}C	8,4	9,5	-	
AT-1758	C^{1}	8,5	9,1	-	
AT-1942	^{1}C	8,8	9,8	-	
AT-3192	C^{1}	-	10	-	
AT-5622	^{1}C	8,7	9,8	-	
AT-5616	C^{1}	8,8	10,2	-	
AT-940	P^{4}	7,2	10,4	-	
AT-23	^{4}P	8,2	11,4	-	
AT-193	P^{4}	8	11,2	-	
AT-4151	^{4}P	7,2	10	-	
AT-4156	P^{4}	7,5	9,8	-	
AT-2719	^{4}P	-	9,6	-	
AT-3189	^{4}P	7,4	10,1	-	
AT-4323	P^{4}	7,2	10,2	-	
AT-4656	^{4}P	7,4	10,3	-	
AT-409	^{4}P	8,8	11,5	-	
AT-5899	^{4}P	7,1	9,5	-	
AT-5836	P^{4}	-	10,3	-	
AT-279	P^{4}	7,7	11,1	-	
AT-4324	P^{4}	7	10	-	
AT-5510	P^{4}	7	9,1	-	
AT-68	P^{4}	7,3	9,9	-	
AT-746	P^{4}	7,4	10	-	

**Superior Maxillary** (Measurements in mm)					
**Sample/Specimens (*Genus*)**	**Tooth**	**MD (M81)**	**BL [M81 (1)]**	**Maximum root height**	***References***
AT-1100 + AT-1111 (15 - 17y old)	^{4}P	7,1	9,9	-	
	P^{4}	7,1	9,8	-	
AT-559 (17 – 19y old)	^{4}P	8,1	10,6	-	
AT-3184 (12,5 - 14,5y old)	^{4}P	7,1	9,6	-	
AT-949 (12,5 - 14,5y old)	P^{4}	7,2	9,5	-	
AT-2070 (9.5 - 11.5y old)	^{4}P	8,3	10,9	-	
AT-2189 (9.5 - 11.5y old)	P^{4}	8,3	11,3	-	
AT-405 (12,5 - 14,5y old)	^{4}P	8,4	10,9	-	
AT-806 (12,5 - 14,5y old)	^{4}P	8	9,2	-	
AT-3188 (12,5 - 14,5y old)	P^{4}	7,9	9,2	-	
AT-4317	^{1}M	11,7	11,5	-	
AT-944	M^{1}	11,5	11,4	-	
AT-16	^{1}M	11,8	12,3	-	
AT-139	M^{1}	11,5	12,6	-	
AT-3424	M^{1}	10,2	11,6	-	
AT-20	M^{1}	10,7	11,9	-	
AT-2770	^{1}M	10,2	11,4	-	
AT-3178	M^{1}	10,9	11,5	-	
AT-4319	^{2}M	9,5	12,2	-	
AT-588	M^{2}	9,3	12,3	-	
AT-270	^{2}M	10,9	13,3	-	
AT-46	M^{2}	10,6	13,4	-	
AT-4336	^{2}M	10,7	12,1	-	
AT-822	M^{2}	10,2	12,6	-	
AT-810	^{2}M	8,5	11,6	-	
AT-4326	M^{2}	8,3	11,9	-	
AT-824	M^{2}	10,8	12	-	
AT-12	M^{2}	9,7	11,3	-	
AT-772	P^{4}	7,2	10,3	-	
	M^{2}	10,5	12,5	-	
	M^{1}	10,3	11	-	
AT-700	^{1}M	11,5	10,4	-	
	^{2}M	8,3	11,5	-	
	M^{2}	8,9	11,5	-	
	^{3}M	8,7	10,8	-	
	M^{3}	9	11,2	-	
AT-2771	^{3}M	8,8	11,8	-	
AT-948	M^{3}	8,6	11,8	-	
AT-3180	^{3}M	9,6	12,7	-	
AT-819	M^{3}	9,2	12,3	-	
AT-951	^{3}M	9	12,5	-	
AT-140	^{3}M	9,3	13	-	
AT-4102	M^{3}	8	10,6	-	
AT-1471	M^{3}	7,7	10	-	
AT-2393	M^{3}	8,9	11,1	-	

**Superior Maxillary** (Measurements in mm)					
**Sample/Specimens (*Genus*)**	**Tooth**	**MD (M81)**	**BL [M81 (1)]**	**Maximum root height**	***References***
AT-602	^{3}M	8,3	11,8	-	
AT-601	M^{3}	7,7	12	-	
AT-945	M^{3}	9,1	11	-	
AT-3181	^{3}M	8,6	11,5	-	
AT-3183	M^{3}	8,2	10,3	-	
AT-274	^{3}M	8	10,1	-	
AT-805	M^{3}	9,3	12,3	-	
AT-5082	M^{3}	8,7	12	-	
AT-10	M^{3}	8,6	11,5	-	
Atapuerca-SH				-	
	C1 (M)	(N23) 8,75±0,38	(N24) 9,88±0,52		[133]
	P4 (M)	(N24) 7,55±0,53	(N26) 10,27±0,65	-	
	M1 (M)	(N26) 11,05±0,68	(N26) 11,5±0,62	-	
	M2 (M)	(N28) 9,94±0,94	(N28) 12,18±0,73	-	
	M3 (M)	(N24) 8,62±0,6	(N25) 11,46±0,92	-	
**Neanderthal**					
**Italy**					
Fate F13	M^{1}	11,9	12,2	-	[145]
**Croatia**					
Krapina 144	^{1}C	10	11,4	-	[146, 147]
Krapina 146	^{1}C	8,2	9,9	-	
Krapina F/H	^{1}C	9,3	10	-	
Krapina F/H	C^{1}	8,8	9,9	-	
Krapina N/N	C^{1}	8,5	11,1	-	
Krapina F/H	^{4}P	7,6	10,5	-	
Krapina 40	^{4}P	8,2	-	-	
Krapina 42 (14y old)	P^{4}	7,4	10,5	-	
Krapina 43 (17y old)	P^{4}	8,7	11,3	-	
Krapina 44 (12y old)	P^{4}	8,1	10,8	-	
Krapina 52 (16y old)	^{4}P	8,3	11,4	-	
Krapina D/D (15y old)	^{4}P	8,4	11,2	-	
Krapina MX E (16y old)	^{4}P	8,7	10,6	-	
Krapina MX J (16y old)	^{4}P	8,8	11,7	-	
Krapina MX M (17y old)	P^{4}	8,5	11	-	
Krapina N/N	P^{4}	6,8	10,4	-	
Krapina F/H	^{1}M	11,9	12,1	-	
Krapina F/H	^{2}M	10,4	12,6	-	
Krapina 172	M^{2}	10	12	-	
Krapina MX P	^{2}M	10,4	13,3	-	
Krapina F/H	^{3}M	9,8	11,7	-	
Krapina MX P	^{3}M	11	-	-	
Krapina MX P	M^{3}	10,6	-	-	
Krapina	P^{4} (M)	(N11) 8,1±0,6	10,9±0,4	-	[131]
	M^{1} (M)	(N9) 12,4±0,7	12,6±0,9	-	

**Superior Maxillary** (Measurements in mm)					
**Sample/Specimens (*Genus*)**	**Tooth**	**MD (M81)**	**BL [M81 (1)]**	**Maximum root height**	***References***
	M^2 (M)	(N10) 11,3±1,1	12,8±0,8	-	
	M^3 (M)	(N9) 10,4±0,6	12,5±0,6	-	
**France**					
La Chaise de Vouthon					
Abri Bourgeois Delaunay BD11	C1	8	9,2	-	[148]
Abri Bourgeois Delaunay BD16	C1	7,9	9,5	-	
Abri Bourgeois Delaunay BD15	C1	7,9	9	-	
Grotte du Bison (Arcy sur Cure)	C^1	8,3	9	-	[149]
P1 1.8	P^4	6,5	10,1	-	
Grotte Boccard GB 77 C13 C65 3	P^4	7,34	10,68	-	[150]
La Ferrassie 11 ULC / LF11	${}^1C$	>8	9,9	-	[151]
La Ferrassie 7 URP4 / LF7	P^4	7,4	10,4	-	
La Quina 5	${}^1C$	8,35	10,37	-	[152]
	C^1	8,94	10,53	-	
	${}^4P$	7,82	10,53	-	
	P^4	7,78	11,11	-	
	${}^1M$	10,62	12,71	-	
	M^1	10,54	12,66	-	
	${}^2M$	10,05	13,97	-	
	M^2	9,96	10,54	-	
	${}^3M$	9,08	14,25	-	
	M^3	8,98	12,66	-	
La Quina 20c	M^2	11,66	13,52	-	
La Quina 22	M^2	10,73	13,44	-	
La Quina 20b	M^3	9,28	11,53	-	
La Chaise de Vouthon					
Abri Bourgeois Delaunay BD14 or I 8/20	${}^4P$	6,8	9,3	-	[148]
La Chaise de Vouthon	${}^1M$	12,5	11,9	-	
Abri Bourgeois Delaunay	${}^2M$	11,4	12,3	-	
BD8	${}^3M$	9,3	11,8	-	
Grotte du Bison					
(Arcy sur Cure) P11.8	M^1	9,3	12,3	-	[149]
P11.8	M^2	9,2	12,1	-	
P11.8	M^3	9,9	12,5	-	
P8-63	M^3	8,6	11,6	-	

Superior Maxillary (Measurements in mm)					
**Sample/Specimens (*Genus*)**	**Tooth**	**MD (M81)**	**BL [M81 (1)]**	**Maximum root height**	***References***
Beau de l'Aubesier Aubesier 9					
Aubesier 9	M^1 or M^2	-	12,2	-	[153]
Aubesier 10	M^1 or M^2	-	12	-	
Monsempron					
Maxillaire h	C^1	9	9,5	-	[154]
	P^4	8	10,5	-	
	M^2	11	12	-	
	M^2	10	13	-	
Montmaurin					
Coupe gorge cave					
C.G. 10B3T, n°10.314	C^1	9,3	9,6	-	[155]
C.G. 2F3	^1C	9,1	8,9	-	
14 B3S, n°14.188	P^4	8	10,9	-	
C.G. 6B3	^4P	10,9	-	-	
				-	
Moula-Guercy					
M-I4-TNN3	C^1	8,6	9,5	-	[156]
M-I4-55	M^1	11,4	11,1	-	
M-F3-215	M^2	10,6	11,6	-	
M-G2-117	M^2	10,4	12	-	
Marillac					
M13E	C^1	8,63 Cr	9,56	-	[157]
		6,3 C	8,9		
M13G (14-16y old)	^4P	7,6	10,18	-	
M20	^2M	> 11	-9,71	-	
Soulabé-Las-Maretas	^3M	10,37	12,41	-	[158]
**Germany**					
Neanderthal NN51	P^4	6,6	9	-	[159]
Neanderthal NN31	M^2	10,5	13,1	-	
Neanderthal NN16	^2M	10,7	13,3	-	
Neanderthal NN33	^3M	10,5	12,7	-	
Hunas	M^3	11,92	10,78	-	[160]
**Greece**					
Kalamakia	^4P	5,7	-9,8	-	[161]
KAL5					
KAL8	M^2	-8,6	11,7	-	
KAL3	(M^3)	10,3	14,1	-	
**Iraq**					
Shanidar 1	C^1	-	-9,5	-	[58]
	^1C	-	-10	-	

Superior Maxillary (Measurements in mm)					
**Sample/Specimens (*Genus*)**	**Tooth**	**MD (M81)**	**BL [M81 (1)]**	**Maximum root height**	***References***
	P^4	6,8	9,7	-	
	^4P	-6,2	10	-	
	M^1	10,3	12,3	-	
	^1M	10,7	11,7	-	
	M^2	9,1	11,8	-	
	^2M	10,1	11,9	-	
	M^3	9,7	11,6	-	
	^3M	9,4	11,6	-	
Shanidar 2	C^1	7,6	9,8	-	
	^1C	8	9,9	-	
	P^4	6,1	10	-	
	^4P	7	10,3	-	
	M^1	10,7	12,1	-	
	^1M	10,9	12,3	-	
	M^2	10,5	12,9	-	
	^2M	11,3	12,7	-	
	M^3	10	12,9	-	
	^3M	9,7	12,3	-	
Shanidar 3	^1C	-7,3	9,8	-	
	M^3	9,6	12,8	-	
Shanidar 4	^1C	-	-9,9	-	
Shanidar 5	^1M	-	13,4	-	
	^3M	9,6	13	-	
Shanidar 6	C^1	7,4	10,3	-	
	P^4	6,5	10,5	-	
	^4P	7,4	10,7	-	
	M^1	10,7	12,1	-	
	^1M	11	12,3	-	
	M^2	11,4	12,6	-	
	^2M	11,8	12,8	-	
	M^3	10,6	12,2	-	
	^3M	10,6	13	-	
**Israël**					
Amud 1	C^1	8,5	9,5	-	[162]
	^1C	8,5	9,5	-	
	P^4	6,6	10,1	-	
	^4P	6,6	10	-	
	M^1	10,7	12,4	-	
	^1M	10,7	12,5	-	
	M^2	10,4	12,3	-	
	^2M	10,4	12,2	-	
	M^3	6,8	7,7	-	
	^3M	8,5	11	-	
Hayonim cave					
HCpM1	M^2	9,54	12,36	-	[163]

**Superior Maxillary** (Measurements in mm)					
**Sample/Specimens (*Genus*)**	**Tooth**	**MD (M81)**	**BL [M81 (1)]**	**Maximum root height**	***References***
**Poland**					
Stajnia Cave	M^2	9,6 (10,72)	11	-	[164]
S5000					
**Spain**					
El Salt 3	P^4	7,5	-10,3	-	[165]
Palomas 1	C^1	-8,9	9,8	-	[166, 167]
	^1C	-8,4	10,3	-	
	P^4	-6	9,9	-	
	^4P	-5,3	9,5	-	
	M^1	-10,2	11,2	-	
	^1M	-10,1	11,4	-	
	M^2	-10,2	12	-	
	^2M	-9,4	11,9	-	
	M^3	10	11,5	-	
	^3M	-8,6	11	-	
Palomas 35	^1C	7,9	8,4	-	
Palomas 51	M^3	8,5	10,5	-	
Palomas 68	P^4	-	9	-	
Palomas 95	^4P	7,7	10,5	-	
**United Kingdom**					
Pontnewydd					
PN4	M^1	12,2	12,4	-	[168]
PN12	^1M	11,3	11,1	-	
PN1	^2M	11,6	13,3	-	
PN17	M^3	9.4	12,3	-	
PN19	M^3	9,5	11,4	-	
**Uzbekistan**					
Obi-Rakhmat Grotto					
OR-1	^4P	8,1	11,1	-	[169]
Neanderthal	P^4 (M)	(N20) 7,1±0,5	9,9±0,6	-	[131]
	M^1 (M)	(22) 11,1±0,8	11,9±0,4	-	
	M2 (M)	(N19) 10,5±0.8	12,3±1,2	-	
	M3 (M)	(N16) 9,6±0,7	12±1	-	
**Denisovian**					
**Russia** (Altai mountain)					
Denisova 4	M^2/ M^3	13,1	14,7	-	[104]
Denisova 8	M^3	14,3	14,65	-	
Homo sapiens					
**Israël**					

Superior Maxillary (Measurements in mm)					
**Sample/Specimens (*Genus*)**	**Tooth**	**MD (M81)**	**BL [M81 (1)]**	**Maximum root height**	***References***
Misliya 1	^{1}C	8,1	9	-	[170]
	^{4}P	6,8	9,1	-	
	^{1}M	10,4	12,3	-	
	^{2}M	9,7	12,3	-	
	^{3}M	8,9	11,8	-	
Qafzeh 5	P^{4}	6,5	9,4	-	[171]
	^{4}P	6,6	9,5	-	
Qafzeh 6	C^{1}	8,5	10	-	
	^{1}C	8,5	10	-	
	P^{4}	6.5	-	-	
	^{4}P	-	10	-	
	M^{1}	11	12,5	-	
	^{1}M	11	13	-	
	M^{2}	10,5	13	-	
	^{2}M	10,5	13	-	
Qafzeh 7	C^{1}	8	9,5	-	
	^{1}C	8,5	-	-	
	P^{4}	7	10,6	-	
	^{4}P	6,9	-	-	
	M^{1}	11,1	12,9	-	
	^{1}M	11,1	-	-	
	M^{2}	10,5	12,6	-	
	^{2}M	10,2	-	-	
	M^{3}	9,4	12,1	-	
	^{3}M	9,3	-	-	
Qafzeh 8	C^{1}	8	8,5	-	
	P^{4}	6,6	10,3	-	
	M^{1}	11,2	13,2	-	
Qafzeh 9	C^{1}	9,7	10,2	-	
	^{1}C	9,4	10,3	-	
	P^{4}	7,6	10,7	-	
	^{4}P	7,6	10,9	-	
	M^{1}	12	13,1	-	
	^{1}M	11,7	13,3	-	
	M^{2}	11,6	12,2	-	
	^{2}M	11,8	12,2	-	
	M^{3}	10,9	12,1	-	
	^{3}M	9,5	13,5	-	
Tabun BC7	M^{1}	11,2	12,5	-	
	M2	9	13,2	-	[172]
	M3	9,1	11,5	-	
**Morocco**					
Djebel Irhoud 21	^{1}C	8,9	9,8	-	[173]
	^{4}P	8,3	11,3	-	

**Superior Maxillary** (Measurements in mm)					
**Sample/Specimens (*Genus*)**	**Tooth**	**MD (M81)**	**BL [M81 (1)]**	**Maximum root height**	***References***
	^{1}M	12,2	12,7	-	
	^{2}M	11,8	12,4	-	
	^{3}M	8,4	12,4	-	
Djebel Irhoud 10	^{1}M	12,7	-12,1	-	
	^{2}M	12,3	10,9	-	
Djebel Irhoud 22	M^{2}	12,6	13,8	-	
	M^{3}	11,6	13,3	-	
**Syria**					
Dederiyeh cave Dederiyeh-8904					
	^{4}P	6,7	9	-	Not known
Dederiyeh-9011	M^{3}	8,3	10,3	-	

**Inferior Maxillary** (Measurements in mm)					
**Sample/Specimens (*Genus*)**	**Tooth**	**MD (M81)**	**BL [M81 (1)]**	**Maximum root height**	***References***
**Circeo11**	$\mathbf{M_{3}}$	**11,8**	**10,5**	**Hypercementosis**	Present study
					Present study
**Circeo12**	$\mathbf{_{3}M}$	**11,3**	**9,6**	**15,3 Dr 16,4 Mr**	
C2	M_{3}	11,2	10,5	-	Original data
C3	M_{3}	11,1	10,1	-	
Paranthropus boisei					
**Turkana (Kenia)**					
KNM-ER 729A	M_{3}	22,2	19	-	[174]
	$_{3}M$	21,2	19	-	
KNM-ER 730	$_{3}M$	13,7	11,5	-	
KNM-ER 801A	M_{3}	19,2	16		
KNM-ER 802F	M_{3}	18.7	16.4		
KNM-ER 806A (*H. erectus*)	$_{3}M$	14,9	12,4		
KNM-ER 806D	M_{3}	14,4	12,3		
KNM-ER 810B	$_{3}M$	17,7	15,7		
KNM-ER 818	$_{3}M$	21,9	18,2		
KNM-ER 992A (*H. erectus*)	M_{3}	13,2	12,1		
KNM-ER 992B	$_{3}M$	13,4	12,3		
KNM-ER 1462	$_{3}M$	14,4	13,3		
KNM-ER 1467	$_{3}M$	18,7	15,4		
KNM-ER 1480	M_{3}	15,4	12,5		
KNM-ER 1509A	$_{3}M$	19,8	15,9		
KNM-ER 1801	$_{3}M$	17	14,6		
KNM-ER 1805C	M_{3}	15	12,2		
KNM-ER 1812C (*H. erectus*)	$_{3}M$	14,5	12,5		
KNM-ER 2601	M_{3}	13,5	10,4		
KNM-ER 3230	M_{3}	21,3	16,9		
	$_{3}M$	20,5	16,5		

Inferior Maxillary (Measurements in mm)					
**Sample/Specimens (*Genus*)**	**Tooth**	**MD (M81)**	**BL [M81 (1)]**	**Maximum root height**	***References***
KNM-ER 3953	M_3	15,4	12,5		
KNM-ER 5877	M_3	25,5	20		
KNM-ER 15930	$_3M$	18,2	15		
KNM-ER 15950	$_3M$	20	17		
KNM-ER 15940	M_3	18,5	15,9		
	$_3M$	18	15,5		
KNM-ER 64060	$_3M$	14,8	13,3		
KNM-WT 17396	$_3M$	19	17		
KNM-BK 67	M_3	12,8	11,2		[174,175 Taxonomic reassessment]
Homo sp. indet. (aff. *erectus*)					
KNM-BK 67	$_3M$	12,9	11,4		
Homo sp. indet. (aff. *erectus*)					
**Omo Ethiopia**					
Omo L2-89	$_3M$	13,7	11,5		[174]
Omo L7A-125	M_3	18,2	14,8		
Omo L338x-39	$_3M$	19,4	15,1		
Omo L398-630	M_3	17,7	14,7		
Omo L628-2	$_3M$	19	17,8		
Omo L628-3	$_3M$	18,7	16,2		
Omo L33-9	M_3	19,3	15,1		
Omo L75-14a	$_3M$	15,1	14,1		
Omo L136-1	$_3M$	17,9	15,6		
Omo L136-2	$_3M$	16,7	14,6		
Omo F22-1b	M_3	20,2	18		
Omo F203-1	M_3	17,2	15,9		
Paranthropus robustus					
**Swartkrans (South Africa)**					[174]
SK6	M_3	18,4	16,2		
	$_3M$	18,7	15,5		
SK12	$_3M$	17,3	15,3		
SK23	M_3	17,5	14,4		
	$_3M$	16,8	13,1		
SK34	M_3	18,2	17		
	$_3M$	18,1	16		
SK75	M_3	17,5	15		
SK81	$_3M$	17,4	14,8		
SK840	$_3M$	16,4	12,9		
SK841b	$_3M$	15,9	13,8		
SK843	$_3M$	17,5	15,1		

**Inferior Maxillary** (Measurements in mm)					
**Sample/Specimens (*Genus*)**	**Tooth**	**MD (M81)**	**BL [M81 (1)]**	**Maximum root height**	***References***
SK844	$_3M$	16	14		
SK858	M_3	17,5	-		
SK880	$_3M$	17,9	14,5		
SK885	$_3M$	15,5	14		
SK1586	M_3	16,3	15,1		
**Kromdraai**					
TM 1518	M_3	16,8	15		
TM 1519	M_3	15,7	14		
TM 1520	$_3M$	16,9	14,1		
TM 1517	M_3	16,4	14,3		
TM 1600	$_3M$	16,1	14,9		
Australopithecus africanus					
**Sterkfontein**					
Sts 7	$_3M$	16,4	14,4		
Sts 52b	M_3	13,7	12,8		
	$_3M$	13,7	13		
Sts 55b	$_3M$	15,5	13,7		
Stw/H14	M_3	17,7	14,7		
**Makapansgat**					
Peninj	M_3	18,8	15,7		
Peninj	$_3M$	18,2	16,1		
MLD 4	$_3M$	-	14,2		
MLD 18	M_3	14,2	13,9		
MLD 19	$_3M$	15,1	13,6		
Homo habilis					
**Tanzania**					
**Western Olduvai Gorge**					
OH 4	$_3M$	15,4	13		
OH 13	M_3	14,8	12,4		
	$_3M$	14,8	12,3		
OH 16	M_3	15,9	14,4		
	$_3M$	15,9	14,3		
OH 27	M_3	15,4	13,3		
Omo					
Homo erectus					
**Southeast Asia**					
Sangiran 21	M_3	12,2 (12,3)	10,9	-	[129]
Sangiran 8	M_3	14,6 (15,1)	13	-	
Sangiran 9	M_3	13,1 (13,8)	12,7	-	
***Homo erectus* Middle Pleistocene**					
**Eastern China**					
Hexian (PA 831)	$_3M$	11,2	10,8	-	[127]
Hexian (PA 834-2)	$_3M$	13,6	13,6	-	

**Inferior Maxillary** (Measurements in mm)					
**Sample/Specimens (*Genus*)**	**Tooth**	**MD (M81)**	**BL [M81 (1)]**	**Maximum root height**	***References***
Homo erectus/Homo heidelbergensis					
**Northwestern Algeria**					
Tighennif 1	M_3	12,4	12,2	-	[176]
	$_3M$	12,4	12,2		
Tighennif 2	$_3M$	13,2	12,7	-	
Tighennif 3	M_3	12,3	11,5	-	
	$_3M$	12	11		
**Germany**					
Mauer	M_3	12,2	10,9	-	[70, 177]
	$_3M$	11,5	11,3	-	
**Europe**					
**Lower Pleistocene**					
**Spain**					
Gran Dolina-TD6					
H7	$_3M$	9,2	8,8	-	[176]
H10	$_3M$	12,5	10,6	-	
**South Africa**					
**Homo naledi**					
LES1	M_3	13,3	11,7	-	[144]
	$_3M$	13,3	11,7	-	
**Europe**					
**Proto-Neanderthal**					
**France**					
Montmaurin-LN	M_3	12,8	10,3	-	[70]
	$_3M$	12,7	10,5		
BH-1	M3	12,1	10,5	-	
Arago2	M3	10,5	9,7	-	
Arago13	M3	13,2	12,5	-	
**Spain**					
Atapuerca-SH (Sima de los Huesos)					
AT-13	$_3M$	12,7	11,3	-	[133]
AT-605	$_3M$	12	10,6	-	
	M_3	11,8	10,7	-	
AT-792	$_3M$	12,9	10,6	-	
	M_3	12,7	10,3	-	
AT-811	$_3M$	10,9	9,8	-	
AT-100	M_3	10,9	10	-	
AT-598	$_3M$	11,7	9,8	-	
AT-1468	M_3	11,6	9,6	-	
AT-1473	$_3M$	11,7	9,4	-	

Inferior Maxillary (Measurements in mm)					
**Sample/Specimens (*Genus*)**	**Tooth**	**MD (M81)**	**BL [M81 (1)]**	**Maximum root height**	***References***
AT-143	M_3	11,7	10,1	-	
AT-2777	M_3	12,1	11	-	
AT-1945	$_3M$	10,5	9,6	-	
AT-1959	M_3	10,8	10,2	-	
AT-2760	$_3M$	10	8,4	-	
AT-599	M_3	10,7	8,7	-	
AT-3182	M_3	11,4	9,6	-	
AT-942	M_3	11,9	9,5	-	
AT-2273	$_3M$	10,9	9,7	-	
Atapuerca-SH	M_3 (M)	(N38) 11,28±0,81	(N38) 9,76±0,72	-	[133, 70]
**Neanderthal**					
**Italy**					
Fate F3	M_3	12,7	11	-	[145]
**Croatia**					
Krapina 9	$_3M$	11,7	10,7	-	[146, 147]
Krapina 78	$_3M$	11,2	10,4	-	
Krapina F/H	$_3M$	12	10,7	-	
Krapina F/H	M_3	12,2	10,9	-	
Krapina MNDJ	M_3	11,8	11,3	-	
Krapina MNDK	$_3M$	12,1	10,6	-	
Krapina R63	M_3	11,7	-	-	
	(M) M3	(N11) 12,1±0,7	(N10) 10,8±0,4	-	
Krapina (J-H.G)					[70]
**France**					
Combe-Grenal XII	$_3M$	11,7	11,9	-	[178]
Grotte du Bison (Arcy sur Cure) P7-647-76	$_3M$	11,1	11,6	-	[149]
	M_3	11,1	9,8	-	
La Chaise de Vouthon					[148]
Abri Bourgeois Delaunay BD1	$_3M$	10,9	9,6	-	
La Ferrassie 10 LRM3 / LF10	M_3	11,3	10,3	-	[151]
La Ferrassie 12 LRP3 / LF 12	M_3	7,6	8,4	-	
La Quina 5	$_3M$	11,47	11,56	-	[152]
	M_3	11,47	11,56	-	

Inferior Maxillary (Measurements in mm)

**Sample/Specimens (*Genus*)**	**Tooth**	**MD (M81)**	**BL [M81 (1)]**	**Maximum root height**	***References***
La Quina 4b	$_3M$	11,3	12,23	-	
La Quina 9	$_3M$	12,6	12,4	-	
Monsempron					
Maxillaire h	M_3	12,6	11	-	[155]
Regourdou 1	$_3M$	11,64	10,87	-	[179]
	M_3	11,84	10,74	-	
**Greece**					
Lakonis I	$_3M$	11,55	10,9	-	[180]
**Hungary**					
Subalyuk 1	$_3M$	12,5	11,5	-	[181]
	M_3	11,4	11,5	-	[182]
	$_3M$	12,4	11,8	-	
**Iraq**					
Shanidar 1	M_3	11,6	10,9	-	[58]
	$_3M$	11,5	10,8	-	
Shanidar 2	M_3	11,2	10,8	-	
	$_3M$	11,7	11,2	-	
Shanidar 6	M_3	12,8	12,2	-	
**Israël**					
Amud 1	M_3	11,6	10,5	-	[162]
	$_3M$	11,8	10,8	-	
Kebara 2	$_3M$	11,5	11,3	-	[183]
	M_3	11,5	11,7	-	
Méarat Shovakh	$_3M$	11,6	10,9	-	[184]
Shovakh 1					
**Spain**					
Palomas 1	M_3	-11,7	10,6	-	[166, 167]
	$_3M$	-12	-	-	
**United Kingdom**					
Pontnewydd					
PN16	M_3	12,8	10,6	-	[168]
PN21	M_3	12,8	10,9	-	
Neanderthal	M3 (M)	(N 25) 11,4±0,9	(N 26) 10,9±1,1	-	[70]

Inferior Maxillary (Measurements in mm)

**Sample/Specimens (*Genus*)**	**Tooth**	**MD (M81)**	**BL [M81 (1)]**	**Maximum root height**	***References***
Homo sapiens					
**Israël**					
Qafzeh 9	M_3	12,9	11,9	-	[171]
Qafzeh 7	M_3	11,9	11,3	-	
Qafzeh 8	M_3	12,9	10,9	-	
Qafzeh 9	M_3	12,9	11,9	-	
	${}_3M$	12,6	11,9	-	
**Morocco**					
Djebel Irhoud 11	M_3	12,8	11,1	-	[173]

Data from [80]

**Ref.* [137]

Table S11. Circeo mandibular M3 dental sample. 3D enamel thickness, full crown, variables assessed in mandibular third molars from Guattari Cave and compared with samples/populations belonging to *H. erectus*, East and North African *Homo,* Middle Pleistocene European *Homo,* Neanderthals and fossil and extant *H. sapiens*. Variables: **Ve**, enamel volume; **Vcdp,** Dentin and coronal pulp volume; **Vpc,** coronal pulp chamber volume; **Vc,** coronal volume; **SEDJ**, cement enamel joint surface; **3D AET,** Average thickness of enamel (Ve/SEDJ); **3D RET** [100*3D AET/($Vcdp^{1/3}$)]; **L**, lateral. Circeo 11 (**C11**) and Circeo 12 (**C12**) present study; Circeo2 (**C2**) and Circeo3 (**C3**) mandibles, original *data*. Mean, Standard Deviation (**SD**) and range are given for the comparative sample when more than two samples are present.

**Taxon/ Sample**	**N**	**Tooth class**	**Ve mm³**	**Vcdp mm³**	**Vpc mm³**	**Vc mm³**	**Vcdp/Vc %**	**SEDJ mm²**	**3D AET (mm²)**	**3D RET**
**C11[1]**	**1**	M_3	**195,36**	**335,08**	**7,90**	**530,44**	**0,63**	**228,68**	**0,85**	**12,32**
**C12[1]**	1	${}_3M$	**168,38**	**308,36**	**7,12**	**476,74 Vdc 301,24**	**0,65 1,02**	**199,32**	**0,84**	**12,53**
**C2[1]**	1	M_3	**185,89**	**130,53**	**2,20**	**316,42**	**0,41**	**139,95**	**1,33**	**26,23**
**C3[1]**	1	M_3	**148,64**	**130,57**	**2,24**	**279,21**	**0,47**	**128,41**	**1,16**	**22,85**
***A. africanus* [2]**										
STW 412b	1	${}_3M$	345,47	-	-	-	-	-	-	12,07
STW 529	1	${}_3M$	554,49	-	-	-	-	-	-	19,34
STW 560b	1	${}_3M$	551,06	-	-	-	-	-	-	15,20
***H. antecesssor*[3]**										
TD6 (level)										
AT6-5	1	M_3	229,21	261,93	-	-	53,33	191,97	1,19	18,66
ATD6-96	2	M_3	109,39	55,52	-	-	33,67	81,15	1,35	35,33
AT6-113	1	M_3	210,24	185,64	-	-	46,89	158,77	1,32	23,21
Mean	4		182,95	167,70	-	350,65	44,63	143,96	1,29	25,74
SD			64,41	104,37	-	167,76	10,02	56,88	0,08	8,62
Range			109,39-229,21	55,52-261,93	-	164,91-491,15	33,67–53,33	81,15-191,97	1,19-1,35	18,66–35,33
***H. erectus*[3]**										
Sangiran (NG9107.2)	1	M_3	174,8	195,50	-	370,30	52,80	167,2	1.05	18,01
**MPEH[3]**										
M-LN	1	M_3	264,01	246,84	-	510,85	48,32	181,19	1,46	23,23

Taxon/ Sample	N	Tooth class	Ve mm³	Vcdp mm³	Vpc mm³	Vc mm³	Vcdp/Vc %	SEDJ mm²	3D AET (mm²)	3D RET
**SH**										
AT30	1	M_3	196,72	181,82	-	378,54	-	148,03	-	-
AT-811	1	M_3	169,39	229,18	-	398,57	-	161,69	-	-
AT-143	1	M_3	221,17	191,13	-	412,30	-	148,9	-	-
AT-1468	1	M_3	206,78	185,12	-	391,90	-	143,8	-	-
AT-599	1	M_3	192,31	159,05	-	351,36	-	128,61	-	-
AT-942	1	M_3	243,94	223,23	-	467,17	-	168,73	-	-
AT-1959	1	M_3	195,74	195,82	-	391,56	-	147,7	-	-
AT-2438b	1	M_3	194,04	157,57	-	351,61	-	128,78	-	-
AT-2273	1	M_3	213,32	175,75	-	389,07	-	143	-	-
AT-2777	1	M_3	237,19	212,61	-	449,80	-	155,45	-	-
AT-3182	1	M_3	221,38	203,19	-	424,57	-	149,63	-	-
AT-3943	1	M_3	175,64	162,49	-	338,13	-	137,37	-	-
**SH**										
Mean	12	M_3	205,64	189,75	-	395,38	47,95	146,81	1,40	24,54
SD			22,79	24,16	-	39,02	3,35	11,91	0,14	2,85
Range			169,39-<br>243,94	157,57-<br>229,18	-	338,13-<br>467,17	44,81–<br>57,50	128,61-<br>168,73	1,05-1,53	17,12-<br>27,90
**NAH[3]**										
Tighenif 2	1	M_3	372,24	371,53	-	743,77	49,95	255,48	1,46	20,27
**NEA[3]**										
Abri Bourgeois-Delaunay[3,4] BD1	2	M_3	146,84(l)<br>58,25(r)	222,97<br>221,19	-	-	60,29<br>58,29	169,27<br>174,79	0,87<br>0,91	14,31<br>14,97
Abri-Suard S36	1	M_3	163,18	244,62	-	407,80	59,99	177,94	0,92	14,66
Abri-Suard S43	1	M_3	185,4	285,54	-	470,58	60,68	142,85	1,30	19,67
Krapina D9	1	M_3	204,74	343,83	-	548,57	62,68	228,03	0,90	12,82

Taxon/ Sample	N	Tooth class	Ve mm^3	Vcdp mm^3	Vpc mm^3	Vc mm^3	Vcdp/Vc %	SEDJ mm^2	3D AET (mm^2)	3D RET
Krapina D57	1	M_3	246,77	259,29	-	506,06	51,24	192,94	1,28	20,06
Krapina D85	1	M_3	262,18	253,05	-	515,23	49,11	186,03	1,41	22,28
La-Quina Q760-H9	1	M_3	222,11	485,91	-	708,01	68,63	192,52	1,15	14,67
Combe Grenal CG XII	1	M_3	239,40	300,14	-	539,54	55,63	206,76	1,16	17,29
Mean	9		208,03	301,62	-	509,65	58,88	188,37	1,11	16,75
SD			37,51	75,22	-	88,61	6,14	22,8	0,20	3,37
Range			154,04-262,18	244,62-485,90	-	407,80-708,01	49,11-68,82	142,85-228,03	0,82-1,40	12,73-22,28
Regourdou1	1	M_3	171,16	356,03	-	-	-	-	-	13,14
**NEA**[3]										
Mean	11	M_3	211,65	293,39		555,04	57,77	187,16	1,14	17,31
SD			34,66	70,87		81,51	6,08	21,09	0,19	3,30
Range			154,04–262,18	228,03–485,90		407,80–708,01	49,11–68,63	142,85–228,03	0,82–1.41	12,74–22,28
**MH**[3]										
B998-scht3	1		245,64	228,53	-	474,17	48,20	192,40	1,28	20,88
B998-scht1	1		343,86	328,87	-	672,73	48,89	243,27	1,41	20,48
San Canziano	1		226,14	190,69	-	416,83	45,75	185,30	1,22	21,20
MH-CZ	1		243,12	237,26	-	480,37	49,39	191,65	1,27	20,49
MH-UdP	1		206,51	170,48	-	376,99	45,22	178,26	1,16	20,89
B996-scht1	1		362,14	405,48	-	767,63	52,82	273,48	1,32	17,89
B996-scht2	1		254,55	288,75	-	543,30	53,15	216,63	1,18	17,78
B996-scht2-mand2	1		166,58	167,74	-	334,32	50,17	153,79	1,08	19,64
?			178,53	149,70	-	328,23	45,61	121,09	1,47	27,77
?			181,56	146,81	-	328,37	44,71	125,05	1,45	27,52
?			196,10	242,06	-	438,16	55,24	149,94	1,31	20,99

Taxon/ Sample	N	Tooth class	Ve mm^3	Vcdp mm^3	Vpc mm^3	Vc mm^3	Vcdp/Vc %	SEDJ mm^2	3D AET (mm^2)	3D RET
?			263,03	250,84	-	513,87	48,81	182,51	1,44	22,85
?			299,26	372,37	-	671,63	55,44	162,05	1,85	25,67
Mean	8		243,62	244,58	-	488,20	49,49	182,72	1,34	21,85
SD			61,68	83,91	-	142,74	3,72	43,51	0,19	3,25
Range			166,58-362,14	146,81-405,48	-	328,23-767,63	44,70-55,44	121,09-273,48	1,08-1,85	17,78-27,77
Mean	20	M_3	232,09	223,86	-	455,95	48,53	171,91	1,36	22,82
SD			53,13	74,10	-	124,08	3,87	38,84	0,18	3,53
Range			166,58–362,14	145,16–405,48	-	328,23–767,08	42.18–55.44	121,09–273,48	1.08–1.85	17.78–30,20

Taxon/ Sample	N	Tooth class	LVe	LVcdp	LVpc	LVc	LVcdp/LVc	LSEDJ	3D LAET (mm)	3D LRET
**C11**[1]		M_3	**78,80**	**295,55**	**-**	**374,35**	**0,79**	**110**	**0,72**	**10,77**
**C12**[1]		$_3M$	**66,47**	**269,11**	**7,12**	**335,58**	**0,80**	**121,25**	**0,55**	**8,51**
**C2**[1]		M_3	**56,18**	**102,04**	**-**	**158,22**	**0,64**	**68,56**	**0,82**	**17,56**
**C3**[1]		M_3	**49,78**	**113,62**	**-**	**163,40**	**0,70**	**66,92**	**0,74**	**15,38**
H. antecessor[3]										
**TD6** (level)										
AT6-5	1		39,16	212,36	-	251,52	84,43	80,55	0,49	8,15
ATD6-96	1		6,91	26,48	-	33,39	79,31	79,31	0,40	13,47
AT6-113	1		25,34	127,31	-	152,65	83,40	83,40	0,49	9,83
Mean	3	M_3	23,80	122,05	-	144,85	82,38	49,67	0,46	10,48
SD			16,18	93,05	-	109,22	2,71	31,70	0,05	2,72
Range			6,91–39,16	93,05 – 122,05	-	33,39 – 251,52	79.31–84.43	17,21 – 80,55	0,40-0,49	8,15–13,47
H. erectus[3]										
Sangiran (NG9107.2)	1	M_3	29,41	145,13	-	174,54	83,15	64,21	0,46	8,72

Taxon/ Sample	N	Tooth class	LVe	LVcdp	LVpc	LVc	LVcdp/LVc	LSEDJ	3D LAET (mm)	3D LRET

Taxon/ Sample	N	Tooth class	LVe	LVcdp	LVpc	LVc	LVcdp/LVc	LSEDJ	3D LAET (mm)	3D LRET
**MPEH[3]**										
M-LN	1	M_3	68,05	207,24	-	275,29	75,28	88,75	0,77	12,96
**SH**										
AT30	1	M_3	35,34	136,76	-	172,10	79,47	62,92	0,56	10,90
AT-811	1	M_3	53,27	194,36	-	247,63	78,49	90,32	0,59	10,18
AT-143	1	M_3	46,77	159	-	205,77	77,27	72,33	0,65	11,94
AT-1468	1	M_3	50,54	157,42	-	207,96	75,70	75,29	0,67	12,43
AT-599	1	M_3	50,84	136,58	-	187,42	72,87	71,66	0,71	13,78
AT-942	1	M_3	64	190,62	-	254,62	74,86	89,77	0,71	12,39
AT-1959	1	M_3	42,45	156,58	-	199,03	78,67	50,21	0,85	15,69
AT-2438b	1	M_3	37,44	122,91	-	160,35	76,65	61,79	0,61	12,19
AT-2273	1	M_3	38,98	136,47	-	175,45	77,78	64,02	0,61	11,83
AT-2777	1	M_3	64	179,87	-	243,87	73,76	62,48	0,78	13,75
AT-3182	1	M_3	56,30	170,83	-	227,13	75,21	76,29	0,74	13,30
AT-3943	1	M_3	46,81	135,04	-	181,85	74,26	70,35	0,67	12,97
**SH**										
Mean	12	M_3	48,90	156,37	-	205,27	76,25	72,79	0,68	12,61
SD			9,54	23,61	-	31,79	2,12	11,75	0,08	1,44
Range			35,34–64	122,91–194,36	-	160,35–254,62	72,87–79,47	50,21–90,32	0,56-0,85	10,18-15,69
**NAH[3]**										
Tighenif 2	1	M_3	79,89	302,65	-	382,54	79,12	124,33	0,64	9,57
**NEA[3]**										
Krapina D5	1	M_3	67,70	276,21	-	343,92	80,31	123,28	0,55	8,43
Krapina D7	1	M_3	83,44	232,45	-	315,89	73,59	116,58	0,72	11,64

Krapina D85	1	M_3	66,24	195,19	-	261,43	74,66	98,02	0,68	11,65
Krapina D106	1	M_3	80,33	314,03	-	394,36	79,63	132,28	0,61	8,93
Regourdou	1	M_3	51,51	219.06	-	270,57	80,96	98,97	0,52	8,63
Regourdou	1	M_3	48,02	211,39	-	259,42	81,49	97,61	0,49	8,26
**NEA[3]**										
Mean	6	M_3	66,21	241,39	-	307,60	78,44	111,12	0,59	9,59
SD			14,46	44,95	-	54,29	3,42	15,01	0,09	1,61
Range			48,02–83,44	195,19–314,03	-	259,42–394,36	73,59–81,49	97,61–138,28	0,49–0,72	8,26–11,65
**MH[3]**										
MH-UdP	1	M_3	38,24	135,23	-	173,46	77,96	81,94	0,47	9,09
MH-CZ	1	M_3	49,39	228,74	-	278,13	82,24	99,06	0,50	8,15
B996 scht1	1	M_3	79,90	314,86	-	394,75	79,76	131,75	0,61	8,91
B998 scht2	1	M_3	51	191,97	-	242,98	85,40	79,01	0,60	10,35
B998 scht2 mand.2	1	M_3	22,25	130,04	-	152,29	85,39	69,99	0,32	6,28
B996 scht1	1	M_3	75,47	262,70	-	338,16	77,68	116,24	0,65	10,14
Mean	6		52,71	210,59	-	263,30	80,34	97,40	0,52	8,82
SD			21,95	72,71	-	93,71	2,96	23,12	0,12	1,49
Range			22,25-79,90	130,04-314,86	-	152,29-394,75	77,68-85,39	69,99-131,75	0,32-0,65	6,28-10,35
Mean	13	M_3	47,92	176,58	-	224,50	78,62	84,04	0,57	10,24
SD			16,65	60,43	-	75,60	3,15	21,75	0,10	2,01
Range			22,25–79,90	114,33–314,86	-	152,29-394,75	73,75–85,39	56,12–131,75	0,32–0,71	6,28–13,98

TD6, Atapuerca-Gran Dolina; **NAH**, North African *Homo*; **SH**, Sima de los Huesos; **MPEH,** Middle Pleistocene European *Homo*; **M-LN**, Montmaurin-La Niche; **NEA**, Neanderthal; **MH**, Modern humans (fossil and extant *H. sapiens*). Comparison data was taken from:

Refs.

[1] **Original *data***

[2] [30]

[3] [134, 33]

[4] [135]

Table S12. Circeo mandibular M3 dental sample 3D taurodontism indices. Vcervix, volume of cement and pulp up to the bifurcation; **Vbranch,** volume of cement roots and root canals; **VBI %,** volumetric bifurcation index [Vcervix/(Vcervix+Vbranch)*100]. Circeo 11 (**C11**) and Circeo 12 (**C12**) present study; Circeo2 (**C2**) and Circeo3 (**C3**) mandibles, **original *data***.

Specimens	Tooth	Vcervix	Vbranch	VBI %	Classification	Taurodontism Index [32]
**C11**	$\mathbf{M_3}$	**446,30**	**204,41**	**68,59**	**Mesotaurodont**	0-24,9 No Taurodontism
**C12**	$\mathbf{_3M}$	**464,86**	**312,90**	**59,77**	**Mesotaurodont**	25,0-49,9 Hypotaurodontism
**C2**	$\mathbf{M_3}$	**505,53**	**520**	**49,29**	**Hypotaurodont**	50,0-74,9 Mesotaurodontism
**C3**	$\mathbf{M_3}$	**244,31**	**497,44**	**32,96**	**Hypotaurodont**	75,0-100 Hypertaurodontism

Table S13. Circeo 6 (C6) mandible. Ante and post-mortem tooth loss.

**Inferior Maxillary Circeo 6 (C6)**		
teeth	right	left
I1	-* normal bone level	− normal bone level
I2	−	− *
C	−	+**
P3	+ Alveolus oriented distally	traces of alveolus
P4	− ***	traces of alveolus
M1	− ***	ND
M2	ND	ND
M3	ND	ND

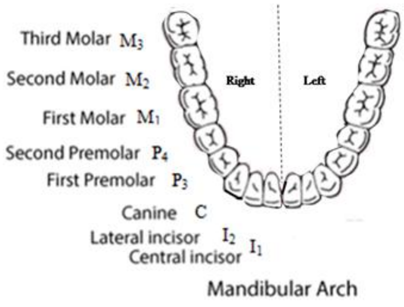

-* Lost *intra-vitam*. Incomplete alveolar closure
− Lost *intra-vitam*. Complete closure
\+ Lost *post-mortem*. incomplete *alveolus*
ND = Not Detectable
** The verticality, depth, and buccal dilatation of the alveolar bone are compatible with a canine
*** presents bone atrophy and height reduction on the buccal wall of the alveolar bone

Table S14. Circeo 7 (C7) femur diaphysis - Linear diaphyseal diameters (measurements mm). **MPMH**, Middle Paleolithic modern human; **EUP/MUP**, Early and Mid-Upper Paleolithic; **M–L**, Medio-lateral and **A–P**, Antero-posterior diameter; **(r)**, right; **(l)**, left; **(N)**, number.

	**Proximal**		**Midshaft**		**Distal**	
**Sample/Specimens**	**A-P (M10)**	**M-L (M9)**	**A-P (M6)**	**M-L (M7)**	**A-P**	**M-L**
**C7** (present study)	**27,2**	**36,5**	**27,8**	**35,1**	**28.5**	**32.4**

**Early Pleistocene** (EP)						
Dmanisi D4167 (r)	-	-	26,5	22,2	-	-
KNM-ER 736 (l)	30	40	38,8	36,2	-	-
KNM-ER 737 (l)	27,1	39,3	27,4	32,6	-	-
KNM-ER 803a, b (l)	(27,6)	(34,4)	28,2	32,1	-	-
KNM-ER 1472 (r)	22	32	(26,1)	(26,5)	-	-
KNM-ER 1481a (l)	23	30,8	22,5	25,3	-	-
**Middle Pleistocene** (MP)						
Aïn Maarouf 1 (l)	-	-	26,2	26,7	-	-
Arago 48 (r)	27,1	35,2	-	-	-	-
Arago (Tautavel)* (l)	-	-	-	-	32,55	36,9
Berg Aukas 1 (l)	30	40,8	35,9	31,5	-	-
Broken Hill E689 (l)	27	30,4	-	-	-	-
Broken Hill E690 (l)	21,8	31,1	28,6	24,6	-	-
Broken Hill E793 (l)	-	-	29,7	26	-	-
Castel di Guido 1 (r)	-	-	35	32	-	-
La Chaise-BD 5 (r)	23	29	26	27	-	-
Ehringsdorf 5 (r)	26,8	37,1	30,2	33	-	-
Gesher-B-Y 1 (r)	19,6	28,2	21,5	24	-	-
Gesher-B-Y 2 (r)	-	-	27	28	-	-
Kresna 11 (l)	25	32,5	26,5	28,58	-	-
Mammolo 1 (r)	29	35,2	32	28,8	-	-
OH 28 (l)	23,3	37,4	24,7	32,7	-	-
Sedia del Diavolo (r)	-	-	31	29	-	-
Tabun E1 (r)	-	-	(26)	(29)	-	-
Zhoukoudian 1 (l)	23,2	34,3	27,1	29,7	-	-
Zhoukoudian 2 (l)	-	-	22,8	26,4	-	-
Zhoukoudian 4 (r)	22,7	34,3	25	29,3	-	-
Zhoukoudian 5 (l)	-	-	23,7	29,5	-	-
Zhoukoudian 6 (l)	-	-	26,1	29,2	-	-
Central Narmada Valley (India) NTK-F-07-05*	-	-	-	-	38.05	44,85
H. erectus (Java)*	-	-	-	-	34,15	37,65
**Neanderthal**						
Amud 1 (r)	30,3	36,5	-	-	-	-
Amud 1 (l)	29,4	36,9	32,3	32,4	-	-
Chapelle-aux-Saints (r)	29,4	33	-	-	-	-
Chapelle-aux-Saints (l)	-	-	31,1	29,1	-	-
Feldhofer 1 (r)	29	32,8	31,3	28,2	-	-
Feldhofer 1 (l)	29,3	35,5	30,5	30,6	-	-
Ferrassie 1 (r)	29,9	38	29,8	32	-	-
Ferrassie 1 (l)	29,7	36	29,1	31	-	-
Ferrassie 2 (r)	27,6	33,4	26,4	29,6	-	-
Ferrassie 2 (l)	27,9	32	28,1	28,6	-	-
Fond-de-Forêt 1 (l)	-	-	33	30,3	-	-
Hortus 34 (l)	24	30	-	-	-	-
Krapina 213 (l)	26,1	36,4	-	-	-	-
Krapina 214 (l)	22,3	29,6	-	-	-	-
Krapina 257.32 (l)	24,6	33,6	-	-	-	-
Krapina 257.33 (l)	21,9	33	-	-	-	-
Palomas 52 (l)	19,2	25,5	-	-	-	-
Palomas 96 (r)	-	-	24,4	24,2	-	-
Quina 5 (l)	26	33,5	26	30	-	-
Quina 38 (l)	26,8	34,5	-	-	-	-
Rochers-de-V.1 (l)	-	-	29,9	27,9	-	-
Saint-Césaire 1 (r)	29,1	35,9	31,3	32,5	-	-
Santa-Croce 1 (r)	25,5	30,8	29,5	28,8	-	-
Shanidar 1 (r)	28,6	35,3	-	-	-	-
Shanidar 4 (r)	-	-	35,5	31,1	-	-
Shanidar 4 (l)	31	36,5	-	-	-	-
Shanidar 5 (r)	-	-	36,5	33,2	-	-
Shanidar 6 (r)	-	-	28,5	28	-	-
Shanidar 6 (l)	24,6	30,5	-	-	-	-
Spy 2 (r)	28,4	35	29,3	29,1	-	-
Spy 2 (l)	28,2	35,8	28,5	29,4	-	-
Stadelhöhle 1 (r)	26,6	31	29	29,6	-	-
Tabun 1 (r)	22,6	30,4	24	27,4	-	-
Tabun 3 (r)	-	-	22,5	24,1	-	-
Zafarraya 1 (r)	28	34	-	-	-	-
Neanderthal (r)*	-	-	-	-	40.7	47,3
**MPMH**						
Qafzeh 3 (l)	-	-	32,8	26	-	-
Qafzeh 8 (r)	29	39,8	41,3	30,2	-	-
Qafzeh 9 (r)	27,3	32,4	37,8	29,1	-	-
Qafzeh 9 (l)	27,1	32,5	-	-	-	-
Skhul 3 (r)	-	-	35,7	30,7	-	-
Skhul 4 (r)	26,5	32	33,5	26,9	-	-

Skhul 4 (l)	26	33,3	33,6	25,2	-	-
Skhul 5 (r)	32,5	28,8	39,6	27,6	-	-
Skhul 5 (l)	29,4	30,9	37,9	26,9	-	-
Skhul 6 (l)	26,2	30,4	34,4	27	-	-
Skhul 7 (r)	-	-	26,8	27,6	-	-
Skhul 9 (l)	29,3	38,3	-	-	-	-
Skhul '7' (l)	-	-	32	27	-	-
Skhul '9' (l)	-	-	23,5	25	-	-
H. sapiens (N 18) (Modern Eastern Indians)*	-	-	-	-	27.68	34,87

	Proximal		Midshaft		Distal	
**Sample/Specimens**	**A-P (M10)**	**M-L (M9)**	**A-P (M6)**	**M-L (M7)**	**A-P**	**M-L**
**EUP/MUP**						
Arene Candide 1 (r)	26	32	31	24	-	-
Arene Candide 1 (l)	26	31	32	26	-	-
Barma Grande 1 (r)	29,5	42,5	-	-	-	-
Barma Grande 1 (l)	31	41,5	-	-	-	-
Barma Grande 2 (r)	31	40	-	-	-	-
Barma Grande 2 (l)	31	40,5	-	-	-	-
Barma Grande 6 (r)	31,5	42,5	41	31	-	-
Caviglione 1 (r)	28,8	37,2	36,6	28,4	-	-
Cro-Magnon 1 (r)	30,6	38,5	40,1	31,3	-	-
Cro-Magnon 1 (l)	28,7	39	37,4	30	-	-
Cro-Magnon 4323A (r)	25,7	36,3	-	-	-	-
Cro-Magnon 4322 (l)	25,9	36	32	26,9	-	-
Cro-Magnon 4324 (l)	-	-	32,2	25,6	-	-
Dolní Věstonice 3 (r)	20,8	30,6	26,2	23,3	-	-
Dolní Věstonice 3 (l)	19,8	29,8	25,5	22,5	-	-
Dolní Věstonice 13 (r)	25,6	34,1	30,5	26,4	-	-
Dolní Věstonice 13 (l)	23,5	35,2	29,5	27,1	-	-
Dolní Věstonice 14 (r)	24,3	38,3	31,1	26,2	-	-
Dolní Věstonice 14 (l)	25,3	37,6	32,3	24,7	-	-
Dolní Věstonice 16 (r)	27,8	34,6	34,8	26,9	-	-
Dolní Věstonice 16 (l)	28,1	34,8	36	26,6	-	-
Dolní Věstonice 35 (r)	24,3	35	34,9	29,1	-	-
Grotte des Enfants 4 (r)	29,5	38	37	30	-	-
Grotte des Enfants 4 (l)	30	38	37	30	-	-
Grotte des Enfants 5 (-)	27	33	-	-	-	-
Minatogawa 1 (r)	23	29	26,5	26	-	-
Minatigawa 1 (l)	22	30	26,5	26,5	-	-
Minatogawa 2 (l)	18	25	21	22	-	-
Minatogawa 3 (r)	19	26,5	24	23,5	-	-
Minatogawa 3 (l)	19	26	24	23,5	-	-
Minatogawa 4 (r)	18,5	26	21	23	-	-
Mladeč 27 (r)	25,5	30	28,3	24	-	-
Mladeč 28 (l)	23,5	33,8	-	-	-	-
Nahal´En-Gev 1 (l)	22	28	26,5	23,2	-	-
Ohalo 2 (r)	27,5	29,8	37,7	26,4	-	-
Ohalo 2 (l)	26,7	30,8	35,4	27	-	-
Paglicci 25 (r)	26	35	34	27	-	-
Paglicci 25 (l)	26	33,5	33,5	27,5	-	-
Paviland 1 (l)	26,8	35,9	32,3	27,4	-	-
Pavlov 1 (r)	27,4	36,5	32,6	28,4	-	-
Pavlov 1 (l)	26,9	37,3	31,2	29,8	-	-
Předmostí 3 (r)	24	38	30,8	30	-	-
Předmostí 3 (l)	24	38	31	29	-	-
Předmostí 4 (r)	25	35,8	29	28	-	-
Předmostí 4 (l)	24	38	31	29	-	-
Předmostí 9 (r)	23	33	27	25	-	-
Předmostí 9 (l)	23	33	27,5	26	-	-
Předmostí 10 (r)	22,6	35	25,4	27,5	-	-
Předmostí 10 (l)	23	35,3	24,5	27,3	-	-
Předmostí 14 (r)	22,5	33	26,4	26,4	-	-
Předmostí 14 (l)	23,4	35	26	27,5	-	-
Rochette 2 (r)	26,5	33	29	26	-	-
Sunghir 1 (r)	27,1	39,4	34,6	33,8	-	-
Sunghir 1 (l)	28	38	35	30,8	-	-
Sunghir 4 (l)	27,4	33,7	33,5	26,8	-	-
Tianyuan 1 (r)	26,2	33,6	-	-	-	-
Tianyuan 1 (l)	26,5	31,5	-	-	-	-
Veneri 1 (r)	29	41	39	31	-	-
Veneri 1 (l)	31	37	37	31	-	-
Veneri 2 (r)	30	34	35	29	-	-

Veneri 2 (l)	30	37	35	29	-	-
Willendorf 1 (r)	-	-	28,3	24	-	-
Zhoukoudian UC 67 (r)	26,5	32,7	36	25,9	-	-
Zhoukoudian UC 68 (r)	24,7	29	32,3	23,4	-	-

Data from [80]
Ref. [137]

Table S15. Cross-sectional geometric parameters of the Circeo 7 (C7) femur and comparison. Distal (**20%**) femoral diaphysial cross-sectional parameters. **Ta**, Total area (mm$^{2)}$; **Imax**ortical area (mm^2); **Ix** (mm^4), anterior-posterior bending rigidity; **Iy,** (mm^4) medio-lateral bending rigidity; **Imax,** (mm^4), maximum bending rigidity; **Imin**, (mm^4), minimum bending rigidity; **J**, polar second moment of area; **%CA**, Relative cortical thickness (percent cortical area= %CA = (CA/TA) × 100); **Imax/Imin**, diaphyseal circular index; **MPMH**, Middle Paleolithic modern human; **EUP/MUP**, Early and Mid-Upper Paleolithic; **(r)**, right; **(l)**, left.

Sample/Specimens	TA	CA	Ix	Iy	Imax	Imin	J	%CA	Imax/Imin
**Circeo7** (Original *data*)	**840**	**547**	**48851**	**50361**	**61789**	**37424**	**99213**	**65,10**	**1,65**
**Early Pleistocene**									
KNM-ER 737 (l)	808	370	28961	48254	48333	27882	76215	45,79	1,73
KNM-ER 1472 (r)	529	308	15417	22226	22319	15324	37643	58,22	1,46
KNM-ER 1481a (l)	509	260	13566	18136	18196	13505	31702	51,08	1,35
KNM-ER 1808mn1 (r)	711	520	32276	43323	43465	32134	75599	73,14	1,35
**Middle Pleistocene**									
Aïn Maarouf 1 (l)	662	311	22542	28260	28892	21909	50802	46,98	1,32
**Neanderthal**									
Amud 1 (l)	1000	517	58991	62572	63098	58465	121563	51,70	1,08
Chapelle-aux-Saints 1 (l)	8367	418	36092	47688	48723	35057	83780		
Feldhofer 1 (r)	919	274	31411	36684	36686	31409	68095	29,82	1,17
Ferrassie 1 (l)	937	443	46152	53432	53434	46149	99584	47,28	1,16
Fond-de-Forêt 1 (l)	930	275	30076	39441	40492	29024	69517	29,57	1,40
Spy 2 (r)	947	305	31861	45360	45389	31832	77221	32,21	1,43
**MPMH**									
Skhul 4 (r)	943	426	44747	54166	54794	44120	98914	45,17	1,24
Skhul 4 (l)	904	392	41440	48006	49968	39478	89446	43,36	1,27
Skhul 6 (l)	853	386	35323	46685	47709	34299	82008	45,25	1,39
**EUP/MUP**									
Cro-Magnon 1 (r)	1119	488	61219	81509	82822	59907	142728	43,61	1,38
Cro-Magnon 4322 (l)	841	358	30124	49905	50759	29269	80029	42,57	1,73
Dolní Věstonice 3 (r)	673	251	17702	26902	26917	17687	44604	37,30	1,52
Dolní Věstonice 13 (r)	847	321	31994	39404	39554	31844	71398	37,90	1,24
Dolní Věstonice 13 (l)	864	338	31167	46048	46063	31152	77215	39,12	1,48
Dolní Věstonice 14 (l)	904	292	30552	42570	43619	29503	73122	32,30	1,48
Dolní Věstonice 16 (r)	786	301	30552	31830	32157	30226	62382	38,30	1,06
Dolní Věstonice 16 (l)	856	277	28614	33754	33820	28548	62368	32,36	1,18
Minatogawa 12 (r)	840	342	30080	43180	43190	30070	73260	40,71	1,44
Minatogawa 12 (l)	866	297	28450	40970	41410	28010	69420	34,30	1,48
Minatogawa 3 (r)	559	300	17280	21670	21690	17260	39950	53,67	1,26
Minatogawa 3 (l)	569	253	14800	21210	21240	14770	36010	44,46	1,44
Minatogawa 4 (r)	607	203	12220	22180	22320	12080	34400	33,44	1,85
Mladeč 27 (r)	880	311	32414	40825	41359	31880	73239	35,34	1,30
Ohalo 2 (r)	945	362	44528	45095	48908	40716	89624	38,31	1,20

Ohalo 2 (l)	892	367	41285	43090	45856	38519	84375	41,14	1,19
Paviland 1 (l)	938	296	32225	43586	43593	32218	75812	31,56	1,35
Pavlov 1 (r)	980	269	26228	49395	49397	26225	75622	27,45	1,88
Pavlov 1 (l)	934	293	29448	47559	47609	29398	77007	31,37	1,62
Tianyuan 1 (r)	906	495	67464	41012	67464	41012	108476	54,64	1,64

Data from [80].

Table S16. Cross-sectional geometric parameters of the Circeo 7 (C7) femur and comparison. Mid-Distal (**35%**) femoral diaphysial cross-sectional parameters. **Ta**, Total area ($mm^{2)}$; **CA**, Cortical area (mm^2); **Ix** (mm^4), anterior-posterior bending rigidity; **Iy,** (mm^4) medio-lateral bending rigidity; **Imax,** (mm^4), maximum bending rigidity; **Imin**, (mm^4), minimum bending rigidity; **J**, polar second moment of area; **%CA**, Relative cortical thickness (percent cortical area= %CA = (CA/TA) × 100); **Imax/Imin**, diaphyseal circular index; **MPMH**, Middle Paleolithic modern human; **EUP/MUP**, Early and Mid-Upper Paleolithic; **(r)**, right; **(l)**, left.

Sample/Specimens	TA	CA	Ix	Iy	Imax	Imin	J	%CA	Imax/Imin
**Circeo7** (Original *data*)	**678**	**557**	**30902**	**40283**	**41542**	**29643**	**71184**	**82,16**	**1,40**
**Early Pleistocene**									
KNM-ER 736 (l)	963	658	67261	66338	70710	62889	133599	68,33	1,12
KNM-ER 737 (l)	662	445	28850	33691	33942	28599	62541	67,22	1,19
KNM-ER 803a (l)	636	505	31051	32282	38414	24919	63333	79,40	1,54
KNM-ER 1472 (r)	470	359	16093	17127	18438	14782	33220	76,38	1,25
KNM-ER 1481a (l)	382	310	10682	12069	13276	9475	22751	81,15	1,40
KNM-ER1808mn (r)	552	469	21873	26396	27162	21108	48269	84,96	1,29
**Middle Pleistocene**									
Arago 53 (r)	772	628	--	--	48121	34238	82359	81,35	1,41
Aïn Maarouf 1 (r)	498	396	19881	18232	20250	17864	38113	79,52	1,13
Broken Hill E690 (l)	499	414	22112	17148	22118	17142	39260	82,97	1,29
Gesher-B.-Y. 1 (r)	394	262	9622	12823	13071	9374	22445	66,50	1,39
Kresna 11 (l)	569	422	23912	25593	27388	22133	49505	74,17	1,24
OH 28 (l)	588	399	21530	28738	29509	20759	50268	67,86	1,42
**Neanderthal**									
Amud 1 (l)	775	599	51892	39696	53620	37968	91588	77,29	1,41
Chapelle-aux-Saints 1 (l)	657	445	33859	27877	34284	27453	61736	67,73	1,25
Feldhofer 1 (r)	697	459	38222	30716	39370	29568	68938	65,85	1,33
Ferrassie 1 (r)	776	471	42459	39197	46212	35444	81656	60,70	1,30
Ferrassie 1 (l)	760	531	39724	43353	46004	37074	83078	69,87	1,24
Ferrassie 2 (r)	622	424	26175	29345	30678	24842	55520	68,17	1,23
Ferrassie 2 (l)	677	416	32134	30356	33858	28632	62490	61,45	1,18
Fond-de-Forêt 1(l)	752	509	45374	37252	50467	32159	82626	67,69	1,57
Spy 2 (r)	668	451	32087	31168	32088	31167	63255	67,51	1,03
Tabun 1 (r)	535	381	20279	20636	21911	19003	40915	71,21	1,15
**MPMH**									
Qafzeh 9 (r)	730	625	48111	35572	48259	35425	83683	85,62	1,36
Skhul 4 (r)	685	447	37618	29555	37666	29507	67173	65,26	1,28
Skhul 4 (l)	629	451	38928	22957	38937	22948	61885	71,70	1,70
Skhul 5 (r)	804	518	64322	33788	65225	32884	98109	64,43	1,98
Skhul 6 (l)	684	449	36754	29667	37241	29180	66421	65,64	1,28
Skhul 7 (r)	502	363	19351	18481	22251	15581	37832	72,31	1,43

Sample/Specimens	TA	CA	Ix	Iy	Imax	Imin	J	%CA	Imax/Imin
**EUP/MUP**									
Cro-Magnon 1 (r)	878	483	61147	45019	61154	45012	106166	55,01	1,36
Cro-Magnon 1 (l)	774	492	57528	33746	58193	33081	91274	63,57	1,76
Cro-Magnon 4322 (l)	632	428	34204	25472	34693	24983	59676	67,72	1,39
Cro-Magnon 4328 (r)	611	461	33403	24138	33456	24085	57541	75,45	1,39
Dolní Věstonice 3 (r)	476	262	14763	14607	14936	14434	29370	55,04	1,03
Dolní Věstonice 3 (l)	466	264	14855	13504	14895	13464	28359	56,65	1,11
Dolní Věstonice 13 (r)	638	382	29950	25720	30996	24674	55670	59,87	1,26
Dolní Věstonice 13 (l)	668	405	30546	29943	32416	28074	60489	60,63	1,15
Dolní Věstonice 14 (r)	615	330	25721	22483	26686	21519	48204	53,66	1,24
Dolní Věstonice 14 (l)	618	339	28176	21324	28322	21179	49501	54,85	1,34
Dolní Věstonice 16 (r)	645	356	32425	22858	32766	22517	55283	55,19	1,46
Dolní Věstonice 16 (l)	667	334	31244	23461	31315	23389	54704	50,07	1,34
Dolní Věstonice 40 (r)	495	403	20801	17981	22440	16342	38782	81,41	1,37
Minatogawa 1 (r)	616	334	23670	24010	24060	23620	47680	54,22	1,02
Minatogawa (l)	563	316	19060	21850	22010	18910	40920	56,13	1,16
Minatogawa 3 (l)	420	308	13630	12680	13670	12640	26310	73,33	1,08
Minatogawa 3 (r)	429	373	14600	14470	14930	14140	29070	86,95	1,06
Minatogawa 4 (r)	414	245	9960	12990	13030	9920	22950	59,18	1,31
Mladeč 27 (r)	562	310	25065	15758	25077	15746	40823	55,16	1,59
Nahal 'En-Gev 1 (l)	465	355	15825	17003	17207	15621	32828	76,34	1,10
Ohalo 2 (r)	694	424	44392	24925	44472	24845	69317	61,10	1,79
Ohalo 2 (l)	703	477	42782	30665	42816	30630	73446	67,85	1,40
Paviland 1 (l)	678	379	35154	25022	35157	25019	60176	55,90	1,41
Pavlov 1 (r)	718	350	31429	30539	32351	29617	61968	48,75	1,09
Pavlov 1 (l)	709	357	30264	30687	31680	29271	60951	50,35	1,08
Sunghir 1 (r)	771	488	47674	36827	50724	33776	84500	63,29	1,50
Sunghir 4 (l)	683	457	41152	27675	41255	27573	68827	66,91	1,50

Data from [80]

Table S17. Cross-sectional geometric parameters of the Circeo 7 (C7) femur and comparison. Midshaft (50%) femoral diaphysial cross-sectional parameters. **Ta**, Total area (mm^2); **CA**, Cortical area (mm^2); **Ix** (mm^4), anterior-posterior bending rigidity; **Iy,** (mm^4) medio-lateral bending rigidity; **Imax,** (mm^4), maximum bending rigidity; **Imin**, (mm^4), minimum bending rigidity; **J**, polar second moment of area; **%CA**, Relative cortical thickness (percent cortical area= %CA = (CA/TA) × 100); **Imax/Imin**, diaphyseal circular index; **MPMH**, Middle Paleolithic modern human; **EUP/MUP**, Early and Mid-Upper Paleolithic; **(r)**, right; **(l)**, left.

Sample/Specimens	TA	CA	Ix	Iy	Imax	Imin	J	%CA	Imax/Imin
**Circeo7** (Original *data*)	**651**	**561**	**31306**	**36117**	**40082**	**27341**	**67423**	**86,21**	**1,47**
**Early Pleistocene**									
KNM-ER 736 (l)	871	659	56553	60075	65146	51482	116628	75,66	1,27
KNM-ER 737 (l)	586	441	21538	32802	33140	20200	54340	75,26	1,64
KNM-ER 803a (l)	626	504	27573	34793	38942	23424	62366	80,51	1,66
KNM-ER 1472 (r)	464	400	16191	17987	20429	13748	34178	86,21	1,49
KNM-ER 1481a (l)	391	332	10167	14416	14848	9734	24583	84,91	1,53
KNM-ER1808mn (r)	551	478	20813	27251	27330	20735	48064	86,75	1,32
**Middle Pleistocene**									
Aïn Maarouf 1 (l)	506	428	19718	20306	22010	18015	40024	84,58	1,22
Berg Aukas 1 (r)	800	708	51599	51050	61019	41631	102649	88,50	1,47

Broken Hill E690 (l)	468	420	19363	15999	21229	14134	35362	89,74	1,50
Broken Hill E793 (-)	587	428	26198	25272	28746	22724	51470	72,91	1,27
Castel del Guido 1 (r)	666	582	42003	29626	43024	28605	71629	87,39	1,50
La Chaise-BD 5 (r)	502	410	17883	21149	21561	17471	39032	81,67	1,23
Ehringsdorf 5 (r)	814	666	52616	49833	57005	45444	102449	81,82	1,25
Gesher-B.-Y. 1 (r)	373	316	8991	13418	13513	8896	22408	84,72	1,52
Kresna 11 (l)	591	454	23587	30202	30637	23149	53789	76,82	1,32
Mammolo 1 (r)	761	609	51832	37927	51847	37911	89759	80,03	1,37
OH 28 (l)	576	410	18731	31758	32379	18110	50489	71,18	1,79
**Sample/Specimens**	**TA**	**CA**	**Ix**	**Iy**	**Imax**	**Imin**	**J**	**%CA**	**Imax/Imin**
Tabun E12 (r)	577	544	24709	28951	31218	22442	53660	94,28	1,39
Zhoukoudian 1 (l)	572	497	22521	29366	29673	22215	51887	86,89	1,34
Zhoukoudian 2 (l)	453	413	13726	19686	19702	13710	33412	91,17	1,44
Zhoukoudian 4 (r)	515	428	17804	24029	24769	17064	41833	83,11	1,45
Zhoukoudian 5 (l)	470	420	14573	20717	20762	14528	35290	89,36	1,43
Zhoukoudian 6 (l)	502	440	19282	21367	24295	16354	40649	87,65	1,49
**Neanderthal**									
Amud 1 (l)	749	660	44164	46307	55455	35016	90471	88,12	1,58
Chapelle-aux-Saints 1 (l)	725	606	38088	43120	43178	38030	81208	83,59	1,14
Feldhofer 1 (r)	652	504	33955	30596	37384	27166	64551	77,30	1,38
Ferrassie 1 (r)	721	565	38744	42513	50953	30304	81257	78,36	1,68
Ferrassie 1 (l)	728	581	35191	46398	46829	34761	81590	79,81	1,35
Ferrassie 2 (r)	601	461	24392	30914	32334	22971	55305	76,71	1,41
Ferrassie 2 (l)	627	482	27378	32515	35561	24332	59893	76,87	1,46
Fond-de-Forêt 1 (l)	727	571	40732	41154	48582	33305	81886	78,54	1,46
Palomas 96 (r)	429	284	14158	15017	16739	12437	29175	66,20	1,35
Quina 5 (r)	618	537	29356	30141	30386	29111	59497	86,89	1,04
Rochers-de-V. 1 (l)	646	545	34007	30948	34057	30898	64955	84,37	1,10
Saint-Césaire 1 (r)	740	624	47905	38670	52020	34555	86575	84,32	1,51
Shanidar 4 (r)	768	610	46120	43883	47274	42729	90003	79,43	1,11
Shanidar 5 (r)	813	620	50193	49511	52327	47376	99704	76,26	1,10
Shanidar 6 (r)	548	414	21399	23735	23800	21333	45133	75,55	1,12
Spy 2 (r)	616	493	27664	30579	31873	26371	58243	80,03	1,21
Tabun 1 (r)	497	427	16166	23002	23105	16063	39168	85,92	1,44
Tabun 3 (r)	390	328	11012	12960	14070	9902	23972	84,10	1,42
**MPMH**									
Qafzeh 3 (l)	576	438	30596	21246	30621	21221	51842	76,04	1,44
Qafzeh 8 (r)	775	636	66853	35575	67446	34981	102428	82,06	1,93
Qafzeh 9 (r)	770	624	53769	40845	54488	40126	94614	81,04	1,36
Skhul 3 (l)	708	523	42627	34322	42986	33963	76949	73,87	1,27
Skhul 4 (r)	598	468	33312	23360	35271	21401	56672	78,26	1,65
Skhul 4 (l)	563	476	32067	20664	32860	19871	52731	84,55	1,65
Skhul 5 (r)	760	525	58890	32473	60811	30552	91363	69,08	1,99
Skhul 5 (l)	691	528	55730	25144	55776	25098	80874	76,41	2,22
Skhul 6 (r)	606	481	36153	22770	36963	21960	58923	79,37	1,68
Skhul 7 (r)	520	403	18906	22599	23880	17625	41505	77,50	1,35
**EUP/MUP**									
Arene Candide 1 (r)	569	309	26000	15500	26000	15500	41500	54,31	1,68
Barma Grande 2 (l)	737	603	53900	33800	54500	33200	87700	81,82	1,64
Cro-Magnon 1 (r)	790	642	71055	38435	71985	37505	109490	81,27	1,92

Cro-Magnon 1 (l)	732	581	52695	37038	53069	36664	89733	79,37	1,45
Cro-Magnon 4322 (l)	555	476	33207	18635	33607	18235	51842	85,77	1,84
Cro-Magnon 4324 (l)	541	432	28345	18966	28477	18834	47311	79,85	1,51
Dolní Věstonice 3 (r)	411	307	14539	11552	15495	10596	26091	74,70	1,46
Dolní Věstonice 3 (l)	406	306	14290	11054	14348	10996	25343	75,37	1,30
Dolní Věstonice 13 (r)	574	429	27986	22501	28842	21646	50487	74,74	1,33
Dolní Věstonice 13 (l)	557	401	23190	22898	23519	22568	46087	71,99	1,04
Dolní Věstonice 14 (r)	551	389	26759	19015	26790	18984	45774	70,60	1,41
Dolní Věstonice 14 (l)	552	404	29908	17673	29933	17648	47581	73,19	1,70
**Sample/Specimens**	**TA**	**CA**	**Ix**	**Iy**	**Imax**	**Imin**	**J**	**%CA**	**Imax/Imin**
Dolní Věstonice 16 (r)	614	422	36190	22041	36444	21787	58231	68,73	1,67
Dolní Věstonice 16 (l)	624	422	36490	22762	36704	22547	59251	67,63	1,63
Dolní Věstonice 35 (r)	588	384	33208	20320	35131	18397	53528	65,31	1,91
Grotte-des-Enfants 4(r)	717	545	52300	31400	52300	31400	83700	76,01	1,67
Minatogawa 1 (r)	508	385	19190	19900	20590	18500	39090	75,79	1,11
Minatogawa 1 (l)	499	401	19620	18950	20150	18420	38570	80,36	1,09
Minatogawa 2 (l)	350	257	8460	9800	9800	8450	18260	73,43	1,16
Minatogawa 1(r)	390	354	13110	11500	13120	11490	24610	90,77	1,14
Minatogawa 1(l)	391	365	12320	12460	12910	11870	24780	93,35	1,09
Minatogawa 4 (r)	327	236	8040	7880	8530	7390	15920	72,17	1,15
Mladeč 27 (r)	498	391	23588	15493	24169	14912	39081	78,51	1,62
Nahal 'En-Gev 1 (l)	455	390	18328	14981	18453	14856	33308	85,71	1,24
Ohalo 2 (r)	660	506	47715	25000	49490	23225	72715	76,67	2,13
Ohalo 2 (l)	641	518	43365	24777	44031	24111	68142	80,81	1,83
Paglicci 25 (r)	615	482	34900	24300	34900	24300	59200	78,37	1,44
Paviland 1 (l)	608	466	34298	22793	34466	22625	57091	76,64	1,52
Pavlov 1 (r)	611	411	30665	25570	32324	23912	56235	67,27	1,35
Pavlov 1 (l)	633	423	28040	30871	34266	24645	58911	66,82	1,39
Rochette 2 (r)	524	443	23200	20700	24400	19500	43900	84,54	1,25
Sunghir 1 (r)	788	591	57640	40076	60680	37036	97716	75,00	1,64
Sunghir 1 (l)	678	518	40882	34264	48086	27060	75146	76,40	1,78
Sunghir 4 (l)	622	462	34894	24437	34914	24418	59331	74,28	1,43
Veneri 1 (l)	767	617	61300	36000	61400	35900	97300	80,44	1,71
Veneri 2 (l)	671	543	44100	28700	44300	28400	72800	80,92	1,56
Willendorf 1 (r)	477	375	20811	15249	21036	15025	36060	78,62	1,40
Zhoukoudian-UC 67 (r)	728	577	67611	26028	67917	25723	93639	79,26	2,64
Zhoukoudian-UC 68 (r)	634	512	45154	21576	45156	21575	66730	80,76	2,09

Data from [80]

Table S18. Cross-sectional geometric parameters of the Circeo 7 (C7) femur and comparison. Mid-proximal (65%) femoral diaphysial cross-sectional parameters. **Ta**, Total area (mm^2); **CA**, Cortical area (mm^2); **Ix** (mm^4), anterior-posterior bending rigidity; **Iy,** (mm^4) medio-lateral bending rigidity; **Imax,** (mm^4), maximum bending rigidity; **Imin**, (mm^4), minimum bending rigidity; **J**, polar second moment of area; **MPMH**, Middle Paleolithic modern human; **EUP/MUP**, Early and Mid-Upper Paleolithic; **(r)**, right; **(l)**, left.

Sample/Specimens	TA	CA	Ix	Iy	Imax	Imin	J
**Circeo7** (Original *data*)	**671**	**635**	**42833**	**30297**	**44300**	**28830**	**73130**
**Early Pleistocene**							

KNM-ER 736 (l)	890	712	56200	65999	66775	55425	122199
KNM-ER 737 (l)	654	523	27809	40273	41382	26701	68082
KNM-ER 803a (l)	657	550	28547	39918	40372	28094	68465
KNM-ER 1472 (r)	492	427	17252	20981	21344	16889	38233
KNM-ER 1481° (l)	450	384	11803	21137	21191	11749	32940
KNM-ER 1808mn (r)	574	502	18360	37183	37216	18327	55543
**Middle Pleistocene**							
Aïn Maarouf 1 (l)	503	457	19062	21129	22388	17800	40188
Arago 48 (r)	634	587	--	--	34420	24029	58449

Sample/Specimens	TA	CA	Ix	Iy	Imax	Imin	J
Broken Hill E690 (l)	486	454	15882	22250	22438	15694	38132
Gesher-B.-Y. 1 (r)	404	336	9405	16959	17014	9350	26364
Kresna 11 (l)	643	496	25850	39454	39864	25397	65304
OH 28 (l)	622	451	20122	40586	40775	19932	60708
Tabun E1 (r)	575	543	23317	29558	29558	23317	52875
**Neanderthal**							
Amud 1 (r)	770	701	42100	52267	52697	41670	94367
Feldhofer 1 (r)	682	556	33025	38528	39437	32116	71553
Ferrassie 1 (r)	749	625	36695	51254	52401	35548	87949
Ferrassie 1 (l)	711	576	34341	43637	43859	34119	77978
Ferrassie 2 (r)	616	474	25807	32332	33931	24208	58139
Ferrassie 2 (r)	613	448	24972	30439	30555	24857	55412
Fond-de-Forêt 1 (l)	748	585	38637	46822	48072	37387	85459
Krapina 257.32 (l)	632	565	22346	44428	44502	22272	66774
Krapina 257.33 (l)	564	537	20671	33537	36744	17463	54208
Palomas 52 (l)	386	327	11800	11423	12294	10929	23223
Palomas 92 (r)	534	454	25935	19222	26499	18657	45157
Palomas 96 (r)	415	366	12402	14771	14960	12213	27173
Quina 38 (l)	591	530	24229	32584	33657	23155	56813
Shanidar 6 (l)	542	484	20116	26606	26887	19834	46722
Spy 2 (r)	608	521	29654	29954	37854	21754	59608
**MPMH**							
Qafzeh 6 (r)	758	603	47331	39477	50246	36562	86808
Qafzeh 8 (r)	768	646	55184	38847	55760	38271	94031
Qafzeh 9 (r)	696	664	43534	34996	44032	34498	78530
Skhul 4 (r)	582	525	27678	26044	28621	25101	53722
Skhul 4 (l)	558	503	25916	23483	26000	23400	49400
Skhul 5 (r)	740	523	45599	33883	49629	30353	79481
**EUP/MUP**							
Cro-Magnon 1(r)	798	695	61856	40435	61921	40370	102291
Cro-Magnon 1(l)	797	702	55864	45166	58061	42968	101029
Cro-Magnon 4322 (l)	599	510	28532	28347	33127	23752	56879
Cro-Magnon 4324 (l)	556	473	26011	23069	29817	19263	49080
Dolní Věstonice 3 (r)	406	321	11195	14236	14348	11083	25431
Dolní Věstonice 3 (l)	383	317	10560	12397	12423	10533	22956
Dolní Věstonice 13 (r)	557	437	24110	23194	25518	21786	47304
Dolní Věstonice 13 (l)	549	456	21672	25492	27599	19565	47164
Dolní Věstonice 14 (r)	584	442	27946	24549	32716	19778	52495
Dolní Věstonice 14 (l)	564	444	29987	19696	30527	19155	49683
Dolní Věstonice 16 (r)	625	428	31927	25075	31988	25014	57002

Dolní Věstonice 16 (l)	626	443	33880	24457	34191	24146	58337
Dolní Věstonice 41 (r)	517	439	17712	24431	24483	17660	42143
Minatogawa 1 (r)	509	421	18340	22340	23030	17650	40680
Minatogawa 1 (l)	517	435	21100	21310	24280	18130	42410
Minatogawa 3 (r)	393	359	11940	13010	14970	9970	24940
Minatogawa 3 (l)	395	379	11290	13950	14760	10480	25240
Minatogawa 4 (r)	354	284	8430	11080	11540	7970	19510
Mladeč 27 (r)	504	397	20056	18636	20676	18016	38692
Mladeč 28 (l)	496	409	16469	21937	22443	15963	38406
**Sample/Specimens**	**TA**	**CA**	**Ix**	**Iy**	**Imax**	**Imin**	**J**
Nahal 'En-Gev 1 (l)	463	403	17615	16223	18490	15348	33838
Ohalo 2 (r)	628	511	36720	26594	39485	23730	63215
Ohalo 2 (l)	633	529	35627	27772	37627	25772	63399
Paviland (l)	613	527	31823	27646	34402	25068	59470
Pavlov 1 (r)	636	497	32681	28904	32706	28879	61585
Pavlov 1 (l)	644	508	29765	33473	34172	29066	63238
Sunghir 1 (r)	767	612	39549	51697	52000	39246	91246
Sunghir 1 (l)	714	608	38775	41271	41649	38397	80046
Sunghir 4 (l)	681	497	38369	30553	39815	29107	68922
Tianyuan 1 (r)	717	618	48760	33651	49232	33178	82410
Tianyuan 1 (l)	676	559	46314	28334	47001	27648	74649
Willendorf 1 (r)	506	431	20714	19938	22413	18238	40652

Data from [80]

Table S19. Cross-sectional geometric parameters of the Circeo 7 (C7) femur and comparison. Proximal (80%) femoral diaphysial cross-sectional parameters. **Ta**, Total area (mm^{2}); **CA**, Cortical area (mm^2); **Ix** (mm^4), anterior-posterior bending rigidity; **Iy,** (mm^4) medio-lateral bending rigidity; **Imax,** (mm^4), maximum bending rigidity; **Imin**, (mm^4), minimum bending rigidity; **J**, polar second moment of area; **MPMH**, Middle Paleolithic modern human; **EUP/MUP**, Early and Mid-Upper Paleolithic; **(r)**, right; **(l)**, left.

Sample/Specimens	TA	CA	Ix	Iy	Imax	Imin	J
**Circeo7** (Original *data*)	**773**	**661**	**58472**	**37731**	**59263**	**36940**	**96203**
**Early Pleistocene**							
KNM-ER 736 (l)	857	656	41747	72382	72409	41720	114129
KNM-ER 737 (l)	738	510	29110	55466	58568	26008	84576
KNM-ER 803a (l)	706	542	30943	47470	50706	27707	78413
KNM-ER 1472 (r)	502	424	13576	28666	29174	13068	42242
KNM-ER 1481 (l)	529	414	16151	28797	30018	14930	44948
KNM-ER 1808mn (r)	629	498	24559	43721	50333	17947	68280
**Middle Pleistocene**							
Berg Aukas 1 (r)	842	744	--	--	75604	42288	117892
Broken Hill E689 (l)	620	532	27573	32583	33324	26832	60156
Broken Hill E690 (l)	527	467	17338	30563	33602	14300	47901
Broken Hill E709 (r)	796	698	43443	55320	55984	42778	98763
La Chaise-BD 5 (r)	532	411	20523	23443	27320	16646	43966
Gesher-B.–Y. 1 (r)	471	346	13136	21097	22221	12012	34233
Kresna 11 (l)	454	693	26536	44846	45131	26311	71382
OH 28 (l)	685	446	22850	49292	50717	21425	72142
Tabun E1 (r)	580	545	24905	30665	35402	20168	55570
**Neanderthal**							

Amud 1(r)	803	699	47325	56044	62757	40612	103369
Amud 1(l)	877	679	57149	63185	76194	44141	120335
Chapelle-aux-Saints 1(r)	797	634	46481	51455	51467	46469	97936
Feldhofer 1(r)	758	594	41631	45609	48168	39072	87240
Ferrassie 1(r)	896	664	49388	73998	76960	46426	123386
Ferrassie 1(l)	836	574	46154	56283	60604	41832	102436
Ferrassie 2 (l)	719	474	34570	38803	43119	30254	73373
Krapina 213 (l)	763	663	34880	61419	63574	32725	96298
Krapina 214 (l)	489	431	13614	27100	27302	13412	40714
**Sample/Specimens**	**TA**	**CA**	**Ix**	**Iy**	**Imax**	**Imin**	**J**
Saint-Césaire 1(r)	783	550	40181	49949	51085	39045	90129
Spy 2 (r)	970	573	57762	69356	69799	57319	127118
Tabun 1(r)	557	445	20145	28546	29411	19281	48691
**MPMH**							
Qafzeh 8 (r)	827	688	40095	73059	77914	35240	113154
Qafzeh 9 (r)	672	578	36644	34288	40576	30356	70932
Skhul 4 (r)	662	501	28225	39076	39980	27321	67301
Skhul 4 (l)	626	477	29111	32668	38922	22857	61779
Skhul 5 (r)	745	493	41908	36680	41910	36678	78588
Skhul 6 (l)	590	457	26484	26847	31272	22058	53330
Skhul 9 (l)	814	552	32638	68171	68756	32053	100809
Arene Candide 1 (r)	595	417	19500	33500	33600	19400	53000
Barma Grande 2 (l)	844	685	46200	75500	88700	33000	121700
Cro-Magnon 1 (r)	842	695	50228	63339	70577	42989	113566
Cro-Magnon 1 (l)	789	593	41151	57052	64488	33715	98203
Cro-Magnon 4322 (l)	660	497	28093	43204	48184	23113	71297
Dolní Věstonice 3 (r)	451	329	12075	20882	23231	9726	32957
Dolní Věstonice 3 (l)	441	340	10536	22260	23695	9101	32796
Dolní Věstonice 13 (r)	635	491	25173	38569	40786	22956	63741
Dolní Věstonice 13 (l)	586	477	22935	36349	42611	16673	59284
Dolní Věstonice 14 (r)	653	438	22813	45364	49342	18835	68176
Dolní Věstonice 14 (l)	662	434	26275	42119	48043	20352	68395
Dolní Věstonice 16(r)	688	456	32254	37263	44033	25483	69517
Dolní Věstonice 16 (l)	680	434	34065	33121	43623	23563	67186
Dolní Věstonice 35 (r)	585	473	23306	33261	37566	19001	56567
Grotte-des-Enfants 4 (r)	761	563	34200	56600	57800	33000	90800
Minatogawa 1 (r)	520	428	18891	23855	25710	17036	42746
Minatogawa 1 (l)	546	454	20878	26612	29305	18186	47491
Minatogawa 2 (l)	356	269	7935	12162	13393	6705	20097
Minatogawa 3 (r)	376	339	8851	14936	15398	8039	23437
Minatogawa 3 (l)	393	348	9952	15746	16838	8860	25698
Minatogawa 4 (r)	366	307	8587	13349	14357	7579	21936
Mladeč 27 (r)	597	390	21089	29263	29325	21027	50352
Mladeč 28 (l)	574	427	21049	32743	37190	16602	53792
Nahal ‘En-Gev 1 (l)	475	370	13300	22439	22960	12779	35739
Ohalo 2 (r)	642	461	26513	35881	39119	23276	62395
Ohalo 2 (l)	632	481	26588	35402	39419	22572	61991
Paglicci 25 (r)	615	548	23600	39500	41800	21300	63100
Paviland 1 (l)	681	482	32212	41073	50430	22856	73286
Pavlov 1 (r)	706	458	28507	47136	52435	23209	75643

Rochette 2 (r)	651	508	29500	36400	40500	25400	65900
Sunghir 1 (r)	793	548	42781	56920	70613	29089	99702
Sunghir 1 (l)	810	579	49008	51237	64451	35795	100246
Sunghir 4 (l)	700	478	33430	38699	43884	28245	72129
Tianyuan 1 (r)	752	637	39607	49206	49674	39139	88813
Tianyuan 1 (l)	716	589	36648	43131	44526	35253	79779
Veneri 1 (l)	843	648	53900	55900	68300	41500	109800
Veneri 2 (l)	805	623	51600	50000	64500	37100	101600

Data from [80]
